# Design, Synthesis and Antitumor Evaluation of Novel Pyrazolopyrimidines and Pyrazoloquinazolines

**DOI:** 10.3390/molecules23061249

**Published:** 2018-05-23

**Authors:** Mohamed El-Naggar, Ashraf S. Hassan, Hanem M. Awad, Mohamed F. Mady

**Affiliations:** 1 Chemistry Department, Faculty of Sciences, University of Sharjah, Sharjah 27272, UAE; M5elnaggar@yahoo.com; 2 Organometallic and Organometalloid Chemistry Department, National Research Centre, Dokki, Cairo 12622, Egypt; 3 Department of Tanning Materials and Leather Technology, National Research Centre, Dokki, Cairo 12622, Egypt; hanem_awad@yahoo.com; 4 Department of Green Chemistry, National Research Centre, Dokki, Cairo 12622, Egypt; 5 Deaprtment of Chemistry, Bio Science and Environmental Technology, Faculty of Science and Technology, University of Stavanger, N-4036 Stavanger, Norway

**Keywords:** pyrazolopyrimidines, pyrazoloquinazolines, synthesis, antitumor activity, cell cycle analysis

## Abstract

A series of *N*-aryl-7-aryl-pyrazolo[1,5-*a*]pyrimidines **18a**–**u** and *N*-aryl-pyrazolo[1,5-*a*]quinazolines **25a**–**c** were designed and synthesized via the reaction of 5-aminopyrazoles **11a**–**c** with enaminones **12a**–**g** or **19**, respectively. The new compounds were screened for their in vitro antitumor activity toward liver (HepG-2) and breast (MCF-7) human cancer cells using 3-[4,5-dimethyl-2-thiazolyl)-2,5-diphenyl-2*H*-tetrazolium bromide MTT assay. From the results, it was found that all compounds showed dose-dependent cytotoxic activities against both HepG-2 and MCF-7 cells. Two compounds **18o** and **18a** were selected for further investigations. Cell cycle analysis of liver (HepG-2) cells treated with **18o** and breast (MCF-7) cells treated with **18a** showed cell cycle arrest at G2/M phase and pro-apoptotic activity as indicated by annexin V-FITC staining.

## 1. Introduction

**Figure 1:**
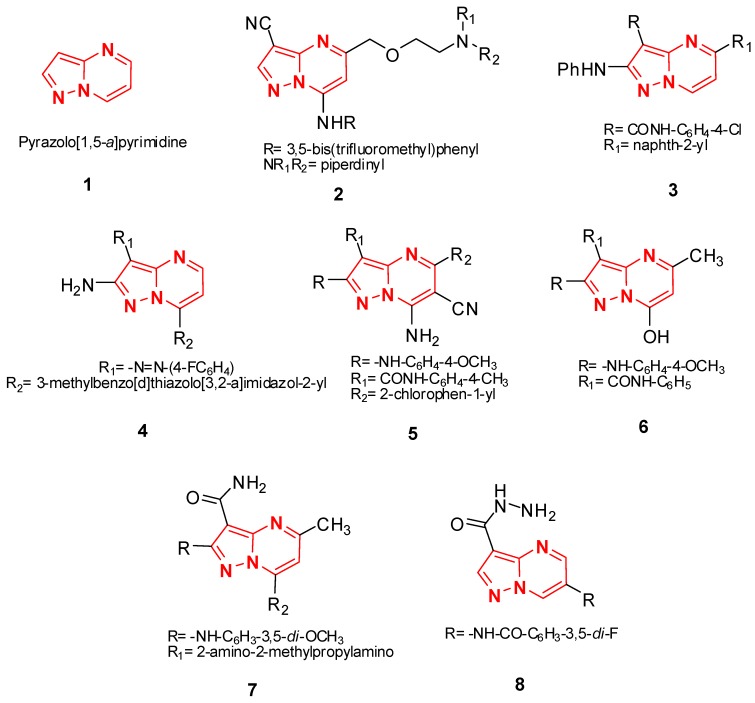
Structures of the antitumor activity compounds **1**–**6** and enzymes inhibitors **7**–**8**.

Pyrazolo[1,5-*a*]pyrimidine ring **1** and its derivatives occupy a unique place in medicinal chemistry due to its various pharmacological activities [1,2,3,4,5,6] especially antitumor properties [7,8,9]. In 2006, Li et al. synthesized compound **2** which exhibited significant in vitro antitumor activity against Bel-7402 (liver) and HT-1080 (fibrosarcoma) cell lines [10]. In 2009, Ahmed et al., prepared compound **3** which was more effective and exhibited cytotoxicity against HCT116 (colon) and HeLa (cervix) cell lines [11]. In 2010, Abdel-Aziz and co-workers have described a facile synthesis of compound **4** which exhibited promising in vitro antitumor activity against CaCo-2 (colon) and BHK (normal fibroblast) cell lines [12]. Furthermore, we have reported the synthesis of compounds **5** and **6** in high yield by treating 5-aminopyrazole with 2-(2-chlorobenzylidene)malononitrile and ethyl acetoacetate, respectively, these compounds show good antitumor activities against HCT-116 and HepG2 cells [13,14] (Figure 1).

In addition, pyrazolo[1,5-*a*]pyrimidine derivatives have been reported as potent enzymes inhibitors [15,16,17]. Mukaiyama et al.*,* have prepared compound **7** which exhibited potent inhibitory activity against c-Src Kinase and good CNS penetration [18]. Very recently, Kumar et al., have prepared pyrazolo[1,5-*a*]pyrimidine carboxamide **8** which showed good aurora kinase A and B activity [19] (Figure 1).

**Figure 2:**
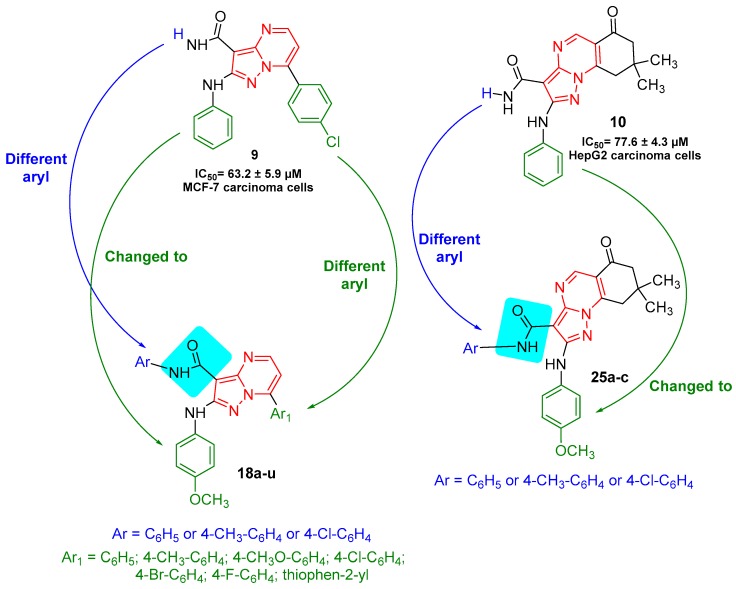
Design of novel *N*-aryl-pyrazolo[1,5-*a*]pyrimidines **18a**–**u** and *N*-aryl-pyrazolo[1,5-*a*]quinazolines **25a**–**c**-based amide linkages.

In continuation of our research program [20,21,22,23,24,25,26,27] and following the potent biological activity results against MCF-7 and HepG2 carcinoma cells which were obtained from our synthesized compounds such as 7-(4-chlorophenyl)-2-(phenylamino)pyrazolo[1,5-*a*]pyrimidine (**9**, IC_50_ = 63.2 ± 5.9 µM) and 2-(phenylamino)-pyrazolo[1,5-*a*]quinazoline (**10**, IC_50_ = 77.6 ± 4.3 µM) compared to doxorubicin [28]. In this work, we have planned to modify the pyrazolo[1,5-*a*]pyrimidine **9** and pyrazolo[1,5-*a*]quinazoline **10** to obtain *N*-aryl-7-aryl-pyrazolo[1,5-*a*]pyrimidines **18a**–**u** and *N*-aryl-pyrazolo[1,5-*a*]quinazolines **25a**–**c**, respectively, incorporating different aryl groups (blue and green) into the structures to evaluate their in vitro antitumor activities against HepG-2 and MCF-7 human cells to find novel and potent antitumor compounds (Figure 2).

## 2. Results and Discussion

### 2.1. Chemistry

**Scheme 1:**
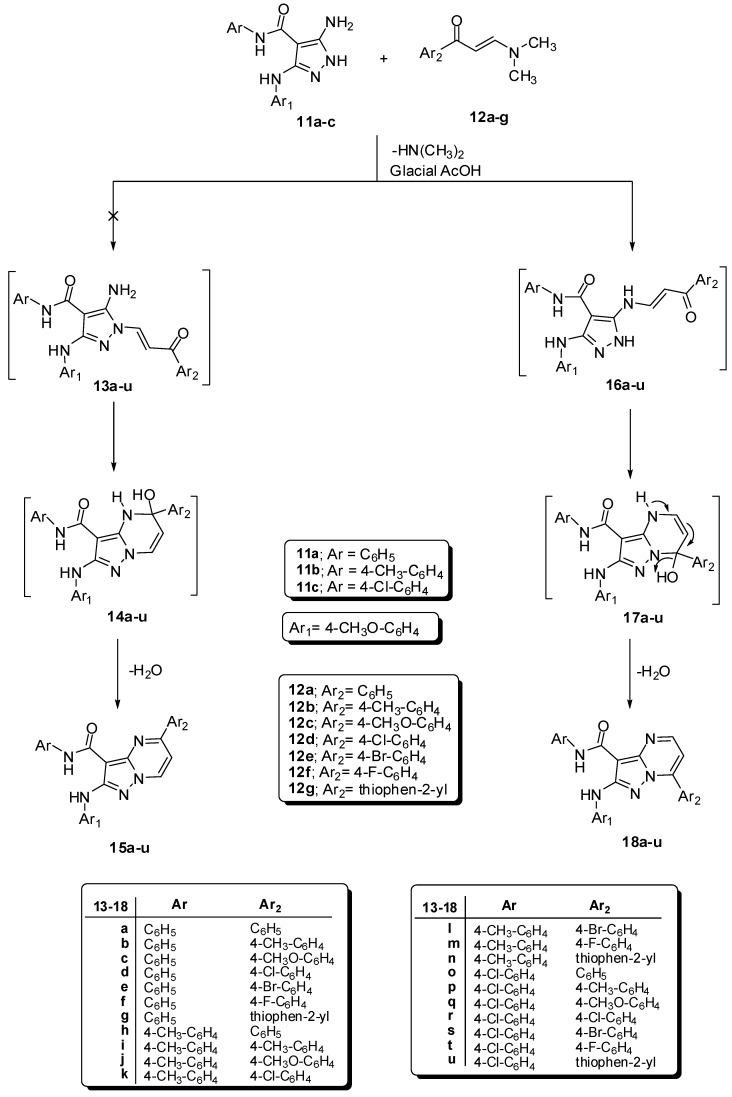
Synthesis of 7-aryl-pyrazolo[1,5-*a*]pyrimidines **18a**–**u**.

**Scheme 2:**
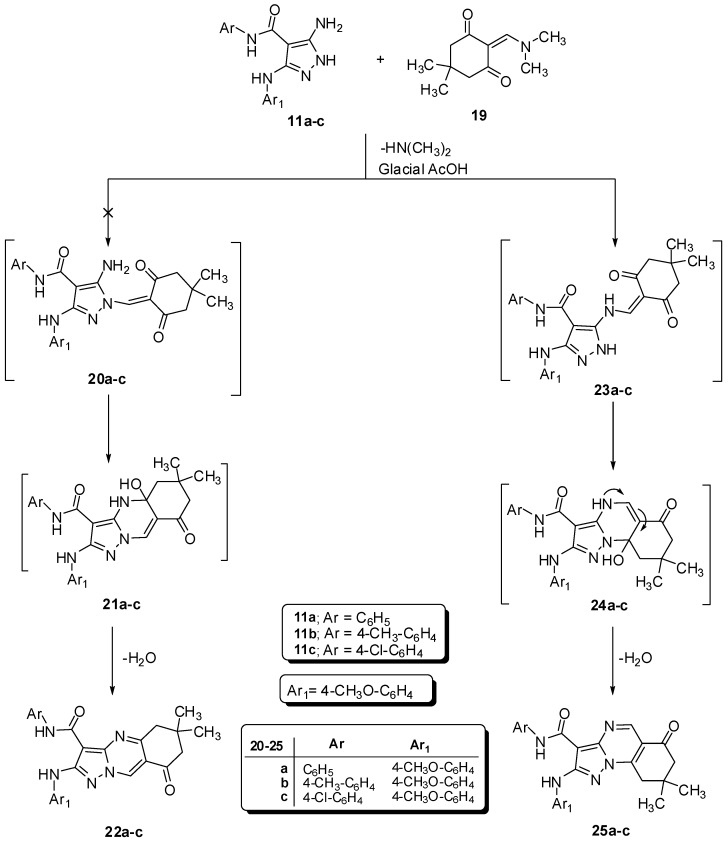
Synthesis of *N*-aryl-2-(arylamino)-pyrazolo[1,5-*a*]quinazolines **25a**–**c**.

The syntheses oftarget compounds **18a**–**u** and **25a**–**c** are illustrated in Scheme 1 and Scheme 2. The starting materials, 5-amino-*N*-aryl-1*H*-pyrazole-4-carboxamides **11a**–**c** were synthesized according to our previous work [29]. Reaction of compounds **11a**–**c** with 1-(aryl)-3-(dimethylamino)prop-2-en-1-ones **12a**–**g** in glacial acetic acid furnished one isolable product 5-aryl-pyrazolo[1,5-*a*]pyrimidines **15a**–**u** or 7-aryl-pyrazolo[1,5-*a*]pyrimidines **18a**–**u**. As depicted in Scheme 1, the final products were confirmed by the spectral analysis.

The ^1^H-NMR spectrum (CDCl_3_, *δ* ppm) exhibited, in each case **18c** or **15c**, characteristic two doublets of the pyrimidine protons at 6.87 (1H, H-6) and at 8.40 (1H, H-5) (each with *J* = 8.4 Hz) and four signals at 3.80, 3.91, 9.36 and 10.02 due to 2OCH_3_ and 2NH, respectively. The ^13^C-NMR spectrum (CDCl_3_, *δ* ppm), in each case **18c** or **15c**, also characterized by signals of –OCH_3_, –OCH_3_, C_3_-pyrazolopyrimidine, C_6_-pyrazolopyrimidine, C_5_-pyrazolopyrimidine and C=O at 55.53, 55.60, 87.45, 106.43, 157.52 and 163.21, respectively.

Although, ^1^H- and ^13^C-NMR spectra cannot differentiate between **15a**–**u** and **18a**–**u**, ^1^H-^15^N HMBC spectrum used for differentiating between the two isomers. The ^1^H-^15^N HMBC spectrum of the final product shows the most important correlated coupling between the proton H-5 of pyrazolopyrimidine (^1^H, at 8.40 ppm) with N-4 of pyrimidine (^15^N, at 255 ppm) ^2^*J* (H-5, N-4) gave absolute confirmation for the structure of **18a**–**u** and conclude **15a**–**u** (cf. Supporting Information). Also, the structure of **18a**–**u** was supported by X-ray crystallography of similar analogs and products [12].

In addition, *N*-aryl-2-(arylamino)-pyrazolo[1,5-*a*]quinazolines **25a**–**c** were formed by the condensation of **11a**–**c** with 2-((dimethylamino)methylene)-5,5-dimethylcyclohexane-1,3-dione (**19**) in a glacial AcOH (Scheme 2) while pyrazolo[1,5-*a*]quinazolines **22a**–**c** were not formed. The spectral analysis of the products supported the structures of **25a**–**c**.

**Figure 3:**
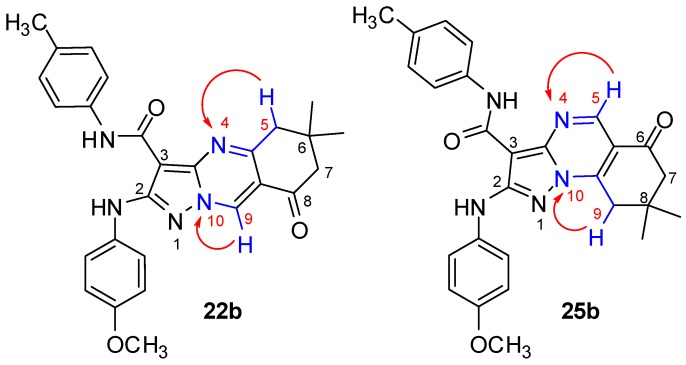
Diagnostic correlations in the ^1^H-^15^N HMBC spectrum for the two isomers **22b** and **25b**.

The mass spectrum of **25b** confirmed the molecular formula C_27_H_27_N_5_O_3_ (469.53) {MS (*m*/*z*, %): 469 (M^+^, 93.88)}. The ^1^H-NMR (CDCl_3_, 400 MHz, *δ* ppm) spectrum of **25b** was characterized by sharp signals of 2CH_3_, CH_2_ and CH_2_ groups of the dimedone at 1.19, 2.52 and 3.22, respectively. The OCH_3_ group, H-5 proton of quinazoline and 2NH protons appeared as singlet signals at 3.81, 8.90, 9.41 and 9.72, respectively. The aromatic protons of 4-methoxyphenylamino ring appeared as two doublets at 6.91 (2*H*) and 7.58 (2*H*) with the coupling constant *J* = 9.0 Hz and the four aromatic protons of *N*-(4-methylphenyl) ring appeared as two doublets at 7.16 (*J* = 8.2 Hz) and 7.54 (*J* = 8.3 Hz). Also, the ^13^C-NMR (CDCl_3_, 100 MHz, *δ* ppm) spectrum showed characteristic signals at 28.70 corresponding to 2CH_3_, at 32.65 for a C_8_ (quinazoline), two signals at 37.52 and 50.99 corresponding to 2CH_2_ and signal at 194.01 due to C=O (quinazoline). The ^1^H-^15^N HMBC spectrum showed that the two most important correlated coupling which gave absolute and unique confirmation for the structure of **25a**–**c**, the first was between the proton H-5 of quinazoline (^1^H, at 8.90 ppm) with N-4 of quinazoline (^15^N, at 260 ppm) ^2^*J* (H-5, N-4) and the second was between the CH_2_ of quinazoline (^1^H, at 3.22 ppm) with N-10 of quinazoline (^15^N, at 216 ppm) ^3^*J* (H-9, N-10) (cf. Supporting Information) (Figure 3).

If the compound **22b** was obtained, its ^1^H-^15^N HMBC spectrum would have exhibited that correlated coupling between the proton H-9 of quinazoline with N-10 of quinazoline ^2^*J* (H-9, N-10) and correlated coupling between the CH_2_ of quinazoline with N-4 of quinazoline ^3^*J* (H-5, N-4), but, these two correlated coupling were not detected in ^1^H-^15^N HMBC spectrum (Figure 3). Furthermore, X-ray diffraction of similar analogs added unequivocal evidence for the structures of **25a**–**c** and confirmed the reaction mechanism [30].

### 2.2. In Vitro Cytotoxic Activity

For evaluation of in vitro cytotoxic activity of compounds {5-aminopyrazoles **11a**–**c**, pyrazolo[1,5-*a*]pyrimidines **18a**–**u** and pyrazolo[1,5-*a*]quinazolines **25a**–**c**} against liver (HepG-2) and breast (MCF-7) human carcinoma cell lines, MTT assay was used [31,32,33]. Doxorubicin^®^ was used as a reference cytotoxic compound. The results were expressed as growth inhibitory concentration (IC_50_) values (Table 1).

From the results of in vitro cytotoxic activity, it was found that most of the prepared compounds displayed comparable IC_50_ values against liver (HepG-2) and breast (MCF-7) cancer cell lines compared to positive control.

For HepG-2 cancer cells, most of the tested compounds did not show any significant difference compared to the positive control. Only four compounds (**11c**, **18b**, **18f** and **18g**) showed significant difference in their activities. Compounds **18c** (IC_50_ = 75.9 ± 5.3 µM), **18d** (IC_50_ = 77.1 ± 4.2 µM), **18h** (IC_50_ = 73.2 ± 3.2 µM), **18j** (IC_50_ = 77.4 ± 2.9 µM), **18k** (IC_50_ = 74.0 ± 3.1 µM), **18l** (IC_50_ = 78.7 ± 5.1 µM), **18o** (IC_50_ = 72.2 ± 3.8 µM), **18q** (IC_50_ = 72.8 ± 3.9 µM), **18r** (IC_50_ = 73.0 ± 1.9 µM), **18s** (IC_50_ = 78.2 ± 3.2 µM), **18t** (IC_50_ = 78.7 ± 4.7 µM) and **25c** (IC_50_ = 79.5 ± 4.8 µM) showed slightly higher activities than doxorubicin (IC_50_ = 80.9 ± 2.1 µM). In addition, compound **18m** (IC_50_ = 80.3 ± 3.9 µM) was almost equipotent as doxorubicin (IC_50_ = 80.9 ± 2.1 µM), while, compounds **11a** (IC_50_ = 81.3 ± 4.1 µM), **18e** (IC_50_ = 81.2 ± 5.5 µM), **18n** (IC_50_ = 82.5 ± 5.7 µM) and **25b** (IC_50_ = 81.9 ± 5.9 µM) displayed slightly less activities compared to doxorubicin (IC_50_ = 80.9 ± 2.1 µM).

In case of MCF-7 cell lines, none of thecompounds showed any significant differences compared to the positive control. Compounds **18a** (IC_50_ = 63.1 ± 3.1 µM), **18b** (IC_50_ = 64.9 ± 3.1 µM), **18c** (IC_50_ = 64.3 ± 4.2 µM), **18j** (IC_50_ = 64.3 ± 3.1 µM), **11o** (IC_50_ = 64.7 ± 1.9 µM) and **18u** (IC_50_ = 64.5 ± 2.9 µM) showed slightly higher activities than doxorubicin (IC_50_ = 65.6 ± 4.2 µM). Whilst, compounds **11a** (IC_50_ = 65.5 ± 4.3 µM), **18d** (IC_50_ = 65.1 ± 2.8 µM), **18n** (IC_50_ = 65.9 ± 3.1 µM), **18q** (IC_50_ = 65.5 ± 2.1 µM) and **18r** (IC_50_ = 65.9 ± 2.6 µM) displayed equipotent as doxorubicin (IC_50_ = 65.6 ± 4.2 µM). Whereas, compounds **18f** (IC_50_ = 66.1 ± 2.9 µM), **18h** (IC_50_ = 66.8 ± 2.6 µM), **18k** (IC_50_ = 66.8 ± 3.9 µM), **18l** (IC_50_ = 66.7 ± 3.2 µM), **18m** (IC_50_ = 66.2 ± 3.8 µM), **18s** (IC_50_ = 66.8 ± 5.0 µM), **25b** (IC_50_ = 66.2 ± 2.9 µM) and **25c** (IC_50_ = 66.5 ± 3.1 µM) displayed slightly less activities.

### 2.3. Structure Activity Relationship (SAR)

From the results of in vitrocytotoxic activity of the synthesized compounds against liver (HepG2) cell lines, we found that, **25c** (IC_50_ = 79.5 ± 4.8 µM) >**25b** (IC_50_ = 81.9 ± 5.9 µM) >**25a** (IC_50_ = 87.9 ± 6.0 µM) in the series of pyrazolo[1,5-*a*]quinazolines **25a**–**c**, in addition, **18o** (IC_50_ = 72.2 ± 3.8 µM) >**18h** (IC_50_ = 73.2 ± 3.2 µM) >**18a** (IC_50_ = 85.4 ± 5.1 µM); **18r** (IC_50_ = 73.0 ± 1.9 µM) >**18k** (IC_50_ = 74.0 ± 3.1 µM) >**18d** (IC_50_ = 77.1 ± 4.2 µM); **18s** (IC_50_ = 78.2 ± 3.2 µM) >**18l** (IC_50_ = 78.7 ± 5.1 µM) >**18c** (IC_50_ = 75.9 ± 5.3 µM) and **18t** (IC_50_ = 78.7 ± 4.7 µM) >**18m** (IC_50_ = 80.3 ± 3.92 µM) >**18f** (IC_50_ = 92.8 ± 6.7 µM) in the series of pyrazolo[1,5-*a*]pyrimidines **18a**–**u**. This was concerning the effect of 4-Cl-C_6_H_4_ group (chloride atom as electron withdrawing group) and 4-CH_3_-C_6_H_4_ group (methyl as electron releasing group) in the two series. Whence, the derivatives bearing Ar = 4-Cl-C_6_H_4_ group (at position 3 in the two series) were slightly more active than those bearing Ar = 4-CH_3_-C_6_H_4_ group than those bearing Ar = C_6_H_5_ group.

In addition, we observed that, there was a ranking in the order of rings bearing halogen atoms (Cl, Br and F) in the series of **18a**–**u**, where, **18d** (IC_50_ = 77.1 ± 4.2 µM) >**18e** (IC_50_ = 81.2 ± 5.5 µM) >**18f** (IC_50_ = 92.8 ± 6.7 µM); **18k** (IC_50_ = 74.0 ± 3.1 µM) >**18l** (IC_50_ = 78.7 ± 5.1 µM) >**18m** (IC_50_ = 80.3 ± 3.9 µM) and **18**r (IC_50_ = 73.0 ± 1.9 µM) >**18**s (IC_50_ = 78.2 ± 3.2 µM) >**18t** (IC_50_ = 78.7 ± 4.7 µM). Therefore, the derivatives bearing Ar_2_ = 4-Cl-C_6_H_4_ group (at position 7) > Ar_2_ = 4-Br-C_6_H_4_ group > Ar_2_ = 4-F-C_6_H_4_ group.

Moreover, the derivatives bearing Ar_2_ = 4-CH_3_O-C_6_H_4_ group (at position 7) > Ar_2_ = 4-CH_3_-C_6_H_4_ group, where, **18c** (IC_50_ = 75.9 ± 5.3µM) >**18b** (IC_50_ = 90.9 ± 6.5 µM); **18j** (IC_50_ = 77.4 ± 2.9µM) >**18i** (IC_50_ = 83.3 ± 4.3 µM) and **18q** (IC_50_ = 72.8 ± 3.9 µM) >**18p** (IC_50_ = 87.8 ± 5.4 µM). Therefore, the replacement of the 4-CH_3_-C_6_H_4_ group by 4-CH_3_O-C_6_H_4_ group was impacted and increased the activity against liver cancer.

**Figure 4:**
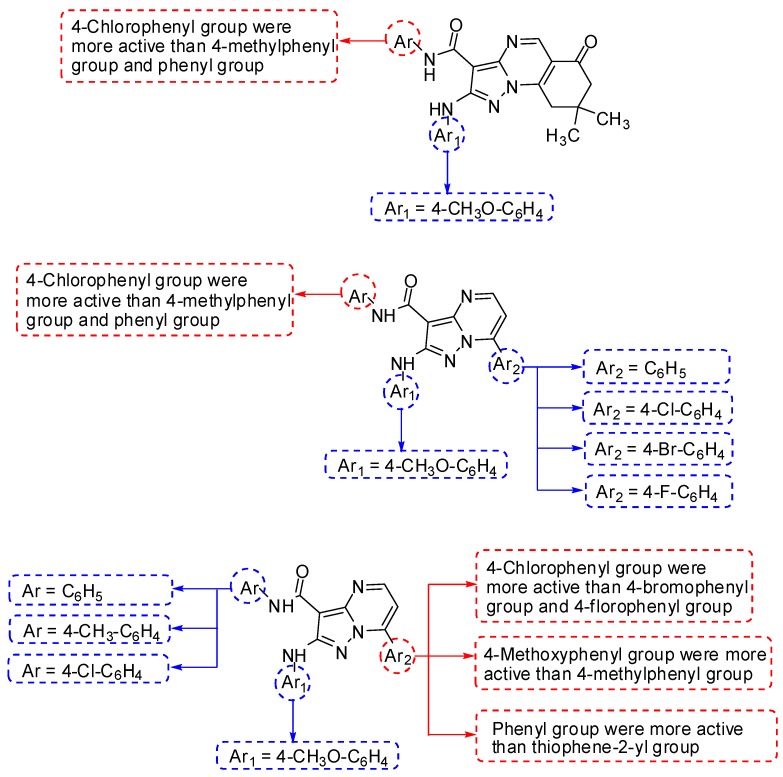
A brief Structure-activity relationship (SAR) study of **18a**–**u** and **25a**–**c** against liver (HepG2) cell lines.

Furthermore, we observed that, the derivatives bearing phenyl group (at position 7) more active than those bearing thiophen-2-yl group, where,**18a** (IC_50_ = 85.4 ± 5.1 µM) >**18g** (IC_50_ = 91.1 ± 6.4 µM); **18h** (IC_50_ = 73.2 ± 3.2 µM) >**18n** (IC_50_ = 82.5 ± 5.7 µM) and **18o** (IC_50_ = 72.2 ± 3.8 µM) >**18u** (IC_50_ = 83.1 ± 5.1 µM). Therefore, the introduction of thiophen-2-yl group in the series decreased the activity. A brief Structure-activity relationship (SAR) study has been presented in Figure 4.

### 2.4. Cell-Cycle Analysis and Apoptotic Changes

**Figure 5:**
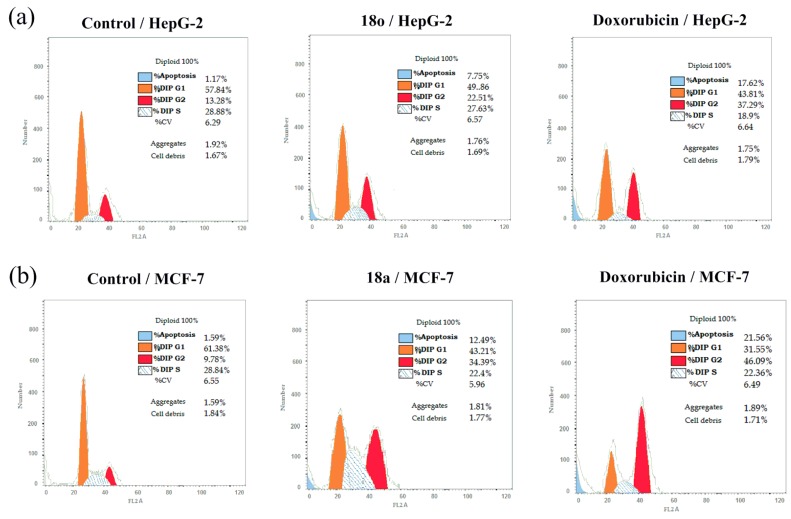
(**a**) Effect of compound **18o** on DNA-ploidy flow cytometric analysis of HepG-2 cancer cells, the cells were treated with DMSO as control and with doxorubicin as a positive control, for 24 h. (**b**) Effect of compound **18a** on DNA-ploidy flow cytometric analysis of MCF-7 cancer cells, the cells were treated with DMSO as control and with doxorubicin as a positive control, for 24 h.

Cell cycle can be defined as cell reproduction via replication of the DNA followed by division of the nucleus and partitioning of the cytoplasm to yield two daughter cells. This cell cycle comprises four different phases. G1 phase occurs between nuclear division (M phase) and DNA synthesis (S phase); G2 phase occurs between S phase and M phase. These gaps allow for the repair of DNA damage and replication errors [34]. According to the cytotoxicity screening in Table 1, and because most of the compounds did not show statistical significant differences compared to the positive control, two compounds (**18o** and **18a**) have been selected for further experiments. The effect of compounds **18o** and **18a** after 24 h of treatment by propidium iodide on cell cycle progression, using the flow cytometry (Figure 5a), was investigated against HepG-2 and MCF-7, respectively.

Compound **18o** induced significant alterations in the cell-cycle phases of HepG2 cells when compared to control. Interestingly, exposure of HepG2 cells to **18o** induced a significant increase in the percentage of cells at pre-G1 and G2/M phases by 6.6 folds and 1.7 folds, with a concurrent significant reduction in the percentage of cells at G0/G1 by 1.2 folds without any significant changes in S phase compared to control, respectively.

Moreover, treatment of MCF-7 cancer cells with compound **18a** caused a significant increase in pre-G1 and G2/M phases percent by 7.9 folds and 3.5 folds with a significant reduction in the percentage of cells at G0/G1 by 1.4 folds and slightly increase in S phases by 0.9 folds compared to control, respectively (Figure 5b). However, the positive control showed better results. In case of HepG-2 cancer cells, Doxorubicin-induced a significant increase in the percentage of cells at pre-G1 and G2/M phases by 2.2 folds and 1.6 folds, with a significant reduction in the percentage of cells at G0/G1 and S phases by 1.14 and 1.46 folds compared to compound **18o**. In addition, in case of MCF-7 cancer cells, Doxorubicin-induced a significant increase in the percentage of cells at pre-G1 and G2/M phases by 1.7-folds and 1.4 folds, with a significant reduction in the percentage of cells at G0/G1 and did not show any significant increase in S phases compared to compound **18o**. From these results, it can be concluded that compounds **18o** and **18a** inhibit the cell growth through cell cycle arrest at G2/M phase, which in turn induces cell death by apoptosis. These results are in agreement with the cytotoxicity screening results.

### 2.5. Annexin V-FITC Apoptosis Assay

**Figure 6:**
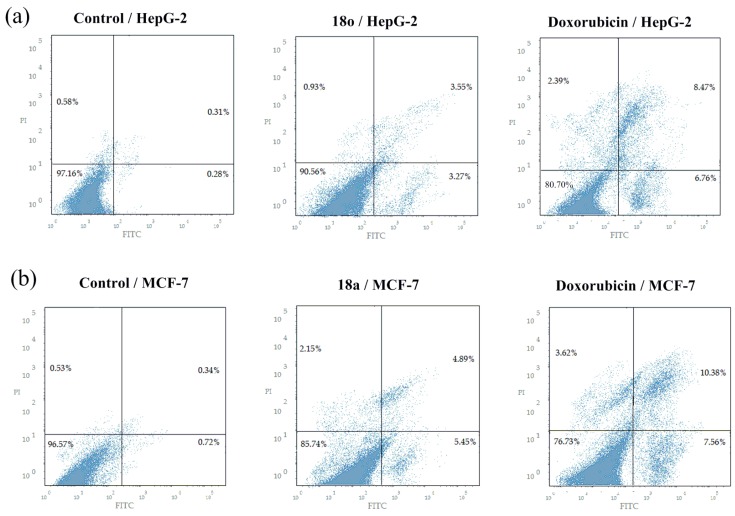
(**a**) Effect of compound **18o** on the percentage of annexin V-FITC positive staining in HepG-2 cancer cells, the cells were treated with DMSO as control and with doxorubicin as a positive control, for 24 h. (**b**) Effect of compound **18a** on the percentage of annexin V-FITC positive staining in MCF-7 cancer cells, the cells were treated with DMSO as control and with doxorubicin as a positive control, for 24 h.

The apoptotic effect of compounds **18o** and **18a** was carried out using Annexin V-FITC/PI (AV/PI) dual staining assay (Figure 6). The results revealed that HepG2 and MCF-7cells, treated with compounds **18o** and **18a**, respectively, showed a significant increase in the percent of annexin V-FITC positive apoptotic cells (UR & LR) by 11.6 folds and 9.8 folds compared to control, respectively. However, doxorubicin showed 2.2 folds and 1.7 folds increases in apoptotic cells % compared to compounds **18o** and **18a**, respectively. These results reveal that the cytotoxicity activities of compounds **18o** and **18a** are due to their potent pro-apoptotic activity.

## 3. Materials and Methods

### 3.1. Chemistry

All melting points were measured on a Gallenkamp melting point apparatus and are uncorrected. The IR spectra were recorded (KBr disk) on a 1650 FT-IR instrument (Perkin Elmer, Waltham, MA, USA). ^1^H-NMR (400 MHz) and ^13^C-NMR (100 MHz) spectra were recorded on a Varian spectrometer (Varian, Inc., Palo Alto, CA, USA) using DMSO-*d*_6_ or CDCl_3_ as solvent and TMS as an internal standard. Chemical shifts are reported in ppm. Coupling constants (*J*) are expressed in Hz. Mass spectra were recorded on a Varian MAT 112 spectrometer at 70 eV. Elemental analyses were performed at the Microanalytical Center, Cairo University, Egypt. The progress of the reactions was monitored by thin-layer chromatography (TLC) using aluminum sheets coated with silica gel F_254_ (Merck, Darmstadt, Germany), viewing under a short-wavelength UV lamp effected detection. All evaporations were carried out under reduced pressure at 40 °C.

*Synthesis of 5-amino-3-(arylamino)-1H-pyrazole-4-carboxamides***11a**–**c**. Compounds of this series were prepared according to the literature procedure.

*5-Amino-3-(4-methoxyphenylamino)-N-phenyl-1H-pyrazole-4-carboxamide* (**11a**). White crystals; m.p. 175–177 °C [29].

*5-Amino-3-(4-methoxyphenylamino)-N-(4-methylphenyl)-1H-pyrazole-4-carboxamide* (**11b**). White crystals; m.p. 198–200 °C [29].

*5-Amino-3-(4-methoxyphenylamino)-N-(4-chlorophenyl)-1H-pyrazole-4-carboxamide* (**11c**). White crystals; m.p. 190–192 °C [29].

*General Procedure for Synthesis of7-aryl-2-(arylamino)pyrazolo[1,5-a]pyrimidine-3-carboxamides***18a**–**u**. A mixture of compounds **11a**–**c** (0.01 mol) with enaminones **12a**–**g** {e.g., 3-(dimethylamino)-1-phenylprop-2-en-1-one (**12a**), 3-(dimethylamino)-1-(4-methylphenyl)prop-2-en-1-one (**12b**), 3-(dimethylamino)-1-(4-methoxyphenyl)prop-2-en-1-one (**12c**), 1-(4-chlorophenyl)-3-(dimethyl-amino)prop-2-en-1-one (**12d**), 1-(4-bromophenyl)-3-(dimethylamino)prop-2-en-1-one (**12e**), 3-(dimethylamino)-1-(4-fluorophenyl)prop-2-en-1-one (**12f**) or 3-(dimethylamino)-1-(thiophen-2-yl)prop-2-en-1-one (**12g**)} (0.01 mol) in glacial acetic acid (25 mL), the reaction mixture was refluxed for 1 h and then left to cool. The solid product was filtered off, washed with ethanol, dried and finally recrystallized from DMF/H_2_O to afford the corresponding pyrazolo[1,5-*a*]pyrimidine derivatives **18a**–**u**.

*2-(4-Methoxyphenylamino)-N,7-diphenylpyrazolo[1,5-a]pyrimidine-3-carboxamide* (**18a**). Yellow crystals, m.p. 218–220 °C, yield (72%). IR (KBr) *ν*_max_/cm^−1^ 3346 (NH), 1658 (C=O). ^1^H-NMR (CDCl_3_, 400 MHz, *δ* ppm): 3.80 (s, 3H, OCH_3_), 6.88 (d, 2H, *J* = 9.0 Hz,ArH), 6.96 (d, 1H, *J* = 4.8 Hz, pyrimidine), 7.12 (t, 1H, ArH), 7.36–7.42 (m, 5H, ArH), 7.62 (d, 2H, *J* = 9.0 Hz,ArH), 7.74 (d, 2H, *J* = 8.4 Hz,ArH), 8.11 (d, 2H, *J* = 8.3 Hz,ArH), 8.49 (d, 1H, *J* = 4.8 Hz, pyrimidine), 9.40 (s, 1H, NH), 10.05 (s, 1H, NH). ^13^C-NMR (CDCl_3_, 100 MHz, *δ* ppm): 55.7 (C, OCH_3_), 87.8 (C, C_3_-pyrazolopyrimidine), 107.0 (C, C_6_-pyrazolopyrimidine), 114.4, 119.2, 120.2, 123.7, 127.7, 129.1, 129.5, 129.6 (14C, Ar), 134.1 (C, C_3a_-pyrazolopyrimidine), 138.8, 142.4, 146.7 (3C, Ar), 147.9 (C, C_7_-pyrazolopyrimidine), 149.6 (C, Ar), 154.5 (C, C_2_-pyrazolopyrimidine), 157.8 (C, C_5_-pyrazolopyrimidine), 163.3 (C=O). MS (*m*/*z*, %): 435 (M^+^, 73.86). Anal. Calcd. (%) for C_26_H_21_N_5_O_2_ (435.48): C, 71.71; H, 4.86; N, 16.08. Found: C, 71.80; H, 4.81; N, 16.00%.

*2-(4-Methoxyphenylamino)-N-phenyl-7-(4-methylphenyl)-pyrazolo[1,5-a]pyrimidine-3-carboxamide* (**18b**). Yellow crystals, m.p. 219–221 °C, yield (77%). IR (KBr) *ν*_max_/cm^−1^ 3337 (NH), 1658 (C=O). ^1^H-NMR (CDCl_3_, 400 MHz, *δ* ppm): 2.49 (s, 3H, CH_3_), 3.80 (s, 3H, OCH_3_), 6.87 (d, 2H, *J* = 8.9 Hz, ArH), 6.91 (d, 1H, *J* = 4.7 Hz, pyrimidine), 7.12 (t, 1H, ArH), 7.36 (d, 2H, *J* = 8.3 Hz, ArH), 7.38 (t, 2H, ArH), 7.60 (d, 2H, *J* = 8.9 Hz, ArH), 7.73 (d, 2H, *J* = 7.6 Hz, ArH), 8.08 (d, 2H, *J* = 8.1 Hz,ArH), 8.43 (d, 1H, *J* = 4.7 Hz, pyrimidine), 9.38 (s, 1H, NH), 10.01 (s, 1H, NH). ^13^C-NMR (CDCl_3_, 100 MHz, *δ* ppm): 21.8 (C, CH_3_), 55.7 (C, OCH_3_), 87.7 (C, C_3_-pyrazolopyrimidine), 107.0 (C, C_6_-pyrazolopyrimidine), 114.4, 119.1, 120.1, 123.7, 127.6, 129.1, 129.4, 129.6 (14C, Ar), 134.1 (C, C_3a_-pyrazolopyrimidine), 138.8, 142.3, 146.6 (3C, Ar), 147.8 (C, C_7_-pyrazolopyrimidine), 149.6 (C, Ar), 154.4 (C, C_2_-pyrazolopyrimidine), 157.7 (C, C_5_-pyrazolopyrimidine), 163.3 (C=O). MS (*m*/*z*, %): 449 (M^+^, 67.43). Anal. Calcd. (%) for C_27_H_23_N_5_O_2_ (449.50): C, 72.14; H, 5.16; N, 15.58. Found: C, 72.10; H, 5.20; N, 15.60%.

*7-(4-Methoxyphenyl)-2-(4-methoxyphenylamino)-N-phenylpyrazolo[1,5-a]pyrimidine-3-carboxamide* (**18c**). Yellow crystals, m.p. 206–208 °C, yield (76%). IR (KBr) *ν*_max_/cm^−1^ 3340 (NH), 1646 (C=O). ^1^H-NMR (CDCl_3_, 400 MHz, *δ* ppm): 3.80 (s, 3H, OCH_3_), 3.91 (s, 3H, OCH_3_), 6.87 (d, 2H, *J* = 8.9 Hz, ArH), 6.89 (d, 1H, *J* = 4.8 Hz, pyrimidine), 7.05 (d, 2H, *J* = 8.8 Hz, ArH), 7.11 (t, 1H, ArH), 7.37 (t, 2H, ArH), 7.60 (d, 2H, *J* = 8.9 Hz, ArH), 7.72 (d, 2H, *J* = 7.6 Hz, ArH), 8.18 (d, 2H, *J* = 8.8 Hz, ArH), 8.40 (d, 1H, *J* = 4.8 Hz, pyrimidine), 9.36 (s, 1H, NH), 10.02 (s, 1H, NH). ^13^C-NMR (CDCl_3_, 100 MHz, *δ* ppm): 55.5 (C, OCH_3_), 55.6 (C, OCH_3_), 87.4 (C, C_3_-pyrazolopyrimidine), 106.4 (C, C_6_-pyrazolopyrimidine), 113.9, 114.2, 119.0, 120.0, 122.4, 123.5, 128.9, 131.3 (14C, Ar), 134.0 (C, C_3a_-pyrazolopyrimidine), 138.6, 146.0 (2C, Ar), 147.7 (C, C_7_-pyrazolopyrimidine), 149.3 (C, Ar), 154.3 (C, C_2_-pyrazolopyrimidine), 157.5 (C, C_5_-pyrazolopyrimidine), 162.2 (C, Ar), 163.2 (C=O). MS (*m*/*z*, %): 465 (M^+^, 69.48). Anal. Calcd. (%) for C_27_H_23_N_5_O_3_ (465.50): C, 69.66; H, 4.98; N, 15.04. Found: C, 69.70; H, 4.95; N, 15.00%.

*7-(4-Chlorophenyl)-2-(4-methoxyphenylamino)-N-phenylpyrazolo[1,5-a]pyrimidine-3-carboxamide* (**18d**). Yellow crystals, m.p. 252–253 °C, yield (72%). IR (KBr) *ν*_max_/cm^−1^ 3343 (NH), 1648 (C=O). ^1^H-NMR (CDCl_3_, 400 MHz, *δ* ppm): 3.81 (s, 3H, OCH_3_), 6.88 (d, 2H, *J* = 9.0 Hz, ArH), 6.94 (d, 1H, *J* = 4.7 Hz, pyrimidine), 7.13 (t, 1H, ArH), 7.39 (t, 2H, ArH), 7.58 (d, 4H, *J* = 8.8 Hz, ArH), 7.74 (d, 2H, *J* = 8.6 Hz, ArH), 8.15 (d, 2H, *J* = 8.7 Hz, ArH), 8.52 (d, 1H, *J* = 4.7 Hz, pyrimidine), 9.42 (s, 1H, NH), 9.99 (s, 1H, NH). ^13^C-NMR (CDCl_3_, 100 MHz, *δ* ppm): 55.7 (C, OCH_3_), 88.0 (C, C_3_-pyrazolopyrimidine), 107.0 (C, C_6_-pyrazolopyrimidine), 114.4, 119.2, 120.2, 123.8, 129.1, 129.1, 130.9, 131.8 (14C, Ar), 133.9 (C, C_3a_-pyrazolopyrimidine), 134.6, 138.0, 138.7 (3C, Ar), 145.3 (C, C_7_-pyrazolopyrimidine), 149.7 (C, Ar), 154.6 (C, C_2_-pyrazolopyrimidine), 157.9 (C, C_5_-pyrazolopyrimidine), 163.2 (C=O). MS (*m*/*z*, %): 469 (M^+^, 78.23). Anal. Calcd. (%) for C_26_H_20_ClN_5_O_2_ (469.92): C, 66.45; H, 4.29; N, 14.90. Found: C, 66.40; H, 4.30; N, 14.95%.

*7-(4-Bromophenyl)-2-(4-methoxyphenylamino)-N-phenylpyrazolo[1,5-a]pyrimidine-3-carboxamide* (**18e**). Yellow crystals, m.p. 278–280 °C, yield (69%). IR (KBr) *ν*_max_/cm^−1^ 3365 (NH), 1650 (C=O). ^1^H-NMR (DMSO-*d*_6_, 400 MHz, *δ* ppm): 3.73 (s, 3H, OCH_3_), 6.93 (d, 2H, *J* = 9.0 Hz, ArH), 7.12 (t, 1H, ArH), 7.39 (t, 2H, ArH), 7.41 (d, 1H, *J* = 4.8 Hz, pyrimidine), 7.59 (d, 2H, *J* = 9.0 Hz, ArH), 7.73 (d, 2H, *J* = 7.6 Hz, ArH), 7.90 (d, 2H, *J* = 8.7 Hz, ArH), 8.20 (d, 2H, *J* = 8.7 Hz, ArH), 8.74 (d, 1H, *J* = 4.8 Hz, pyrimidine), 9.26 (s, 1H, NH), 10.03 (s, 1H, NH). ^13^C-NMR (DMSO-*d*_6_, 100 MHz, *δ* ppm): 55.7 (C, OCH_3_), 87.6 (C, C_3_-pyrazolopyrimidine), 106.9 (C, C_6_-pyrazolopyrimidine), 114.4, 119.1, 120.5, 123.3, 129.4, 129.8, 131.0, 131.6 (14C, Ar), 133.7 (C, C_3a_-pyrazolopyrimidine), 133.4, 136.1, 138.7 (3C, Ar), 145.2 (C, C_7_-pyrazolopyrimidine), 149.5 (C, Ar), 154.8 (C, C_2_-pyrazolopyrimidine), 157.1 (C, C_5_-pyrazolopyrimidine), 163.7 (C=O). MS (*m*/*z*, %): 514 (M^+^, 81.26). Anal. Calcd. (%) for C_26_H_20_BrN_5_O_2_ (514.37): C, 60.71; H, 3.92; N, 13.62. Found: C, 60.65; H, 3.97; N, 13.65%.

*7-(4-Fluorophenyl)-2-(4-methoxyphenylamino)-N-phenylpyrazolo[1,5-a]pyrimidine-3-carboxamide* (**18f**). Yellow crystals, m.p. 237–239 °C, yield (70%). IR (KBr) *ν*_max_/cm^−1^ 3343 (NH), 1647 (C=O). ^1^H-NMR (DMSO-*d*_6_, 400 MHz, *δ* ppm): 3.73 (s, 3H, OCH_3_), 6.91 (d, 2H, *J* = 9.0 Hz, ArH), 7.11 (t, 1H, ArH), 7.37 (d, 1H, *J* = 4.9 Hz, pyrimidine), 7.39 (d, 2H, *J* = 7.6 Hz, ArH), 7.52 (t, 2H, ArH), 7.58 (d, 2H, *J* = 9.0 Hz, ArH), 7.71 (d, 2H, *J* = 8.6 Hz, ArH), 8.31 (d, 2H, *J* = 8.9 Hz, ArH), 8.71 (d, 1H, *J* = 4.8 Hz, pyrimidine), 9.23 (s, 1H, NH), 10.01 (s, 1H, NH). ^13^C-NMR (DMSO-*d*_6_, 100 MHz, *δ* ppm): 55.2 (C, OCH_3_), 86.7 (C, C_3_-pyrazolopyrimidine), 108.3 (C, C_6_-pyrazolopyrimidine), 114.3, 115.7, 115.9, 118.8, 119.4, 123.5, 126.4, 129.1 (14C, Ar), 132.4 (C, C_3a_-pyrazolopyrimidine), 133.3, 138.4 (2C, Ar), 145.0 (C, C_7_-pyrazolopyrimidine), 147.1 (C, Ar), 151.1 (C, C_2_-pyrazolopyrimidine), 154.1 (C, C_5_-pyrazolopyrimidine), 156.6 (C, Ar), 162.2 (C=O). MS (*m*/*z*, %): 453 (M^+^, 87.33). Anal. Calcd. (%) for C_26_H_20_FN_5_O_2_ (453.47): C, 68.86; H, 4.45; N, 15.44. Found: C, 68.95; H, 4.40; N, 15.50%.

*2-(4-Methoxyphenylamino)-N-phenyl-7-(thiophen-2-yl)pyrazolo[1,5-a]pyrimidine-3-carboxamide* (**18g**). Yellow crystals, m.p. 233–235 °C, yield (71%). IR (KBr) *ν*_max_/cm^−1^ 3356 (NH), 1652 (C=O). ^1^H-NMR (DMSO-*d*_6_, 400 MHz, *δ* ppm): 3.78 (s, 3H, OCH_3_), 7.03 (d, 2H, *J* = 8.4 Hz, ArH), 7.12 (t, 1H, ArH), 7.40 (t, 2H, ArH), 7.47 (t, 1H, *J* = 4.9 Hz, thiophene), 7.74 (d, 2H, *J* = 7.8 Hz, ArH), 7.84 (d, 2H, *J* = 8.5 Hz, ArH), 7.90 (d, 1H, *J* = 4.6 Hz, pyrimidine), 8.28 (d, 1H, *J* = 4.4 Hz, thiophene), 8.58 (d, 1H, *J* = 2.8 Hz, thiophene), 8.71 (d, 1H, *J* = 4.4 Hz, pyrimidine), 9.44 (s, 1H, NH), 10.07 (s, 1H, NH). ^13^C-NMR (DMSO-*d*_6_, 100 MHz, *δ* ppm): 55.7 (C, OCH_3_), 86.8 (C, C_3_-pyrazolopyrimidine), 107.3 (C, C_6_-pyrazolopyrimidine), 114.8, 119.2, 120.8, 126.3 (7C, Ar), 128.1, 129.8 (2C, thiophene), 130.1 (2C, Ar), 133.2 (C, thiophene), 133.9 (C, C_3a_-pyrazolopyrimidine), 134.5, 137.1 (2C, Ar), 139.4 (C, thiophene), 147.3 (C, Ar), 150.8 (C, C_2_-pyrazolopyrimidine), 154.4 (C, C_5_-pyrazolopyrimidine), 156.3 (C, C_7_-pyrazolopyrimidine), 162.9 (C=O). MS (*m*/*z*, %): 441 (M^+^, 100). Anal. Calcd. (%) for C_24_H_19_N_5_O_2_S (441.50): C, 65.29; H, 4.34; N, 15.86. Found: C, 65.35; H, 4.30; N, 15.90%.

*2-(4-Methoxyphenylamino)-7-phenyl-N-(4-methylphenyl)-pyrazolo[1,5-a]pyrimidine-3-carboxamide* (**18h**). Yellow crystals, m.p. 251–253 °C, yield (76%). IR (KBr) *ν*_max_/cm^−1^ 3374 (NH), 1660 (C=O). ^1^H-NMR (CDCl_3_, 400 MHz, *δ* ppm): 2.35 (s, 3H, CH_3_), 3.80 (s, 3H, OCH_3_), 6.88 (d, 2H, *J* = 9.0 Hz, ArH), 6.95 (d, 1H, *J* = 4.8 Hz, pyrimidine), 7.18 (d, 2H, *J* = 8.2 Hz, ArH), 7.40 (d, 2H, *J* = 8.2 Hz, ArH), 7.60–7.64 (m, 5H, ArH), 8.11 (d, 2H, *J* = 8.2 Hz, ArH), 8.48 (d, 1H, *J* = 4.8 Hz, pyrimidine), 9.42 (s, 1H, NH), 9.97 (s, 1H, NH).^13^C-NMR (CDCl_3_, 100 MHz, *δ* ppm): 21.0 (C, CH_3_), 55.7 (C, OCH_3_), 87.8 (C, C_3_-pyrazolopyrimidine), 106.9 (C, C_6_-pyrazolopyrimidine), 114.4, 119.1, 120.2, 127.7, 129.4, 129.6, 133.3 (14C, Ar), 134.2 (C, C_3a_-pyrazolopyrimidine), 136.2, 142.4, 146.6 (3C, Ar), 147.8 (C, C_7_-pyrazolopyrimidine), 149.6 (C, Ar), 154.4 (C, C_2_-pyrazolopyrimidine), 157.8 (C, C_5_-pyrazolo-pyrimidine), 163.2 (C=O). MS (*m*/*z*, %): 449 (M^+^, 92.11). Anal. Calcd. (%) for C_27_H_23_N_5_O_2_ (449.50): C, 72.14; H, 5.16; N, 15.58. Found: C, 72.20; H, 5.11; N, 15.50%.

*2-(4-Methoxyphenylamino)-N,7-di-(4-methylphenyl)pyrazolo[1,5-a]pyrimidine-3-carboxamide* (**18i**). Yellow crystals, m.p. 261 °C, yield (74%). IR (KBr) *ν*_max_/cm^−1^ 3293 (NH), 1642 (C=O). ^1^H-NMR (CDCl_3_, 400 MHz, *δ* ppm): 2.35 (s, 3H, CH_3_), 2.49 (s, 3H, CH_3_), 3.80 (s, 3H, OCH_3_), 6.87 (d, 2H, *J* = 9.0 Hz, ArH), 6.91 (d, 1H, *J* = 4.8 Hz, pyrimidine), 7.18 (d, 2H, *J* = 8.2 Hz, ArH), 7.37 (d, 2H, *J* = 8.0 Hz, ArH), 7.60 (d, 2H, *J* = 9.0 Hz, ArH), 7.61 (d, 2H, *J* = 8.5 Hz, ArH), 8.08 (d, 2H, *J* = 8.3 Hz, ArH), 8.43 (d, 1H, *J* = 4.8 Hz, pyrimidine), 9.40 (s, 1H, NH), 9.94 (s, 1H, NH). ^13^C-NMR (CDCl_3_, 100 MHz, *δ* ppm): 21.0 (C, CH_3_), 21.8 (C, CH_3_), 55.7 (C, OCH_3_), 87.7 (C, C_3_-pyrazolopyrimidine), 106.9 (C, C_6_-pyrazolopyrimidine), 114.4, 119.1, 120.1, 127.6, 129.4, 129.5, 129.6, 133.2 (14C, Ar), 134.1 (C, C_3a_-pyrazolopyrimidine), 136.2, 142.3, 146.5 (3C, Ar), 147.7 (C, C_7_-pyrazolopyrimidine), 149.5 (C, Ar), 154.4 (C, C_2_-pyrazolopyrimidine), 157.7 (C, C_5_-pyrazolopyrimidine), 163.2 (C=O). MS (*m*/*z*, %): 463 (M^+^, 100). Anal. Calcd. (%) for C_28_H_25_N_5_O_2_ (463.53): C, 72.55; H, 5.44; N, 15.11. Found: C, 72.55; H, 5.44; N, 15.11%.

*7-(4-Methoxyphenyl)-2-(4-methoxyphenylamino)-N-(4-methylphenyl)pyrazolo[1,5-a]pyrimidine-3-carboxamide* (**18j**). Yellow crystals, m.p. 244–245 °C, yield (75%). IR (KBr) *ν*_max_/cm^−1^ 3368 (NH), 1649 (C=O). ^1^H-NMR (CDCl_3_, 400 MHz, *δ* ppm): 2.35 (s, 3H, CH_3_), 3.80 (s, 3H, OCH_3_), 3.94 (s, 3H, OCH_3_), 6.88 (d, 2H, *J* = 9.0 Hz, ArH), 6.93 (d, 1H, *J* = 4.8 Hz, pyrimidine), 7.10 (d, 2H, *J* = 9.0 Hz, ArH), 7.18 (d, 2H, *J* = 8.2 Hz, ArH), 7.61–7.64 (m, 4H, ArH), 8.22 (d, 2H, *J* = 8.9 Hz, ArH), 8.45 (d, 1H, *J* = 4.8 Hz, pyrimidine), 9.42 (s, 1H, NH), 9.98 (s, 1H, NH). ^13^C-NMR (CDCl_3_, 100 MHz, *δ* ppm): 21.0 (C, CH_3_), 55.7 (C, OCH_3_), 55.7 (C, OCH_3_), 87.7 (C, C_3_-pyrazolopyrimidine), 106.5 (C, C_6_-pyrazolopyrimidine), 114.1, 114.4, 119.1, 120.2, 122.7, 129.6, 131.5, 133.2 (14C, Ar), 134.2 (C, C_3a_-pyrazolopyrimidine), 136.2, 146.2 (2C, Ar), 147.9 (C, C_7_-pyrazolopyrimidine), 149.5 (C, Ar), 154.4 (C, C_2_-pyrazolopyrimidine), 157.8 (C, C_5_-pyrazolopyrimidine), 162.3 (C, Ar), 163.3 (C=O). MS (*m*/*z*, %): 479 (M^+^, 92.77). Anal. Calcd. (%) for C_28_H_25_N_5_O_3_ (479.53): C, 70.13; H, 5.25; N, 14.60. Found: C, 70.05; H, 5.30; N, 14.55%.

*7-(4-Chlorophenyl)-2-(4-methoxyphenylamino)-N-(4-methylphenyl)pyrazolo[1,5-a]pyrimidine-3-carboxamide* (**18k**). Yellow crystals, m.p. 267–269 °C, yield (71%). IR (KBr) *ν*_max_/cm^−1^ 3315 (NH), 1662 (C=O). ^1^H-NMR (CDCl_3_, 400 MHz, *δ* ppm): 2.35 (s, 3H, CH_3_), 3.81 (s, 3H, OCH_3_), 6.88 (d, 2H, *J* = 9.0 Hz, ArH), 6.93 (d, 1H, *J* = 4.7 Hz, pyrimidine), 7.19 (d, 2H, *J* = 8.2 Hz, ArH), 7.57–7.62 (m, 6H, ArH), 8.14 (d, 2H, *J* = 8.7 Hz, ArH), 8.50 (d, 1H, *J* = 4.7 Hz, pyrimidine), 9.43 (s, 1H, NH), 9.91 (s, 1H, NH). ^13^C-NMR (CDCl_3_, 100 MHz, *δ* ppm): 21.0 (C, CH_3_), 55.7 (C, OCH_3_), 88.0 (C, C_3_-pyrazolopyrimidine), 107.0 (C, C_6_-pyrazolopyrimidine), 114.4, 119.2, 120.2, 129.1, 129.6, 130.9, 131.7, 133.4 (14C, Ar), 134.1 (C, C_3a_-pyrazolopyrimidine), 134.4, 136.0, 137.9 (3C, Ar), 146.1 (C, C_7_-pyrazolopyrimidine), 149.7 (C, Ar), 154.6 (C, C_2_-pyrazolopyrimidine), 159.4 (C, C_5_-pyrazolopyrimidine), 163.1 (C=O). MS (*m*/*z*, %): 483 (M^+^, 87.08). Anal. Calcd. (%) for C_27_H_22_ClN_5_O_2_ (483.95): C, 67.01; H, 4.58; N, 14.47. Found: C, 67.10; H, 4.50; N, 14.50%.

*7-(4-Bromophenyl)-2-(4-methoxyphenylamino)-N-(4-methylphenyl)pyrazolo[1,5-a]pyrimidine-3-carboxamide* (**18l**). Yellow crystals, m.p. 278–279 °C, yield (68%). IR (KBr) *ν*_max_/cm^−1^ 3325 (NH), 1649 (C=O). ^1^H-NMR (DMSO-*d*_6_, 400 MHz, *δ* ppm): 2.30 (s, 3H, CH_3_), 3.74 (s, 3H, OCH_3_), 6.95 (d, 2H, *J* = 9.3 Hz, ArH), 7.20 (d, 2H, *J* = 8.6 Hz, ArH), 7.44 (d, 1H, *J* = 4.1 Hz, pyrimidine), 7.62 (d, 2H, *J* = 8.4 Hz, ArH), 7.63 (d, 2H, *J* = 7.8 Hz, ArH), 7.93 (d, 2H, *J* = 8.3 Hz, ArH), 8.22 (d, 2H, *J* = 8.3 Hz, ArH), 8.77 (d, 1H, *J* = 4.3 Hz, pyrimidine), 9.31 (s, 1H, NH), 9.98 (s, 1H, NH). ^13^C-NMR (DMSO-*d*_6_, 100 MHz, *δ* ppm): 21.0 (C, CH_3_), 55.7 (C, OCH_3_), 88.0 (C, C_3_-pyrazolopyrimidine), 107.0 (C, C_6_-pyrazolopyrimidine), 114.9, 119.2, 120.5, 125.2, 129.7, 129.4, 131.0, 133.5 (14C, Ar), 134.2 (C, C_3a_-pyrazolopyrimidine), 134.4, 135.9, 137.9 (3C, Ar), 146.0 (C, C_7_-pyrazolopyrimidine), 149.7 (C, Ar), 154.5 (C, C_2_-pyrazolopyrimidine), 159.3 (C, C_5_-pyrazolopyrimidine), 163.1 (C=O). MS (*m*/*z*, %): 528 (M^+^, 26.25). Anal. Calcd. (%) for C_27_H_22_BrN_5_O_2_ (528.40): C, 61.37; H, 4.20; N, 13.25. Found: C, 61.45; H, 4.16; N, 13.30%.

*7-(4-Fluorophenyl)-2-(4-methoxyphenylamino)-N-(4-methylphenyl)pyrazolo[1,5-a]pyrimidine-3-carboxamide* (**18m**). Yellow crystals, m.p. 255–257 °C, yield (69%). IR (KBr) *ν*_max_/cm^−1^ 3329 (NH), 1652 (C=O). ^1^H-NMR (DMSO-*d*_6_, 400 MHz, *δ* ppm): 2.30 (s, 3H, CH_3_), 3.73 (s, 3H, OCH_3_), 6.93 (d, 2H, *J* = 9.0 Hz, ArH), 7.19 (d, 2H, *J* = 8.3 Hz, ArH), 7.39 (d, 1H, *J* = 4.8 Hz, pyrimidine), 7.54 (d, 2H, *J* = 8.9 Hz, ArH), 7.60 (d, 2H, *J* = 9.0 Hz, ArH), 7.62 (d, 2H, *J* = 8.4 Hz, ArH), 8.32 (d, 2H, *J* = 9.0 Hz, ArH), 8.74 (d, 1H, *J* = 4.8 Hz, pyrimidine), 9.27 (s, 1H, NH), 9.97 (s, 1H, NH). ^13^C-NMR (DMSO-*d*_6_, 100 MHz, *δ* ppm): 21.0 (C, CH_3_), 55.8 (C, OCH_3_), 87.9 (C, C_3_-pyrazolopyrimidine), 107.0 (C, C_6_-pyrazolopyrimidine), 114.6, 116.1, 119.5, 120.5, 129.5, 130.9, 131.7 (13C, Ar), 134.2 (C, C_3a_-pyrazolopyrimidine), 134.2, 136.1, 138.0 (3C, Ar), 146.2 (C, C_7_-pyrazolopyrimidine), 149.6 (C, Ar), 154.3 (C, C_2_-pyrazolopyrimidine), 159.0 (C, C_5_-pyrazolopyrimidine), 160.1 (C, Ar), 162.9 (C=O). MS (*m/z*, %): 467 (M^+^, 45.13). Anal. Calcd. (%) for C_27_H_22_FN_5_O_2_ (467.49): C, 69.37; H, 4.74; N, 14.98. Found: C, 69.30; H, 4.80; N, 15.05%.

*2-(4-Methoxyphenylamino)-7-(thiophen-2-yl)-N-(4-methylphenyl)pyrazolo[1,5-a]pyrimidine-3-carboxamide* (**18n**). Yellow crystals, m.p. 278–279 °C, yield (70%). IR (KBr) *ν*_max_/cm^−1^ 3345 (NH), 1652 (C=O). ^1^H-NMR (DMSO-*d*_6_, 400 MHz, *δ* ppm): 2.30 (s, 3H, CH_3_), 3.78 (s, 3H, OCH_3_), 7.03 (d, 2H, *J* = 8.9 Hz, ArH), 7.20 (d, 2H, *J* = 8.1 Hz, ArH), 7.47 (t, 1H, thiophene), 7.62 (d, 2H, *J* = 8.0 Hz, ArH), 7.83 (d, 2H, *J* = 8.6 Hz, ArH), 7.90 (d, 1H, *J* = 3.6 Hz, pyrimidine), 8.28 (d, 1H, *J* = 4.7 Hz, thiophene), 8.59 (d, 1H, *J* = 2.3 Hz, thiophene), 8.70 (d, 1H, *J* = 4.8 Hz, pyrimidine), 9.46 (s, 1H, NH), 10.00 (s, 1H, NH). ^13^C-NMR (DMSO-*d*_6_, 100 MHz, *δ* ppm): 21.0 (C, CH_3_), 55.7 (C, OCH_3_), 87.5 (C, C_3_-pyrazolopyrimidine), 107.1 (C, C_6_-pyrazolopyrimidine), 114.1, 119.5, 120.3 (6C, Ar), 127.6, 128.1, 129.8 (3C, thiophene), 130.0 (2C, Ar), 133.6 (C, C_3a_-pyrazolopyrimidine), 133.5, 134.5, 137.1 (3C, Ar), 139.8 (C, thiophene), 147.8 (C, Ar), 151.5 (C, C_2_-pyrazolopyrimidine), 154.1 (C, C_5_-pyrazolopyrimidine), 157.2 (C, C_7_-pyrazolopyrimidine), 163.0 (C=O). MS (*m*/*z*, %): 455 (M^+^, 65.71). Anal. Calcd. (%) for C_25_H_21_N_5_O_2_S (455.53): C, 65.92; H, 4.65; N, 15.37. Found: C, 66.00; H, 4.60; N, 15.31%.

*N-(4-Chlorophenyl)-2-(4-methoxyphenylamino)-7-phenylpyrazolo[1,5-a]pyrimidine-3-carboxamide* (**18o**). Yellow crystals, m.p. 252–254 °C, yield (73%). IR (KBr) *ν*_max_/cm^−1^ 3336 (NH), 1650 (C=O). ^1^H-NMR (DMSO-*d*_6_, 400 MHz, *δ* ppm): 3.72 (s, 3H, OCH_3_), 6.90 (d, 2H, *J* = 9.0 Hz, ArH), 7.40 (d, 1H, *J* = 4.8 Hz, pyrimidine), 7.44 (d, 2H, *J* = 8.8 Hz, ArH), 7.61 (d, 2H, *J* = 9.0 Hz, ArH), 7.68–7.70 (m, 3H, ArH), 7.78 (d, 2H, *J* = 8.9 Hz, ArH), 8.23 (d, 2H, *J* = 7.2 Hz, ArH), 8.75 (d, 1H, *J* = 4.8 Hz, pyrimidine), 9.20 (s, 1H, NH), 10.11 (s, 1H, NH). ^13^C-NMR (DMSO-*d*_6_, 100 MHz, *δ* ppm): 55.7 (C, OCH_3_), 87.9 (C, C_3_-pyrazolopyrimidine), 107.0 (C, C_6_-pyrazolopyrimidine), 114.5, 119.1, 120.2, 124.0, 128.4, 129.1, 130.9, 131.8 (13C, Ar), 134.0 (C, C_3a_-pyrazolopyrimidine), 134.6, 135.9, 138.0, 138.7 (4C, Ar), 145.6 (C, C_7_-pyrazolopyrimidine), 149.7 (C, Ar), 154.8 (C, C_2_-pyrazolopyrimidine), 158.0 (C, C_5_-pyrazolopyrimidine), 163.8 (C=O). MS (*m*/*z*, %): 469 (M^+^, 29.83). Anal. Calcd. (%) for C_26_H_20_ClN_5_O_2_ (469.92): C, 66.45; H, 4.29; N, 14.90. Found: C, 66.40; H, 4.35; N, 14.85%.

*N-(4-Chlorophenyl)-2-(4-methoxyphenylamino)-7-(4-methylphenyl)pyrazolo[1,5-a]pyrimidine-3-carboxamide* (**18p**). Yellow crystals, m.p. 261 °C, yield (75%). IR (KBr) *ν*_max_/cm^−1^ 3322 (NH), 1658 (C=O). ^1^H-NMR (DMSO-*d*_6_, 400 MHz, *δ* ppm): 2.32 (s, 3H, CH_3_), 3.74 (s, 3H, OCH_3_), 6.93 (d, 2H, *J* = 7.6 Hz, ArH), 7.42 (d, 1H, *J* = 4.8 Hz, pyrimidine), 7.45 (d, 2H, *J* = 7.7 Hz, ArH), 7.51 (d, 2H, *J* = 8.6 Hz, ArH), 7.64 (d, 2H, *J* = 7.8 Hz, ArH), 7.79 (d, 2H, *J* = 8.4 Hz, ArH), 8.19 (d, 2H, *J* = 7.5 Hz, ArH), 8.74 (d, 1H, *J* = 4.8 Hz, pyrimidine), 9.23 (s, 1H, NH), 10.14 (s, 1H, NH). ^13^C-NMR (DMSO-*d*_6_, 100 MHz, *δ* ppm): 21.0 (C, CH_3_), 55.7 (C, OCH_3_), 87.5 (C, C_3_-pyrazolopyrimidine), 107.0 (C, C_6_-pyrazolopyrimidine), 114.4, 119.3, 120.9, 129.0, 129.6, 131.0, 131.7, 133.4 (14C, Ar), 134.0 (C, C_3a_-pyrazolopyrimidine), 134.3, 136.0, 137.9 (3C, Ar), 146.1 (C, C_7_-pyrazolopyrimidine), 149.7 (C, Ar), 154.5 (C, C_2_-pyrazolopyrimidine), 159.4 (C, C_5_-pyrazolopyrimidine), 163.1 (C=O). MS (*m*/*z*, %): 483 (M^+^, 22.71). Anal. Calcd. (%) for C_27_H_22_ClN_5_O_2_ (483.95): C, 67.01; H, 4.58; N, 14.47. Found: C, 67.10; H, 4.50; N, 14.55%.

*N-(4-Chlorophenyl)-7-(4-methoxyphenyl)-2-(4-methoxyphenylamino)pyrazolo[1,5-a]pyrimidine-3-carboxamide* (**18q**). Yellow crystals, m.p. 266–267 °C, yield (74%). IR (KBr) *ν*_max_/cm^−1^ 3365 (NH), 1661 (C=O). ^1^H-NMR (DMSO-*d*_6_, 400 MHz, *δ* ppm): 3.74 (s, 3H, OCH_3_), 3.92 (s, 3H, OCH_3_), 6.95 (d, 2H, *J* = 8.8 Hz, ArH), 7.24 (d, 2H, *J* = 8.7 Hz, ArH), 7.40 (d, 1H, *J* = 4.7 Hz, pyrimidine), 7.44 (d, 2H, *J* = 8.7 Hz, ArH), 7.65 (d, 2H, *J* = 8.8 Hz, ArH), 7.78 (d, 2H, *J* = 8.6 Hz, ArH), 8.31 (d, 2H, *J* = 8.6 Hz, ArH), 8.70 (d, 1H, *J* = 4.7 Hz, pyrimidine), 9.22 (s, 1H, NH), 10.15 (s, 1H, NH). ^13^C-NMR (DMSO-*d*_6_, 100 MHz, *δ* ppm): 55.6 (C, OCH_3_), 55.7 (C, OCH_3_), 87.8 (C, C_3_-pyrazolopyrimidine), 106.4 (C, C_6_-pyrazolopyrimidine), 114.2, 114.6, 119.0, 120.5, 122.8, 129.5, 131.6 (13C, Ar), 133.1 (C, C_3a_-pyrazolopyrimidine), 134.8, 135.0, 136.1 (3C, Ar), 147.9 (C, C_7_-pyrazolopyrimidine), 149.4 (C, Ar), 154.5 (C, C_2_-pyrazolopyrimidine), 157.8 (C, C_5_-pyrazolopyrimidine), 161.5 (C, Ar), 163.2 (C=O). MS (*m*/*z*, %): 499 (M^+^, 18.46). Anal. Calcd. (%) for C_27_H_22_ClN_5_O_3_ (499.95): C, 64.86; H, 4.44; N, 14.01. Found: C, 64.95; H, 4.40; N, 14.05%.

*N,7-bis(4-Chlorophenyl)-2-(4-methoxyphenylamino)pyrazolo[1,5-a]pyrimidine-3-carboxamide* (**18r**). Yellow crystals, m.p. 282–284 °C, yield (70%). IR (KBr) *ν*_max_/cm^−1^ 3317 (NH), 1653 (C=O). ^1^H-NMR (DMSO-*d*_6_, 400 MHz, *δ* ppm): 3.74 (s, 3H, OCH_3_), 6.94 (d, 2H, *J* = 8.8 Hz, ArH), 7.44 (d, 2H, *J* = 8.6 Hz, ArH), 7.45 (d, 1H, *J* = 3.8 Hz, pyrimidine), 7.61 (d, 2H, *J* = 8.8 Hz, ArH), 7.78 (d, 4H, *J* = 8.4 Hz, ArH), 8.29 (d, 2H, *J* = 8.6 Hz, ArH), 8.76 (d, 1H, *J* = 4.7 Hz, pyrimidine), 9.23 (s, 1H, NH), 10.10 (s, 1H, NH). ^13^C-NMR (DMSO-*d*_6_, 100 MHz, *δ* ppm): 55.8 (C, OCH_3_), 87.4 (C, C_3_-pyrazolopyrimidine), 106.9 (C, C_6_-pyrazolopyrimidine), 115.0, 119.3, 120.4, 129.1, 129.7, 129.9, 131.6 (13C, Ar), 133.1 (C, C_3a_-pyrazolopyrimidine), 133.8, 134.3, 136.0, 137.9 (4C, Ar), 146.0 (C, C_7_-pyrazolopyrimidine), 149.8 (C, Ar), 154.5 (C, C_2_-pyrazolopyrimidine), 159.4 (C, C_5_-pyrazolopyrimidine), 162.9 (C=O). MS (*m/z*, %): 504 (M^+^, 22.87). Anal. Calcd. (%) for C_26_H_19_C_l2_N_5_O_2_ (504.37): C, 61.91; H, 3.80; N, 13.89. Found: C, 62.00; H, 3.75; N, 13.80%.

*7-(4-Bromophenyl)-N-(4-chlorophenyl)-2-(4-methoxyphenylamino)pyrazolo[1,5-a]pyrimidine-3-carboxamide* (**18s**). Yellow crystals, m.p. 275–277 °C, yield (67%). IR (KBr) *ν*_max_/cm^−1^ 3327 (NH), 1648 (C=O). ^1^H-NMR (DMSO-*d*_6_, 400 MHz, *δ* ppm): 3.74 (s, 3H, OCH_3_), 6.94 (d, 2H, *J* = 7.3 Hz, ArH), 7.44 (d, 2H, *J* = 7.2 Hz, ArH), 7.45 (d, 1H, *J* = 4.4 Hz, pyrimidine), 7.61 (d, 2H, *J* = 7.1 Hz, ArH), 7.79 (d, 2H, *J* = 7.9 Hz, ArH), 7.92 (d, 2H, *J* = 7.3 Hz, ArH), 8.21 (d, 2H, *J* = 7.8 Hz, ArH), 8.76 (d, 1H, *J* = 4.3 Hz, pyrimidine), 9.23 (s, 1H, NH), 10.11 (s, 1H, NH). ^13^C-NMR (DMSO-*d*_6_, 100 MHz, *δ* ppm): 55.7 (C, OCH_3_), 87.6 (C, C_3_-pyrazolopyrimidine), 106.9 (C, C_6_-pyrazolopyrimidine), 114.4, 119.2, 120.2, 123.2, 129.1, 129.6, 130.7, 131.4 (14C, Ar), 132.7 (C, C_3a_-pyrazolopyrimidine), 134.2, 136.0, 137.5 (3C, Ar), 146.0 (C, C_7_-pyrazolopyrimidine), 149.8 (C, Ar), 154.5 (C, C_2_-pyrazolopyrimidine), 159.5 (C, C_5_-pyrazolopyrimidine), 163.2 (C=O). MS (*m*/*z*, %): 548 (M^+^, 20.55). Anal. Calcd. (%) for C_26_H_19_BrClN_5_O_2_ (548.82): C, 56.90; H, 3.49; N, 12.76. Found: C, 57.00; H, 3.40; N, 12.80%.

*N-(4-Chlorophenyl)-7-(4-fluorophenyl)-2-(4-methoxyphenylamino)pyrazolo[1,5-a]pyrimidine-3-carboxamide* (**18t**). Yellow crystals, m.p. 251–252 °C, yield (67%). IR (KBr) *ν*_max_/cm^−1^ 3339 (NH), 1651 (C=O). ^1^H-NMR (DMSO-*d*_6_, 400 MHz, *δ* ppm): 3.74 (s, 3H, OCH_3_), 6.93 (d, 2H, *J* = 7.8 Hz, ArH), 7.44 (d, 2H, *J* = 8.7 Hz, ArH), 7.45 (d, 1H, *J* = 4.2 Hz, pyrimidine), 7.56 (d, 2H, *J* = 8.0 Hz, ArH), 7.62 (d, 2H, *J* = 8.0 Hz, ArH), 7.78 (d, 2H, *J* = 7.4 Hz, ArH), 8.33 (d, 2H, *J* = 8.1 Hz, ArH), 8.75 (d, 1H, *J* = 4.5 Hz, pyrimidine), 9.21 (s, 1H, NH), 10.10 (s, 1H, NH). ^13^C-NMR (DMSO-*d*_6_, 100 MHz, *δ* ppm): 55.8 (C, OCH_3_), 86.7 (C, C_3_-pyrazolopyrimidine), 108.3 (C, C_6_-pyrazolopyrimidine), 114.3, 115.9, 119.0, 119.4, 124.6, 128.1, 129.1 (13C, Ar), 132.3 (C, C_3a_-pyrazolopyrimidine), 133.2, 134.3, 138.4 (3C, Ar), 145.0 (C, C_7_-pyrazolopyrimidine), 147.3 (C, Ar), 151.1 (C, C_2_-pyrazolopyrimidine), 154.0 (C, C_5_-pyrazolopyrimidine), 156.6 (C, Ar), 162.3 (C=O). MS (*m*/*z*, %): 487 (M^+^, 21.30). Anal. Calcd. (%) for C_26_H_19_ClFN_5_O_2_ (487.91): C, 64.00; H, 3.93; N, 14.35. Found: C, 64.10; H, 4.00; N, 14.30%.

*N-(4-Chlorophenyl)-2-(4-methoxyphenylamino)-7-(thiophen-2-yl)pyrazolo[1,5-a]pyrimidine-3-carboxamide* (**18u**). Yellow crystals, m.p. 289–291 °C, yield (71%). IR (KBr) *ν*_max_/cm^−1^ 3293 (NH), 1644 (C=O). ^1^H-NMR (DMSO-*d*_6_, 400 MHz, *δ* ppm): 3.78 (s, 3H, OCH_3_), 7.04 (d, 2H, *J* = 9.0 Hz, ArH), 7.33 (d, 2H, *J* = 9.2 Hz, ArH), 7.47 (t, 1H, thiophene), 7.79 (d, 2H, *J* = 9.1 Hz, ArH), 7.84 (d, 2H, *J* = 8.9 Hz, ArH), 7.91 (d, 1H, *J* = 4.8 Hz, pyrimidine), 8.29 (d, 1H, *J* = 5.1 Hz, thiophene), 8.60 (d, 1H, *J* = 2.9 Hz, thiophene), 8.71 (d, 1H, *J* = 5.7 Hz, pyrimidine), 9.39 (s, 1H, NH), 10.14 (s, 1H, NH). ^13^C-NMR (DMSO-*d*_6_, 100 MHz, *δ* ppm): 55.7 (C, OCH_3_), 87.8 (C, C_3_-pyrazolopyrimidine), 107.1 (C, C_6_-pyrazolopyrimidine), 114.0, 119.5, 120.4, (6C, Ar), 127.6, 128.2, 129.7 (3C, thiophene), 130.0 (2C, Ar), 133.1 (C, C_3a_-pyrazolopyrimidine), 133.6, 134.5, 136.9 (3C, Ar), 139.7 (C, thiophene), 148.0 (C, Ar), 151.4 (C, C_2_-pyrazolopyrimidine), 154.2 (C, C_5_-pyrazolopyrimidine), 157.2 (C, C_7_-pyrazolopyrimidine), 162.9 (C=O). MS (*m*/*z*, %): 475 (M^+^, 74.59). Anal. Calcd. (%) for C_24_H_18_ClN_5_O_2_S (475.95): C, 60.56; H, 3.81; N, 14.71. Found: C, 60.50; H, 3.90; N, 14.80%.

*General Procedure for Synthesis of N-aryl-2-(arylamino)-8,8-dimethyl-6-oxo-6,7,8,9-tetrahydropyrazolo[1,5-a]quinazoline-3-carboxamides***25a**–**c**. A mixture of compounds **11a**–**c** (0.01 mol) with 2-((dimethyl-amino)methylene)-5,5-dimethylcyclohexane-1,3-dione (**19**, 0.01 mol, 1.95 g) in glacial acetic acid (25 mL), the reaction mixture was refluxed for 1 h and then left to cool. The solid product was filtered off, washed with ethanol, dried and finally recrystallized from DMF/H_2_O to afford the corresponding pyrazolo[1,5-a]quinazolines **25a**–**c**.

*2-(4-Methoxyphenylamino)-8,8-dimethyl-6-oxo-N-phenyl-6,7,8,9-tetrahydropyrazolo[1,5-a]quinazoline-3-carboxamide* (**25a**). Yellow crystals, m.p. 270–272 °C, yield (73%). IR (KBr) *ν*_max_/cm^−1^ 3302 (NH), 1655 (C=O). ^1^H-NMR (CDCl_3_, 400 MHz, *δ* ppm): 1.24 (s, 6H, 2CH_3_), 2.58 (s, 2H, CH_2_), 3.31 (s, 2H, CH_2_), 3.82 (s, 3H, OCH_3_), 6.95 (d, 2H, *J* = 9.0 Hz, ArH), 7.14 (t, 1H, ArH), 7.39 (t, 2H, ArH), 7.64 (d, 2H, *J* = 9.0 Hz, ArH), 7.71 (d, 2H, *J* = 7.5 Hz, ArH), 8.99 (s, 1H, quinazoline), 9.48 (s, 1H, NH), 9.88 (s, 1H, NH). ^13^C-NMR (CDCl_3_, 100 MHz, *δ* ppm): 28.7 (2C, 2CH_3_), 32.7 (C, C_8_-quinazoline), 37.7 (C, CH_2_), 51.1 (C, CH_2_), 55.7 (C, OCH_3_), 90.9 (C, C_3_-quinazoline), 113.8 (C, C_5a_-quinazoline), 114.5, 119.6, 120.3, 124.2, 129.2 (9C, Ar), 133.4 (C, C_3a_-quinazoline), 138.2, 147.1, 148.6 (3C, Ar), 151.5 (C, C_2_-quinazoline), 155.0 (C, C_5_-quinazoline), 159.1 (C=O), 162.6 (C, C_9a_-quinazoline), 193.9 (C=O). MS (*m*/*z*, %): 455 (M^+^, 71.06). Anal. Calcd. (%) for C_26_H_25_N_5_O_3_ (455.51): C, 68.56; H, 5.53; N, 15.37. Found: C, 68.50; H, 5.55; N, 15.40%.

*2-(4-Methoxyphenylamino)-8,8-dimethyl-6-oxo-N-(4-methylphenyl)-6,7,8,9-tetrahydropyrazolo[1,5-a]quinazoline-3-carboxamide* (**25b**). Yellow crystals, m.p. 266–268 °C, yield (77%). IR (KBr) *ν*_max_/cm^−1^ 3316 (NH), 1659 (C=O). ^1^H-NMR (CDCl_3_, 400 MHz, *δ* ppm): 1.19 (s, 6H, 2CH_3_), 2.34 (s, 3H, CH_3_), 2.52 (s, 2H, CH_2_), 3.22 (s, 2H, CH_2_), 3.81 (s, 3H, OCH_3_), 6.91 (d, 2H, *J* = 9.0 Hz, ArH), 7.16 (d, 2H, *J* = 8.2 Hz, ArH), 7.54 (d, 2H, *J* = 8.3 Hz, ArH), 7.58 (d, 2H, *J* = 9.0 Hz, ArH), 8.90 (s, 1H, quinazoline), 9.41 (s, 1H, NH), 9.72 (s, 1H, NH). ^13^C-NMR (CDCl_3_, 100 MHz, *δ* ppm): 21.0 (C, CH_3_), 28.7 (2C, 2CH_3_), 32.6 (C, C_8_-quinazoline), 37.5 (C, CH_2_), 50.9 (C, CH_2_), 55.6 (C, OCH_3_), 90.7 (C, C_3_-quinazoline), 113.7 (C, C_5a_-quinazoline), 114.4, 119.4, 120.1, 129.6 (8C, Ar), 133.4 (C, C_3a_-quinazoline), 133.7, 135.7, 146.8, 148.5 (4C, Ar), 151.5 (C, C_2_-quinazoline), 154.8 (C, C_5_-quinazoline), 158.8 (C=O), 162.3 (C, C_9a_-quinazoline), 194.0 (C=O). MS (*m*/*z*, %): 469 (M^+^, 93.88). Anal. Calcd. (%) for C_27_H_27_N_5_O_3_ (469.53): C, 69.07; H, 5.80; N, 14.92. Found: C, 69.15; H, 5.75; N, 15.00%.

*N-(4-Chlorophenyl)-2-(4-methoxyphenylamino)-8,8-dimethyl-6-oxo-6,7,8,9-tetrahydropyrazolo[1,5-a]quinazoline-3-carboxamide* (**25c**). Yellow crystals, m.p. 291–293 °C, yield (72%). IR (KBr) *ν*_max_/cm^−1^ 3299 (NH), 1662 (C=O). ^1^H-NMR (DMSO-*d*_6_, 400 MHz, *δ* ppm): 1.16 (s, 6H, 2CH_3_), 2.59 (s, 2H, CH_2_), 3.36 (s, 2H, CH_2_), 3.76 (s, 3H, OCH_3_), 6.98 (d, 2H, *J* = 8.8 Hz, ArH), 7.45 (d, 2H, *J* = 8.6 Hz, ArH), 7.72 (d, 2H, *J* = 8.6 Hz, ArH), 7.76 (d, 2H, *J* = 8.8 Hz, ArH), 8.95 (s, 1H, quinazoline), 9.30 (s, 1H, NH), 10.01 (s, 1H, NH). ^13^C-NMR (DMSO-*d*_6_, 100 MHz, *δ* ppm): 28.7 (2C, 2CH_3_), 32.6 (C, C_8_-quinazoline), 37.6 (C, CH_2_), 50.0 (C, CH_2_), 55.7 (C, OCH_3_), 90.8 (C, C_3_-quinazoline), 113.7 (C, C_5a_-quinazoline), 114.4, 119.3, 120.1, 129.3 (8C, Ar), 133.4 (C, C_3a_-quinazoline), 133.8, 136.3, 146.9, 148.1 (4C, Ar), 151.5 (C, C_2_-quinazoline), 154.8 (C, C_5_-quinazoline), 158.9 (C=O), 162.4 (C, C_9a_-quinazoline), 193.9 (C=O). MS (*m*/*z*, %): 489 (M^+^, 63.07). Anal. Calcd. (%) for C_26_H_24_ClN_5_O_3_ (489.95): C, 63.74; H, 4.94; N, 14.29. Found: C, 63.80; H, 5.00; N, 14.20%.

### 3.2. Biological Evaluation

#### 3.2.1. In-Vitro Anticancer Activity

Cell culture of HepG-2 (human liver carcinoma) and MCF-7 (human breast adenocarcinoma) cell lines were purchased from the American Type Culture Collection (Rockville, MD, USA) and maintained in DMEM medium which was supplemented with 10% heat-inactivated FBS (fetal bovine serum), 100 U/mL penicillin and 100 U/mL streptomycin. The cells were grown at 37 °C in a humidified atmosphere of 5% CO_2_.

#### 3.2.2. MTT Cytotoxicity Assay

The antitumor activity against HepG-2 and MCF-7 human cancer cell lines was estimated using the 3-[4,5-dimethyl-2-thiazolyl)-2,5-diphenyl-2*H*-tetrazolium bromide (MTT) assay, which is based on the cleavage of the tetrazolium salt by mitochondrial dehydrogenases in viable cells [31,32,33]. Cells were dispensed in a 96 well sterile microplate (5 × 10^4^ cells/well), and incubated at 37 °C with series of different concentrations, in DMSO, of each tested compound or Doxorubicin^®^ (positive control) for 48 h in a serum free medium prior to the MTT assay. After incubation, media were carefully removed, 40 µL of MTT (2.5 mg/mL) were added to each well and then incubated for an additional 4 h. The purple formazan dye crystals were solubilized by the addition of 200 µL of DMSO. The absorbance was measured at 590 nm using a SpectraMax^®^, Paradigm^®^ Multi-Mode microplate reader. The relative cell viability was expressed as the mean percentage of viable cells compared to the untreated control cells.

#### 3.2.3. Statistical Analysis

All experiments were conducted in triplicate and repeated on three different days. All the values were represented as mean ± SD. IC_50_s were determined by probit analysis using the SPSS software program (SPSS Inc., Chicago, IL, USA).

#### 3.2.4. Cell Cycle Analysis and Apoptosis Detection

Cell cycle analysis and apoptosis detection were carried out by flow cytometry [35]. Both HepG-2 and MCF-7 cells were seeded at 8 × 10^4^ and incubated at 37 °C, 5% CO_2_ overnight, after treatment with the tested compounds, for 24 h. Cell pellets were collected and centrifuged (300 *g*, 5 min). For cell cycle analysis, cell pellets were fixed with 70% ethanol on ice for 15 min and collected again. The collected pellets were incubated with propidium iodide (PI) staining solution (50 µg/mL PI, 0.1 mg/mL RNaseA, 0.05% Triton X-100) at room temperature for 1 h and analyzed by Gallios flow cytometer (Beckman Coulter, Brea, CA, USA). Apoptosis detection was performed by FITC Annexin-V/PI commercial kit (Becton Dickenson, Franklin Lakes, NJ, USA) following the manufacture protocol. The samples were analyzed by fluorescence-activated cell sorting (FACS) with a Gallios flow cytometer (Beckman Coulter) within 1 h after staining. Data were analyzed using Kaluza v. 1.2 (Beckman Coulter).

## 4. Conclusions

A series of *N*-aryl-7-aryl-pyrazolo[1,5-*a*]pyrimidines **18a**–**u** and *N*-aryl-pyrazolo[1,5-*a*]quinazolines **25a**–**c** have been synthesized and investigated for their in vitroantitumor activity. All the investigated compounds showed dose-dependent cytotoxic activities against two cancer types (liver and breast cancer). The IC_50_ values of these compounds did not reveal statistical significant differences compared to the positive control (doxorubicin). Therefore, two compounds (**18o** and **18a**) have been selected to study their cell cycle and apoptotic effect against HepG2 and MCF-7 cancer cell lines. Compounds **18o** and **18a**showed slightly higher cytotoxicity compared to doxorubicin against HepG-2 cells (IC_50_ = 72.2 ± 3.8 vs. 80.9 ± 2.1 µM) and against MCF-7 cells (IC_50_ = 63.1 ± 3.1 vs. 65.6 ± 4.2µM), respectively. Cell cycle analysis of HepG-2 cells treated with **18o** and MCF-7 cells treated with **18a** revealed a significant G2/M phase arrest coupled with an increase in the percentage of cells in pre-G phase, which is indicative of apoptosis. The pro-apoptotic activity of **18a** and **18o** was inferred by the significant increase in the percentage of annexin V-FITC-positive apoptotic cells.

## Figures and Tables

**Table 1:**
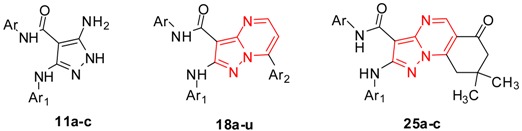
The IC_50_ (µM) values of compounds **11a**–**c**, **18a**–**u** and **25a**–**c** using MTT assay against two human carcinoma cell lines (HepG-2 and MCF-7).

Compounds	Ar	Ar_1_	Ar_1_	IC_50_ (µM)	
				HepG-2	MCF-7
**11a**	C_6_H_5_	4-CH_3_O-C_6_H_4_	-	81.3 ± 4.1	65.5 ± 4.3
**11b**	4-CH_3_-C_6_H_4_	4-CH_3_O-C_6_H_4_	-	86.2 ± 4.5	69.2 ± 3.9
**11c**	4-Cl-C_6_H_4_	4-CH_3_O-C_6_H_4_	-	94.8 ± 6.5	69.1 ± 3.7
**18a**	C_6_H_5_	4-CH_3_O-C_6_H_4_	C_6_H_5_	85.4 ± 5.1	63.1 ± 3.1 *
**18b**	C_6_H_5_	4-CH_3_O-C_6_H_4_	4-CH_3_-C_6_H_4_	90.9 ± 6.5	64.9 ± 3.1
**18c**	C_6_H_5_	4-CH_3_O-C_6_H_4_	4-CH_3_O-C_6_H_4_	75.9 ± 5.3	64.3 ± 4.2
**18d**	C_6_H_5_	4-CH_3_O-C_6_H_4_	4-Cl-C_6_H_4_	77.1 ± 4.2	65.1 ± 2.8
**18e**	C_6_H_5_	4-CH_3_O-C_6_H_4_	4-Br-C_6_H_4_	81.2 ± 5.5	68.1 ± 4.0
**18f**	C_6_H_5_	4-CH_3_O-C_6_H_4_	4-F-C_6_H_4_	92.8 ± 6.7	66.1 ± 2.9
**18g**	C_6_H_5_	4-CH_3_O-C_6_H_4_	thiophen-2-yl	91.1 ± 6.4	69.2 ± 3.2
**18h**	4-CH_3_-C_6_H_4_	4-CH_3_O-C_6_H_4_	C_6_H_5_	73.2 ± 3.2	66.8 ± 2.6
**18i**	4-CH_3_-C_6_H_4_	4-CH_3_O-C_6_H_4_	4-CH_3_-C_6_H_4_	83.3 ± 4.3	67.7 ± 2.7
**18j**	4-CH_3_-C_6_H_4_	4-CH_3_O-C_6_H_4_	4-CH_3_O-C_6_H_4_	77.4 ± 2.9	64.3 ± 3.1
**18k**	4-CH_3_-C_6_H_4_	4-CH_3_O-C_6_H_4_	4-Cl-C_6_H_4_	74.0 ± 3.1	66.8 ± 3.9
**18l**	4-CH_3_-C_6_H_4_	4-CH_3_O-C_6_H_4_	4-Br-C_6_H_4_	78.7 ± 5.1	66.7 ± 3.2
**18m**	4-CH_3_-C_6_H_4_	4-CH_3_O-C_6_H_4_	4-F-C_6_H_4_	80.3 ± 3.9	66.2 ± 3.8
**18n**	4-CH_3_-C_6_H_4_	4-CH_3_O-C_6_H_4_	thiophen-2-yl	82.5 ± 5.7	65.9 ± 3.1
**18o**	4-Cl-C_6_H_4_	4-CH_3_O-C_6_H_4_	C_6_H_5_	72.2 ± 3.8 *	64.7 ± 1.9
**18p**	4-Cl-C_6_H_4_	4-CH_3_O-C_6_H_4_	4-CH_3_-C_6_H_4_	87.8 ± 5.4	67.1 ± 2.1
**18q**	4-Cl-C_6_H_4_	4-CH_3_O-C_6_H_4_	4-CH_3_O-C_6_H_4_	72.8 ± 3.9	65.5 ± 2.1
**18r**	4-Cl-C_6_H_4_	4-CH_3_O-C_6_H_4_	4-Cl-C_6_H_4_	73.0 ± 1.9	65.9 ± 2.6
**18s**	4-Cl-C_6_H_4_	4-CH_3_O-C_6_H_4_	4-Br-C_6_H_4_	78.2 ± 3.2	66.8 ± 5.0
**18t**	4-Cl-C_6_H_4_	4-CH_3_O-C_6_H_4_	4-F-C_6_H_4_	78.7 ± 4.7	67.0 ± 1.8
**18u**	4-Cl-C_6_H_4_	4-CH_3_O-C_6_H_4_	thiophen-2-yl	83.1 ± 5.1	64.5 ± 2.9
**25a**	C_6_H_5_	4-CH_3_O-C_6_H_4_	-	87.9 ± 6.0	68.9 ± 4.2
**25b**	4-CH_3_-C_6_H_4_	4-CH_3_O-C_6_H_4_	-	81.9 ± 5.9	66.2 ± 2.9
**25c**	4-Cl-C_6_H_4_	4-CH_3_O-C_6_H_4_	-	79.5 ± 4.8	66.5 ± 3.1
**Doxorubicin**	-	-	-	80.9 ± 2.1	65.6 ± 4.2

* The most potent compound and selected for further experiments.

## Supplementary Materials

Spectra of compounds are available online.
