# Supplementary material for: Design, Synthesis and Antitumor Evaluation of Novel Pyrazolopyrimidines and Pyrazoloquinazolines
Source: Molecules. 2018 May 23;23(6):1249. doi: 10.3390/molecules23061249 (PMC6100441; doi:10.3390/molecules23061249)
Supplement: Supplementary file 1 [file molecules-23-01249-s001.pdf]

SUPPLEMENTARY MATERIAL

for

Design, synthesis and antitumor evaluation of novel pyrazolopyrimidines and pyrazoloquinazolines

Mohamed El-Naggar[1], Ashraf S. Hassan[2,*] Hanem M. Awad[3] and Mohamed F. Mady[4,5,*]

[1] Chemistry Department, Faculty of Sciences, University of Sharjah, Sharjah, UAE, 27272; M5elnaggar@yahoo.com
[2] Organometallic and Organometalloid Chemistry Department, National Research Centre, Dokki 12622 Cairo, Egypt; ashraf_salmoon@yahoo.com
[3] Department of Tanning Materials and Leather Technology, National Research Centre, Dokki 12622, Cairo, Egypt; hanem_awad@yahoo.com
[4] Department of Green Chemistry, National Research Centre, Dokki 12622, Cairo, Egypt
[5] Department of Mathematics and Natural Science, Faculty of Science and Technology, University of Stavanger, N-4036 Stavanger, Norway; mohamed.mady@uis.no

* Authors to whom correspondence should be addressed; E-Mails: ashraf_salmoon@yahoo.com (A.S.H); mohamed.mady@uis.no (M.F.M); Tel.: +20-100-664-5444 (A. S. H.); +47-912-569-33 (M. F. M.).

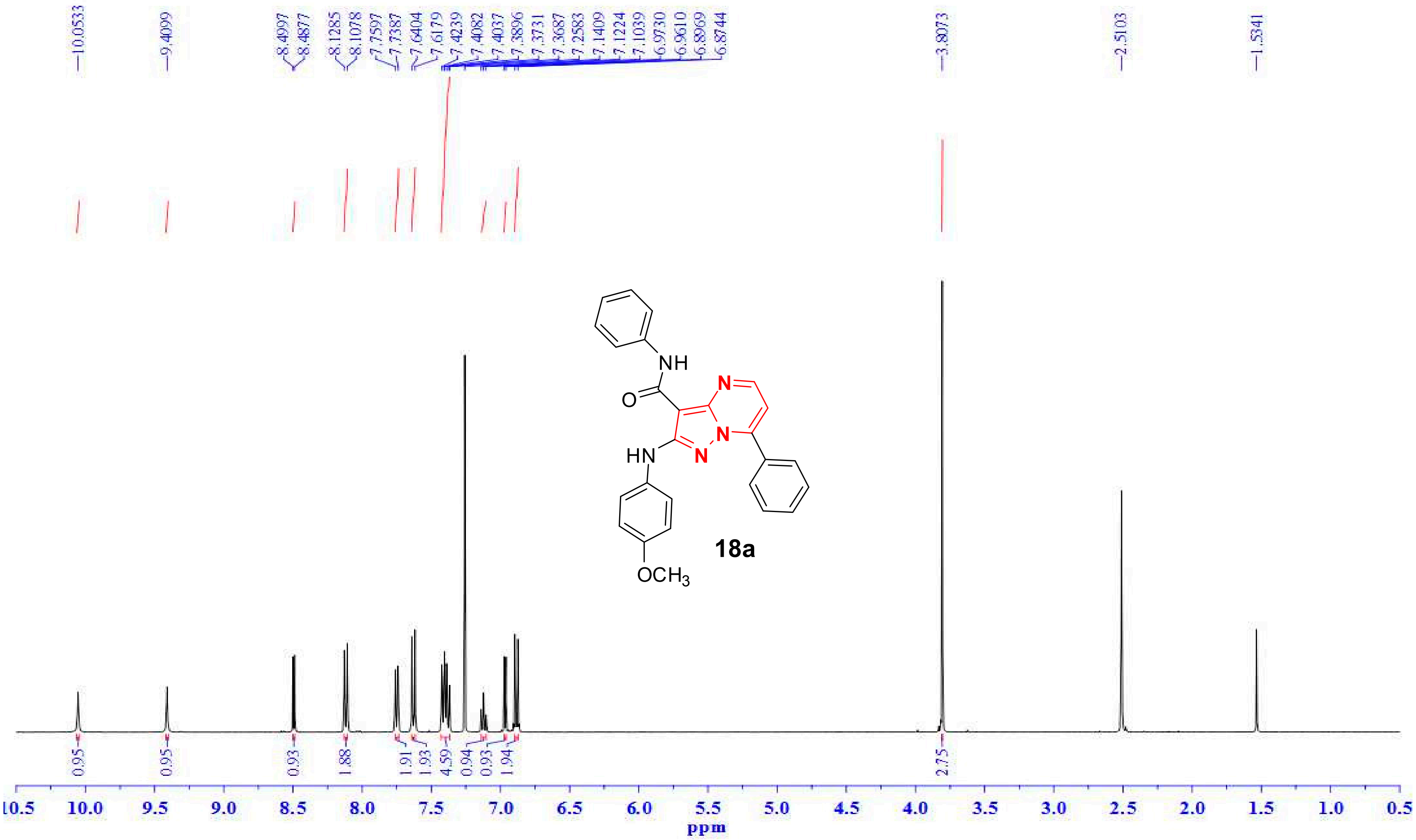

The ^{1}H NMR (400 MHz) spectrum of compound **18a**

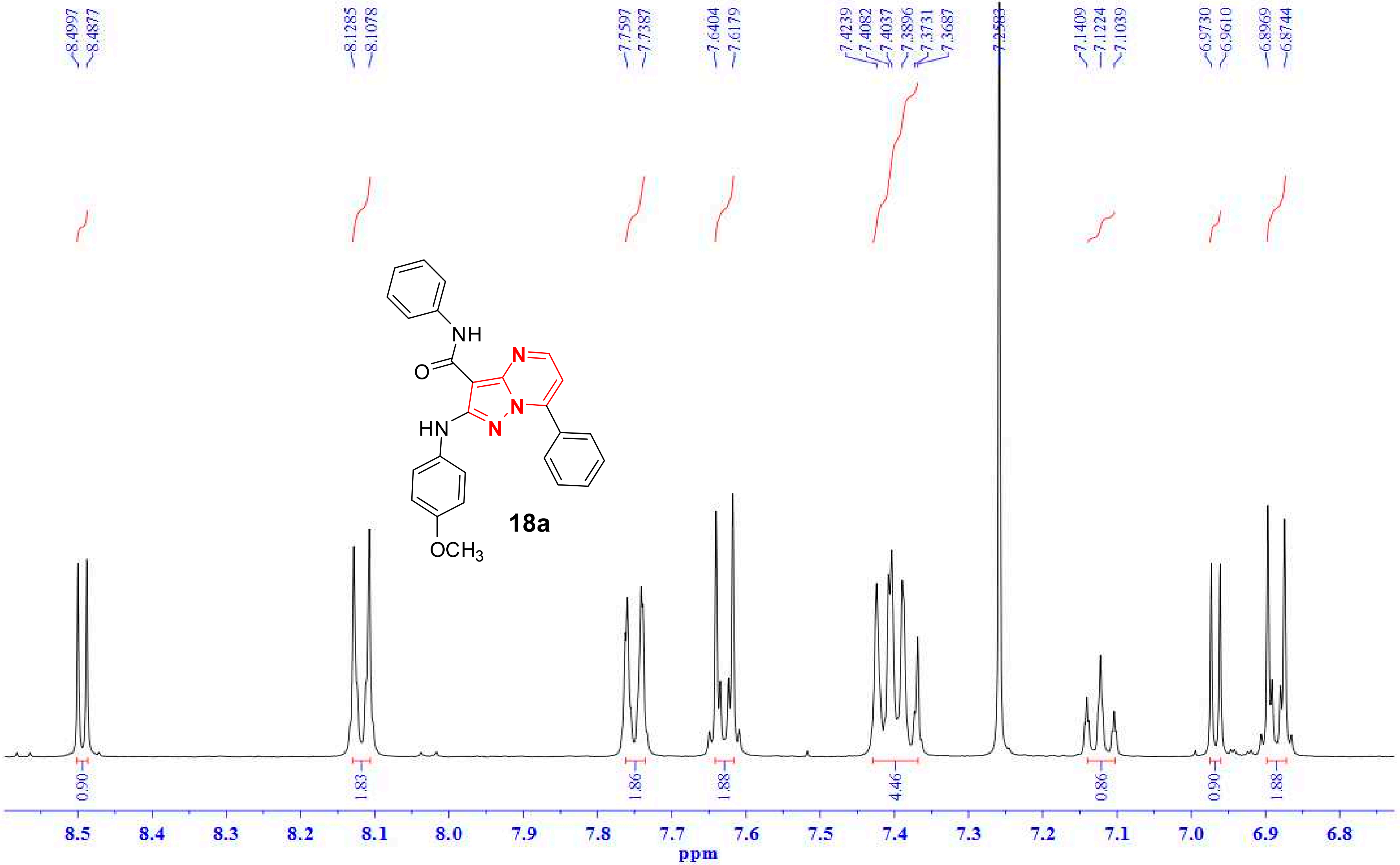

18a

The ^{1}H NMR (400 MHz) spectrum (aromatic region) of compound **18a**

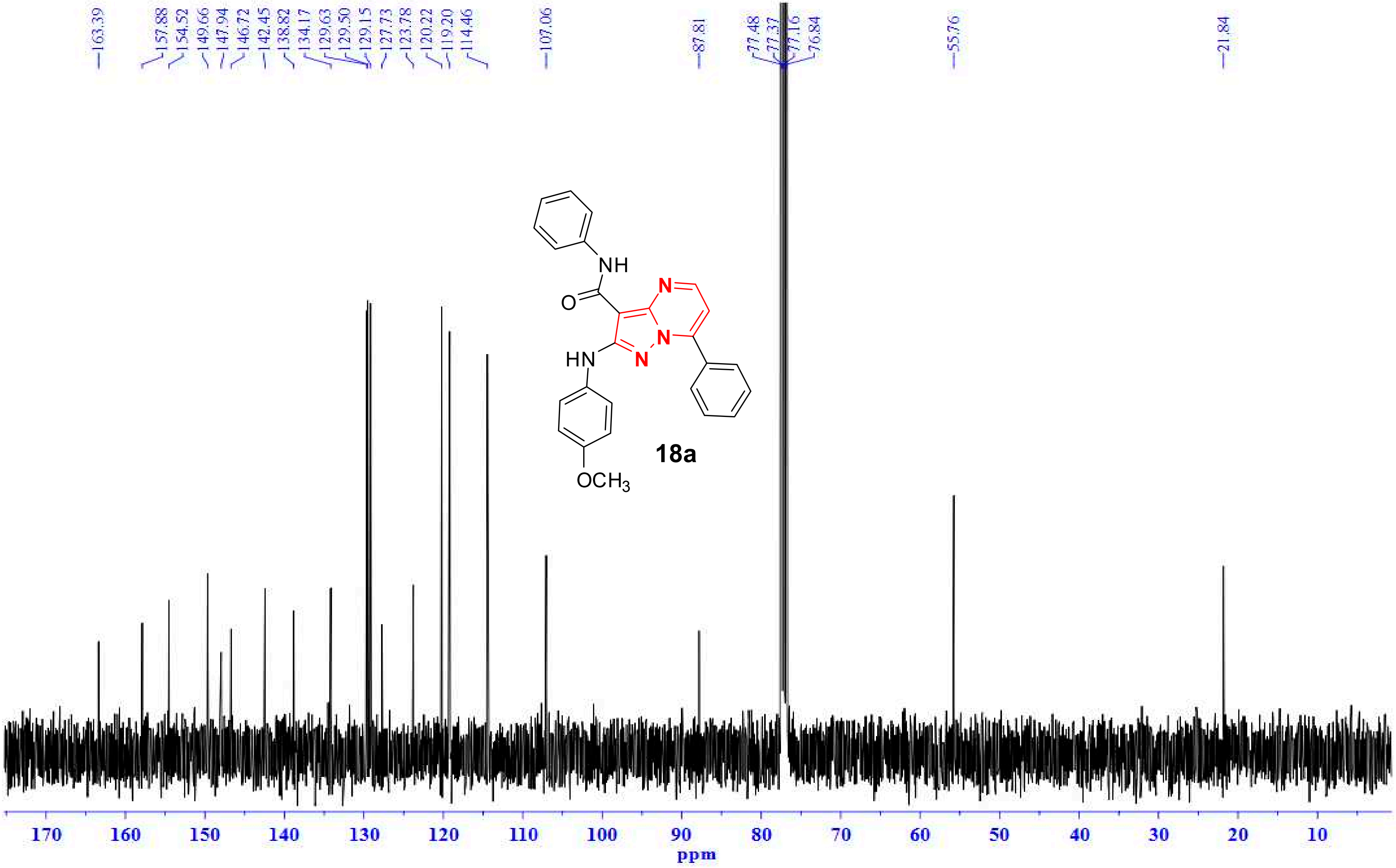

The ^{13}C NMR (100 MHz) spectrum of compound **18a**

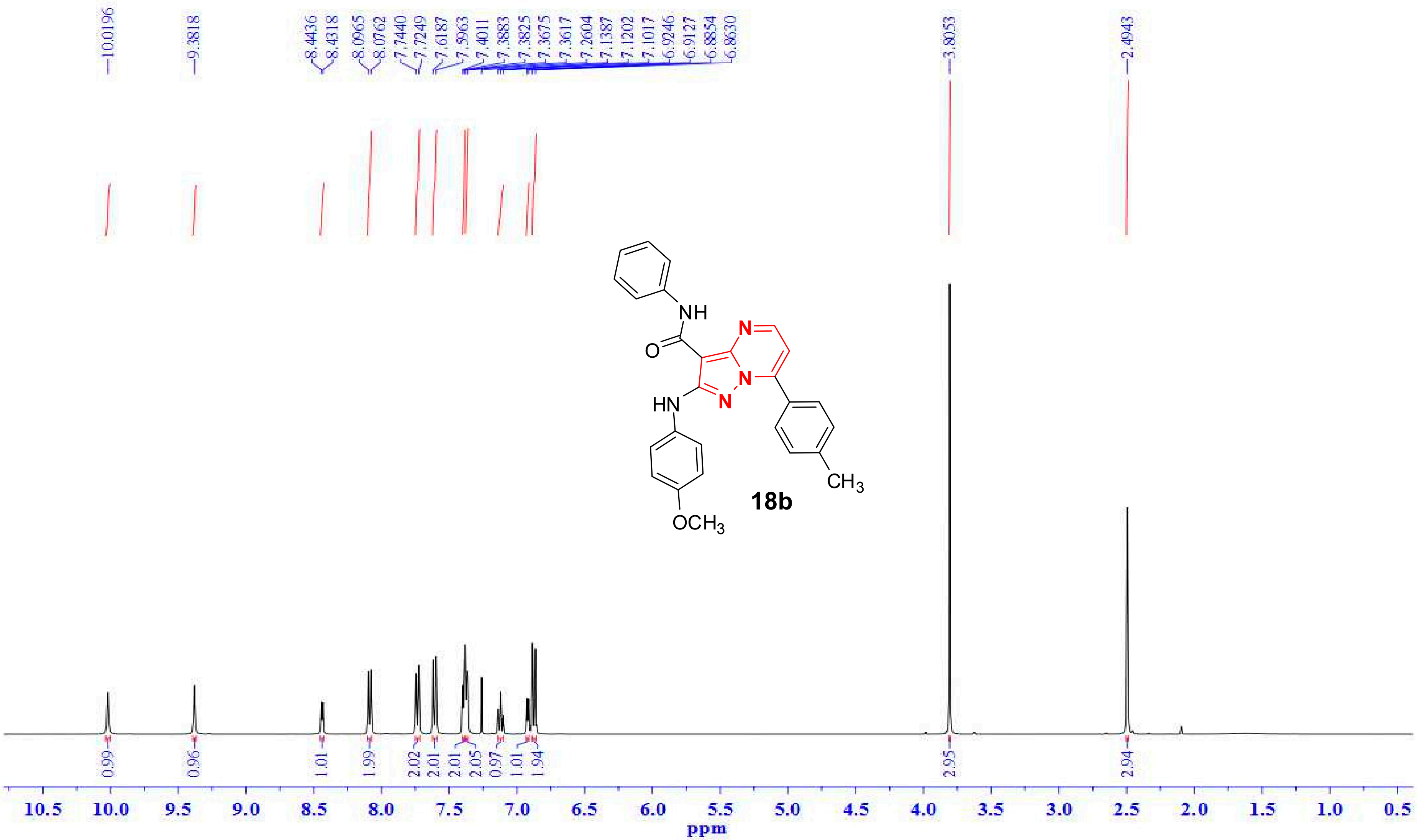

The ^{1}H NMR (400 MHz) spectrum of compound **18b**

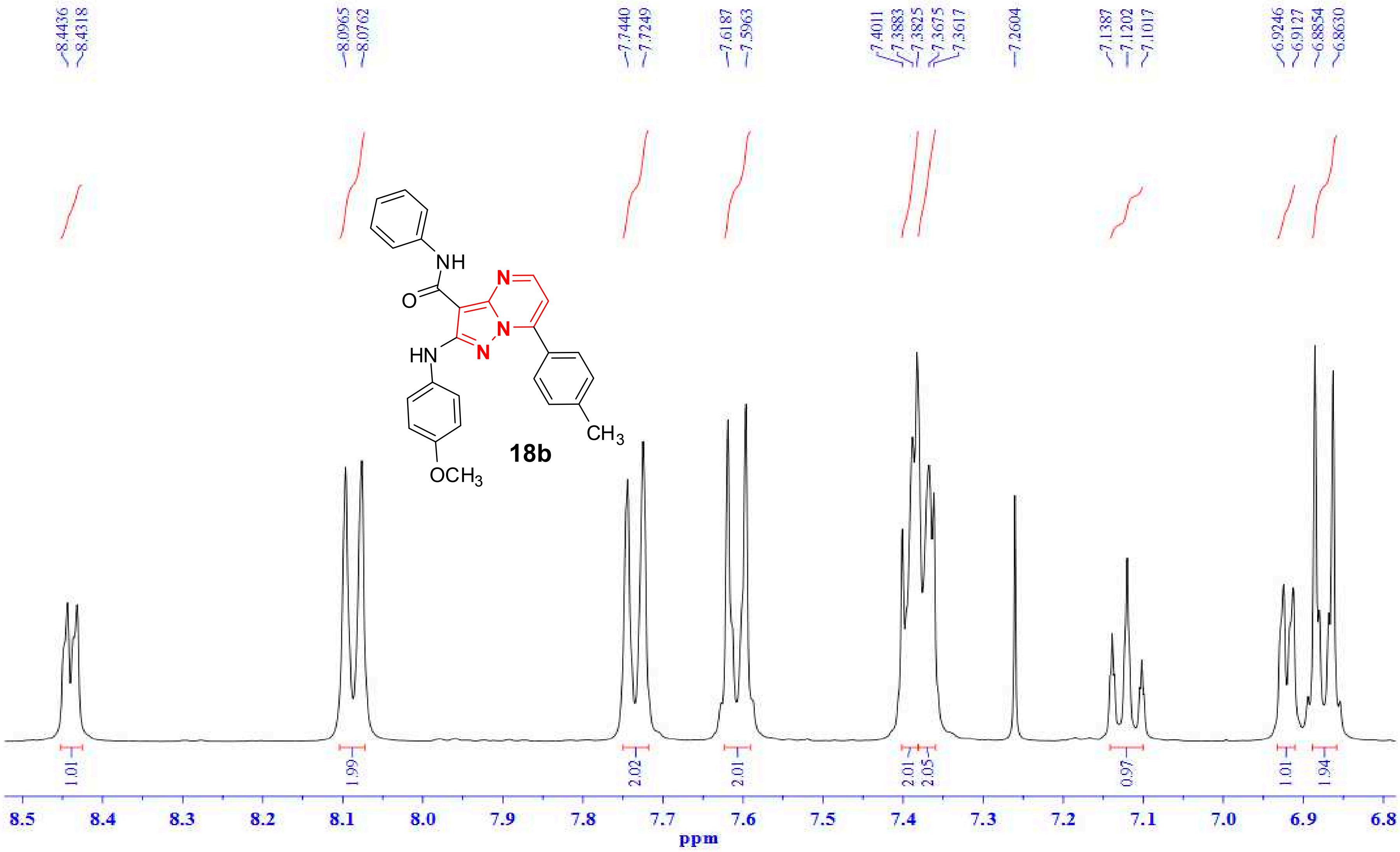

The ¹H NMR (400 MHz) spectrum (aromatic region) of compound **18b**

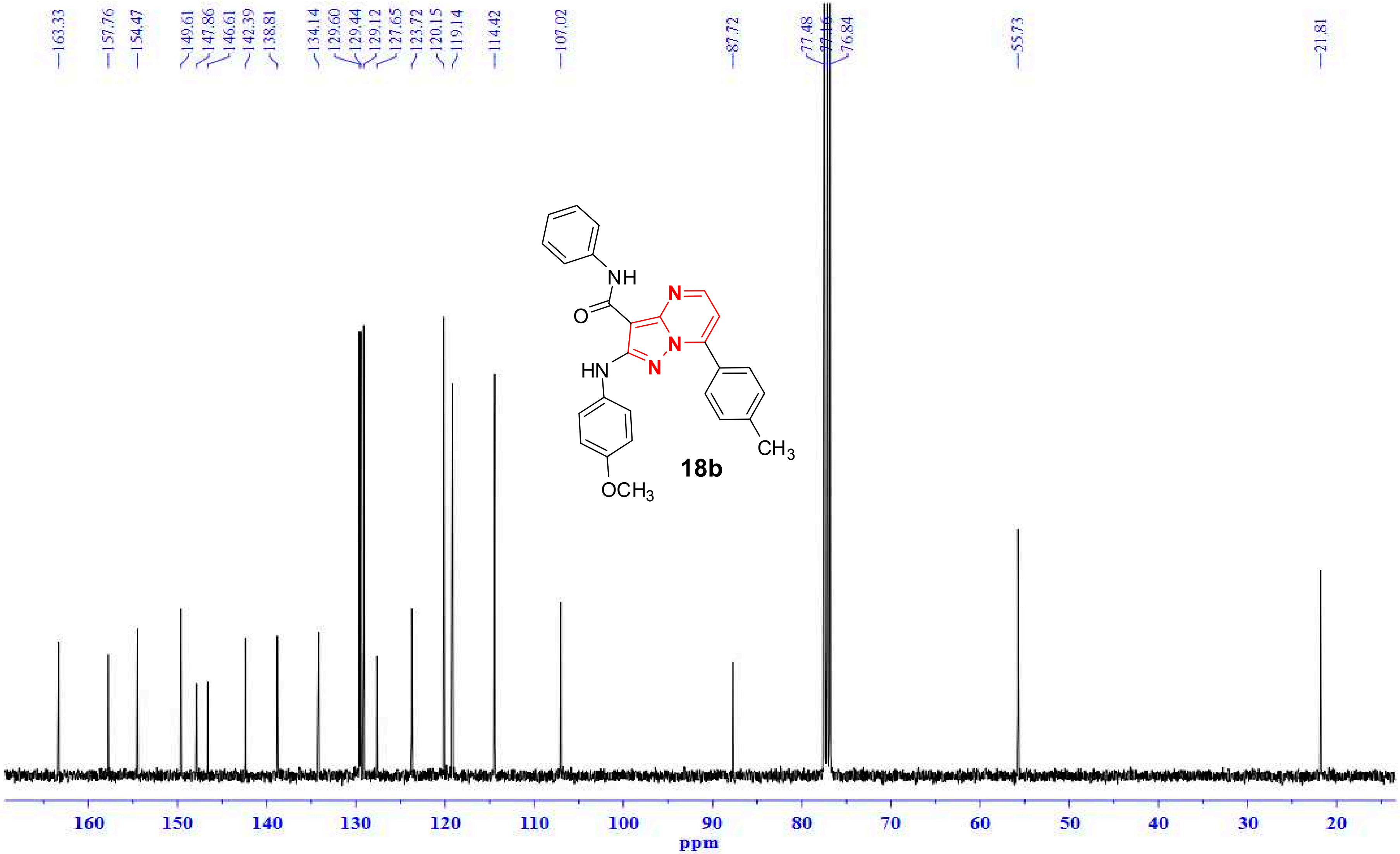

The ^{13}C NMR (100 MHz) spectrum of compound **18b**

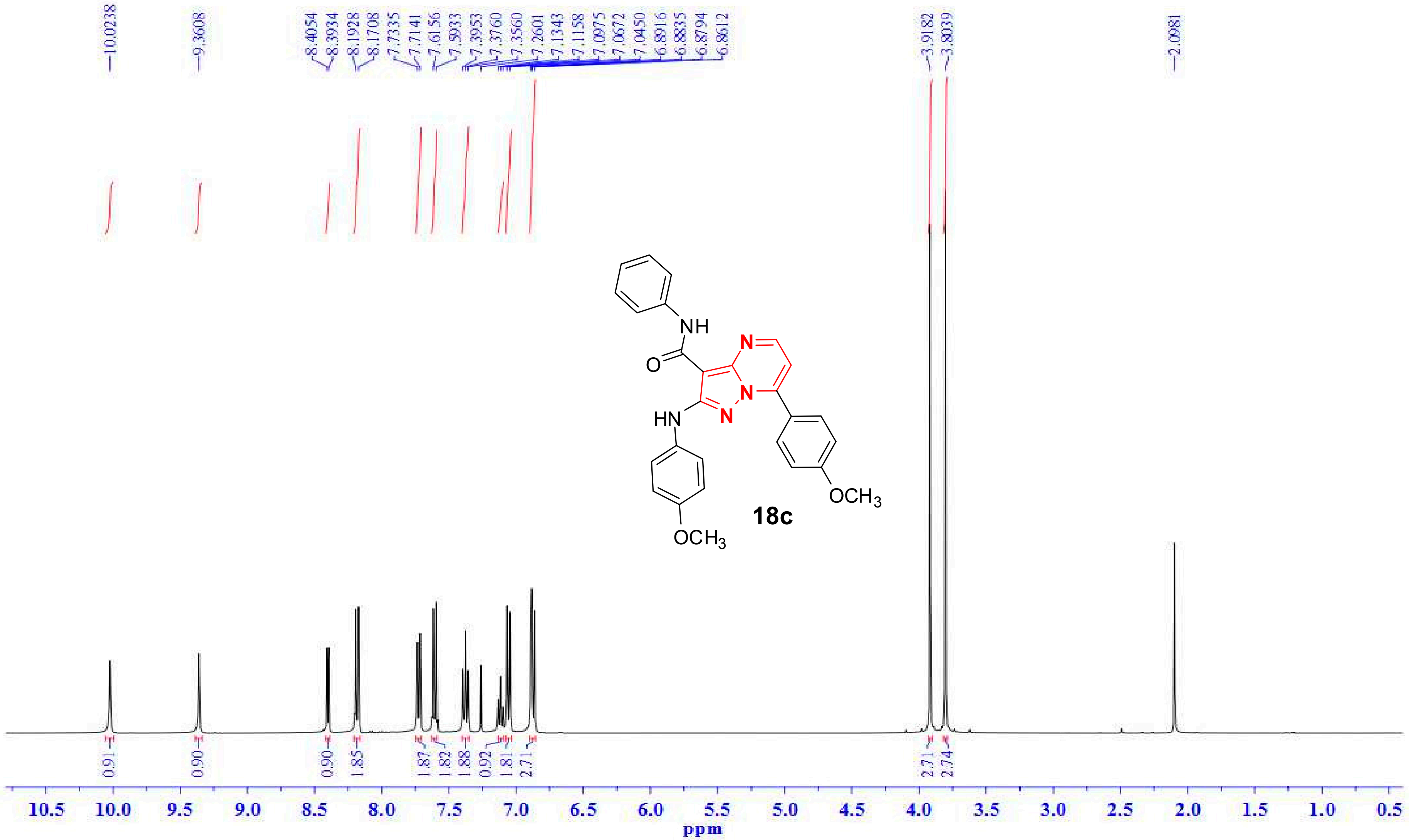

The ^{1}H NMR (400 MHz) spectrum of compound **18c**

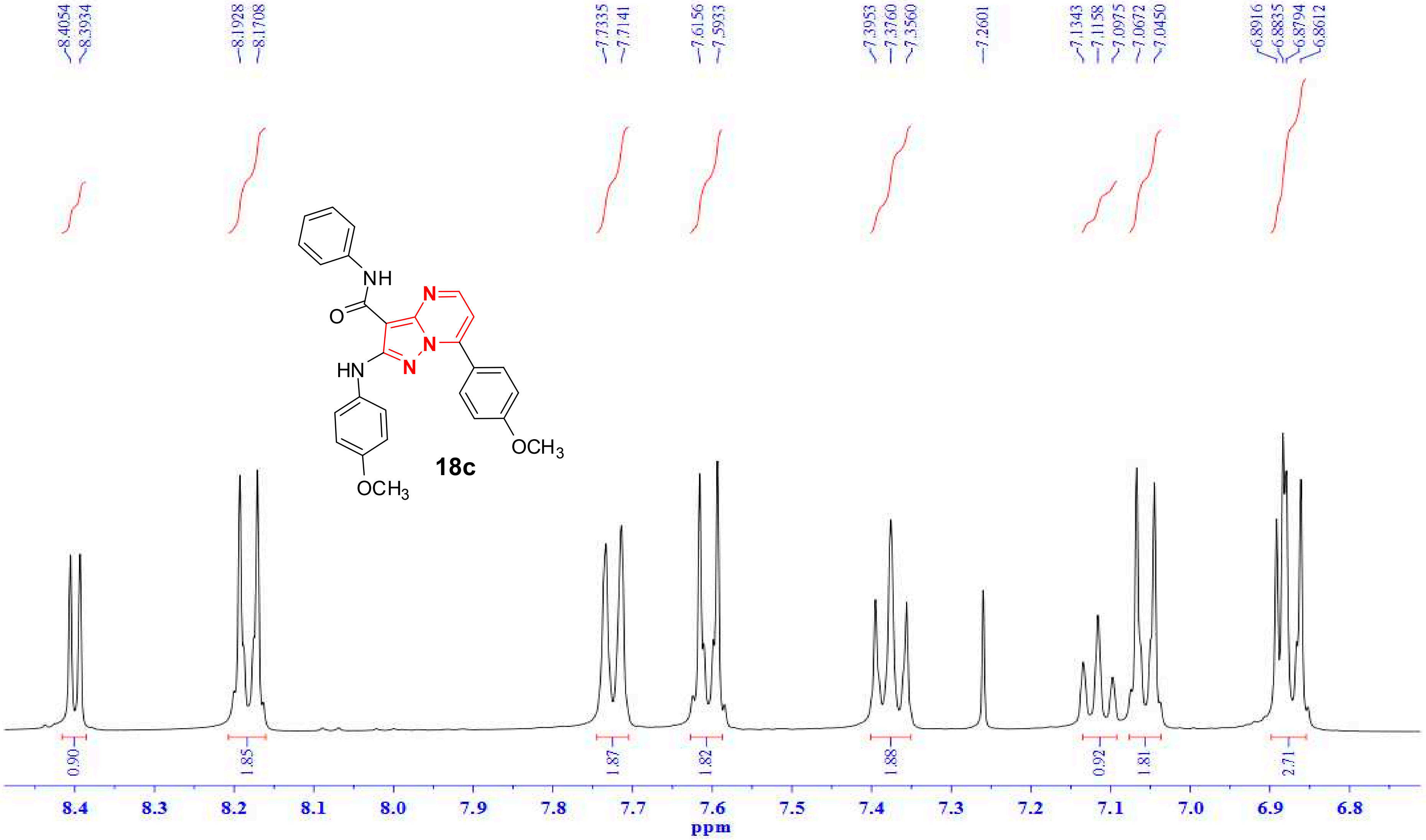

The ^{1}H NMR (400 MHz) spectrum (aromatic region) of compound **18c**

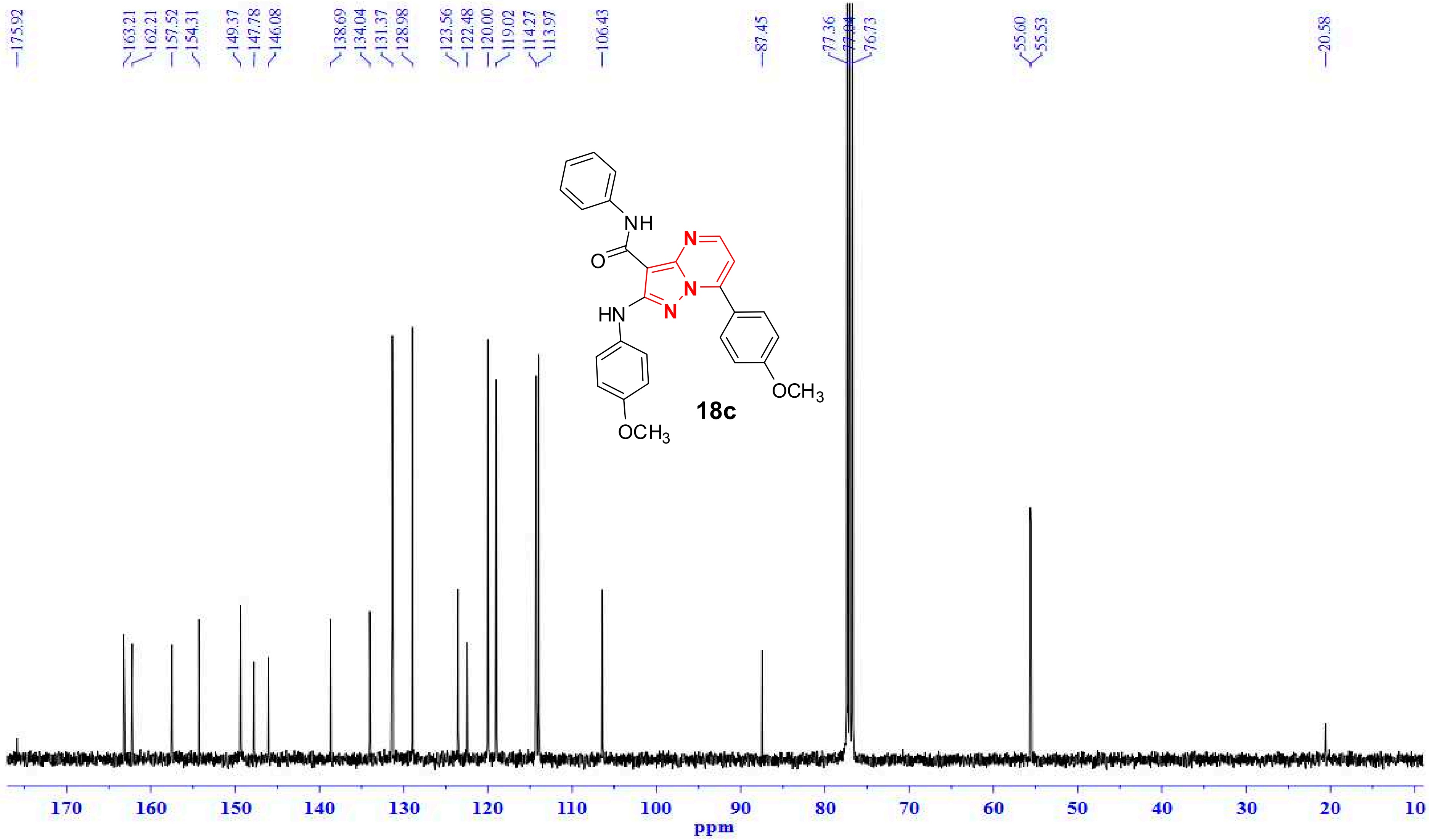

The ^{13}C NMR (100 MHz) spectrum of compound **18c**

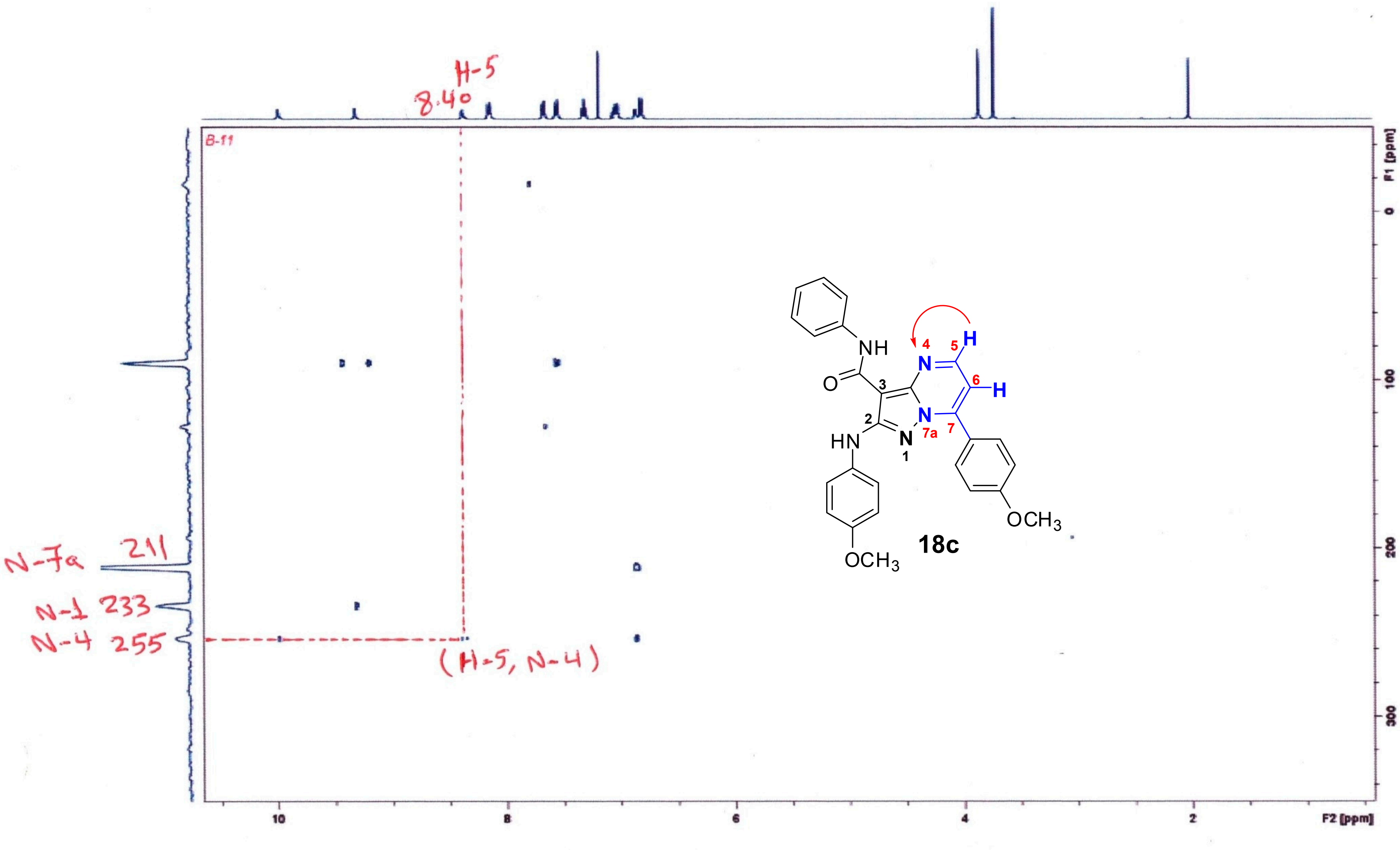

The ^1H-^{15}N HMBC (40 MHz) spectrum of compound **18c**

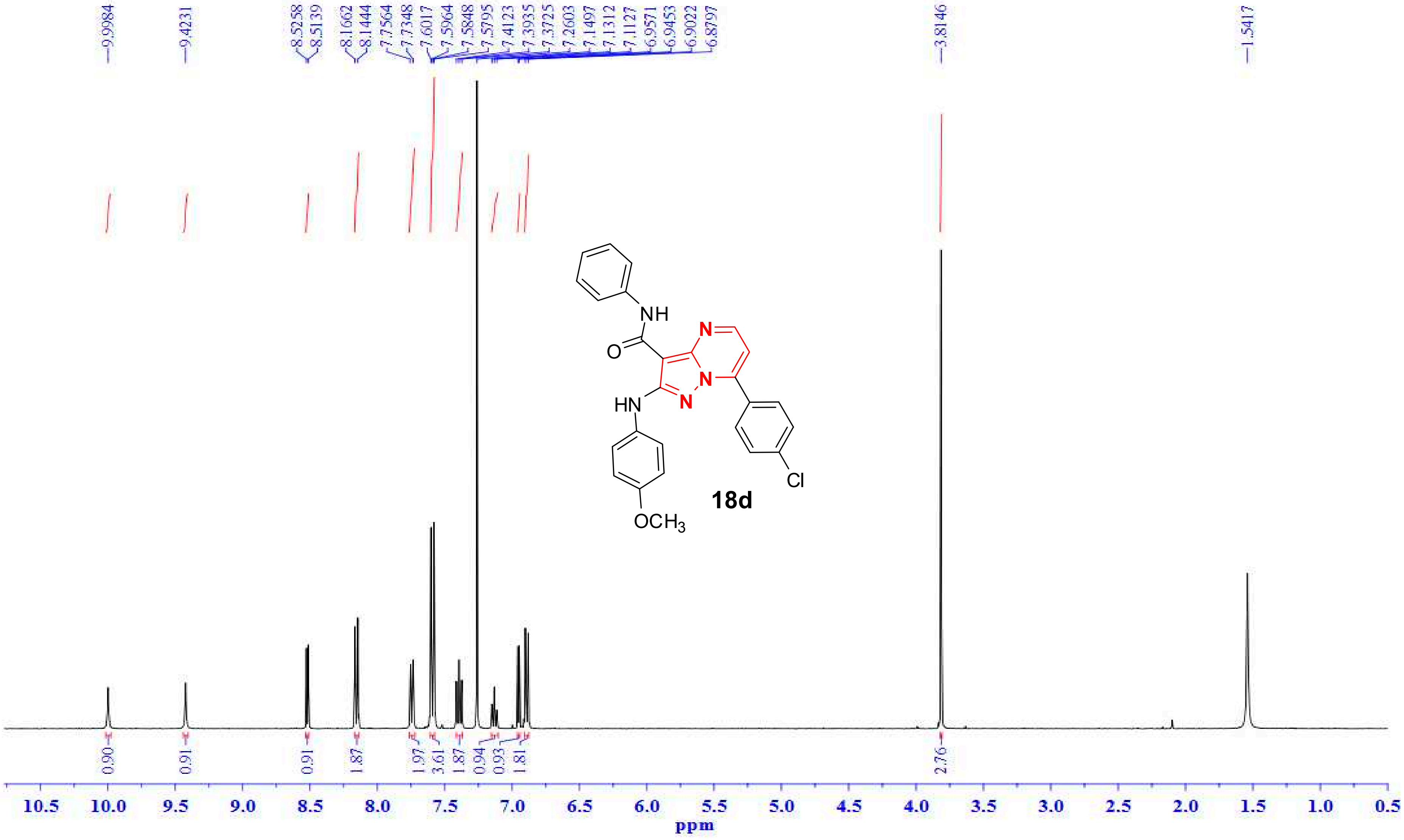

The ^{1}H NMR (400 MHz) spectrum of compound **18d**

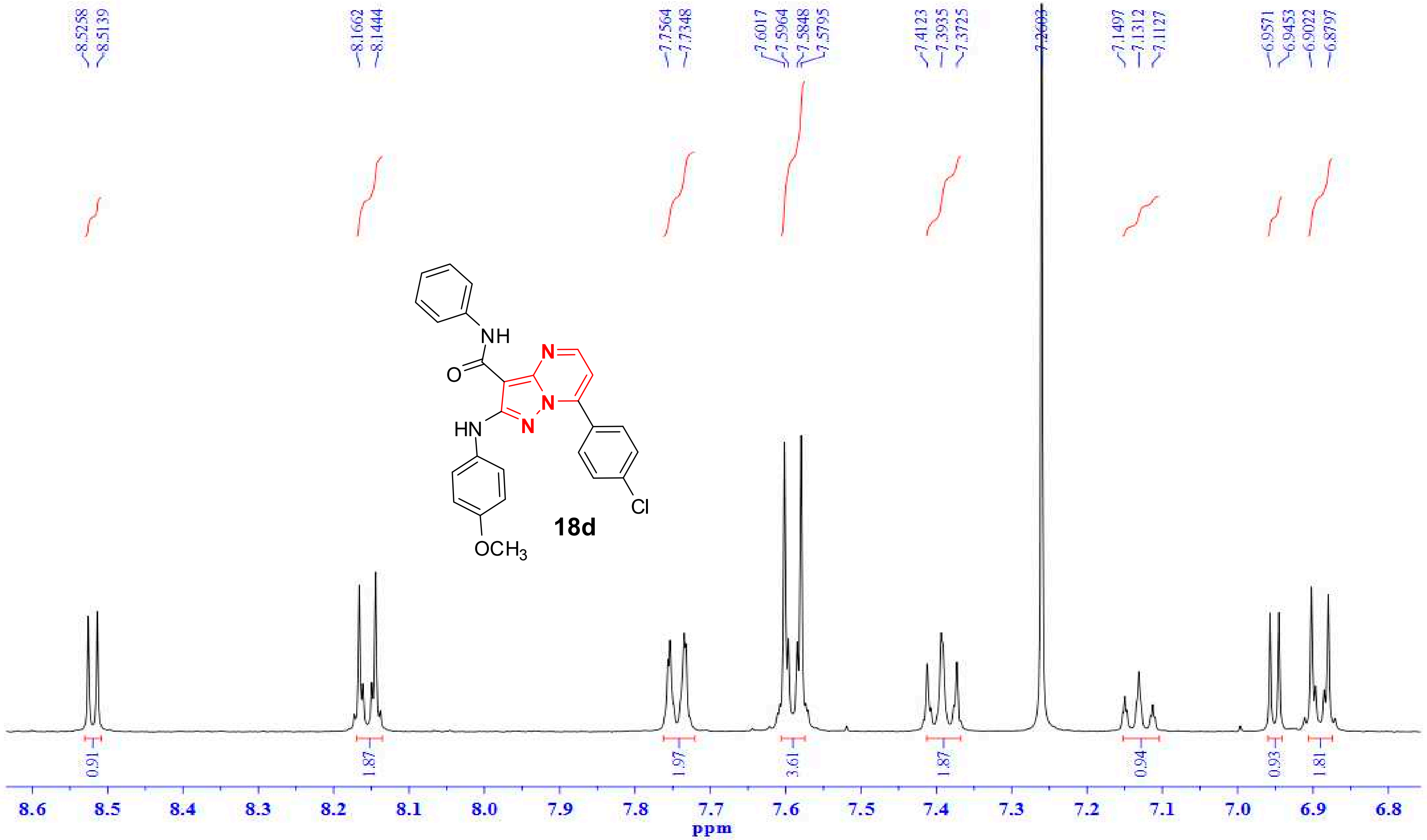

The ^{1}H NMR (400 MHz) spectrum (aromatic region) of compound **18d**

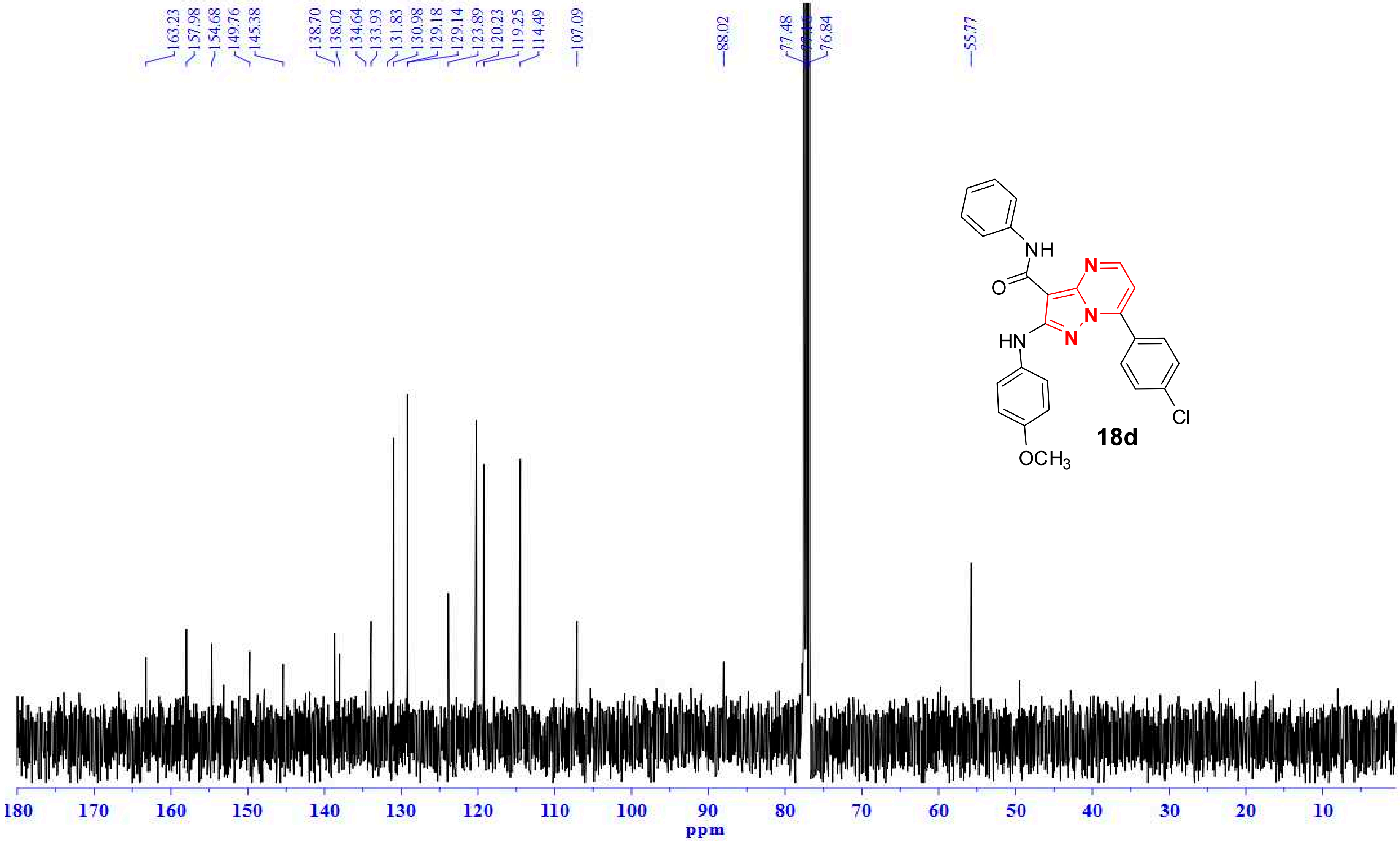

The ^{13}C NMR (100 MHz) spectrum of compound **18d**

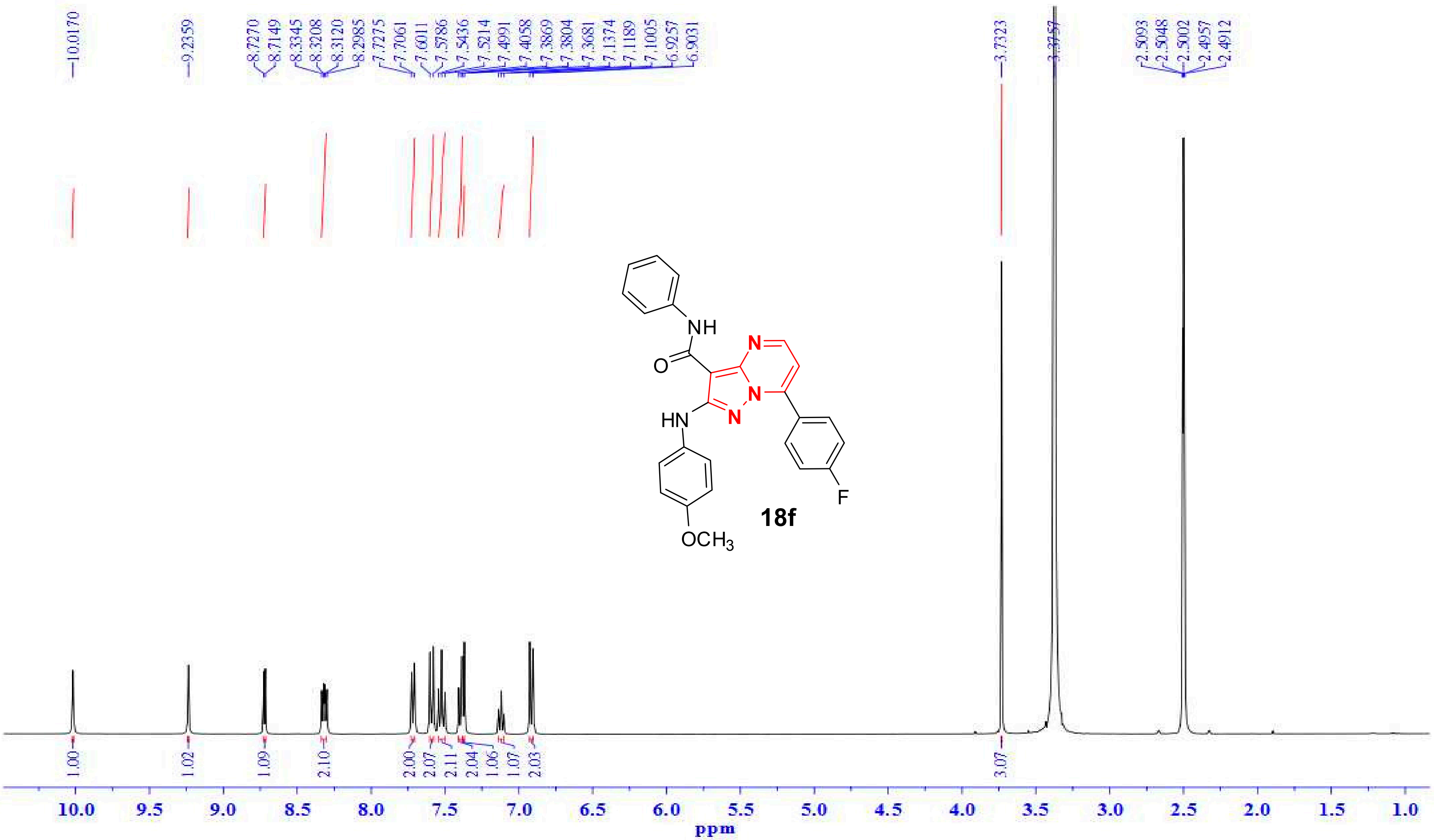

The ^{1}H NMR (400 MHz) spectrum of compound **18f**

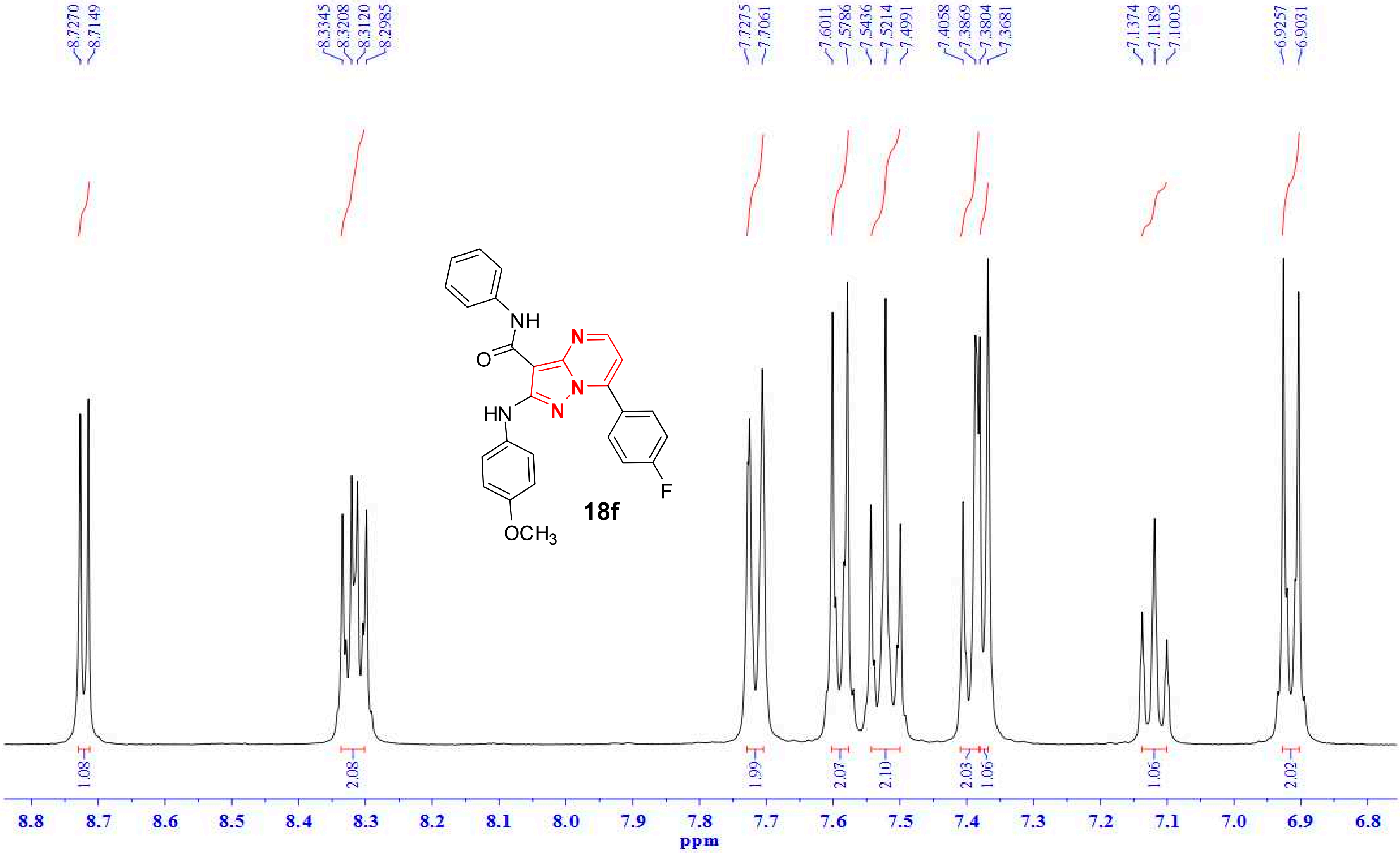

The ¹H NMR (400 MHz) spectrum (aromatic region) of compound **18f**

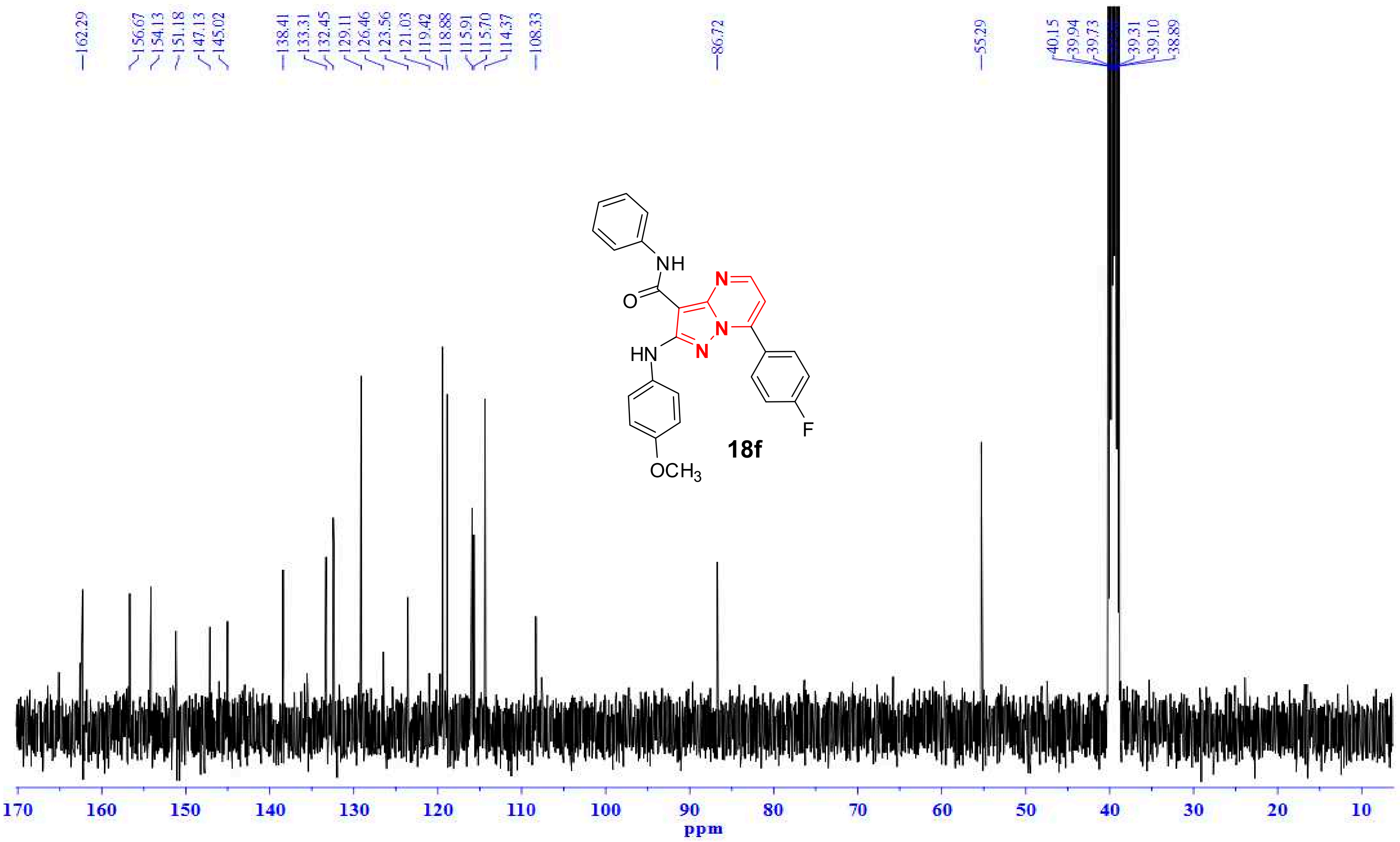

The ^{13}C NMR (100 MHz) spectrum of compound **18f**

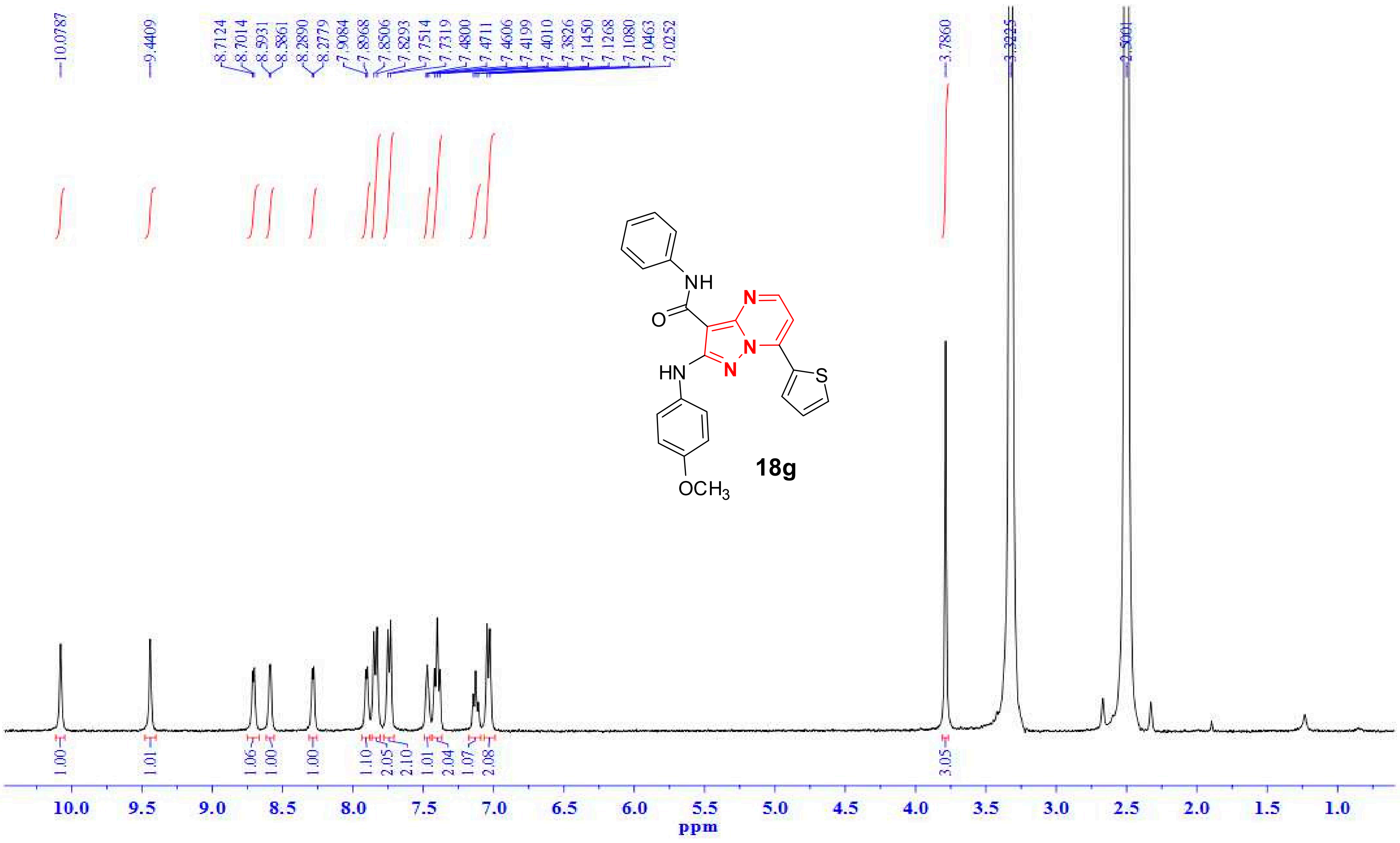

The ^{1}H NMR (400 MHz) spectrum of compound **18g**

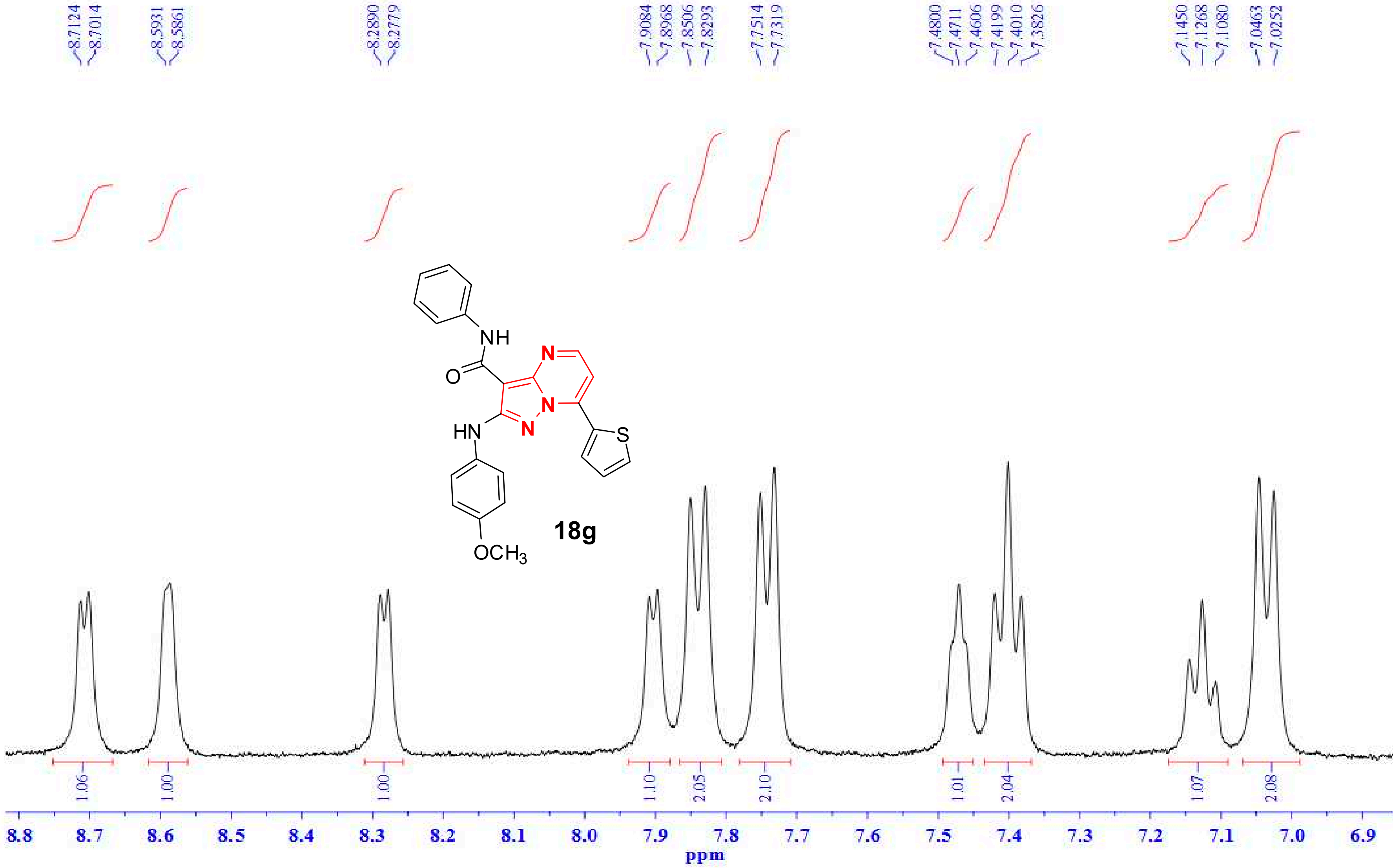

The ^{1}H NMR (400 MHz) spectrum (aromatic region) of compound **18g**

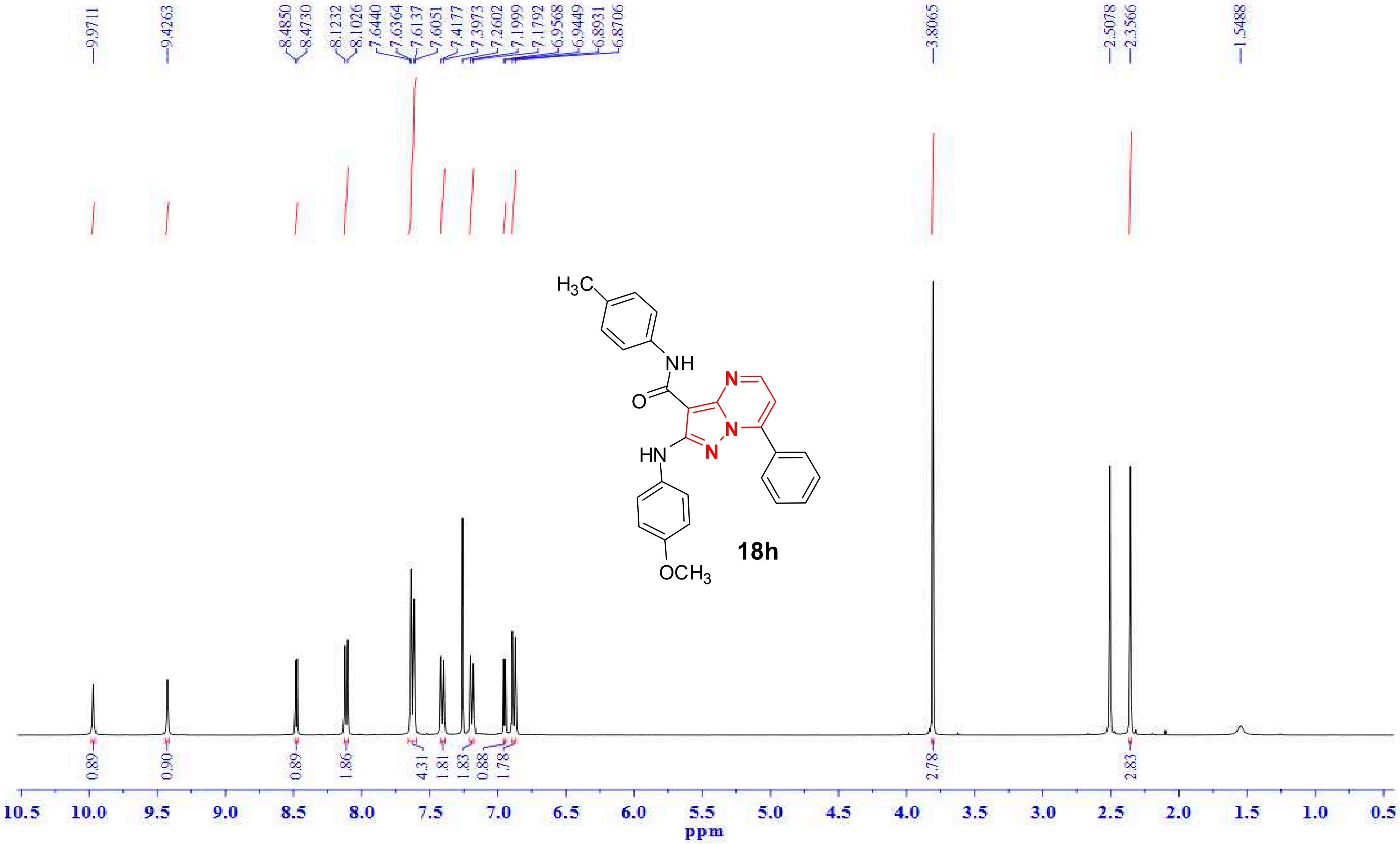

The ^{1}H NMR (400 MHz) spectrum of compound **18h**

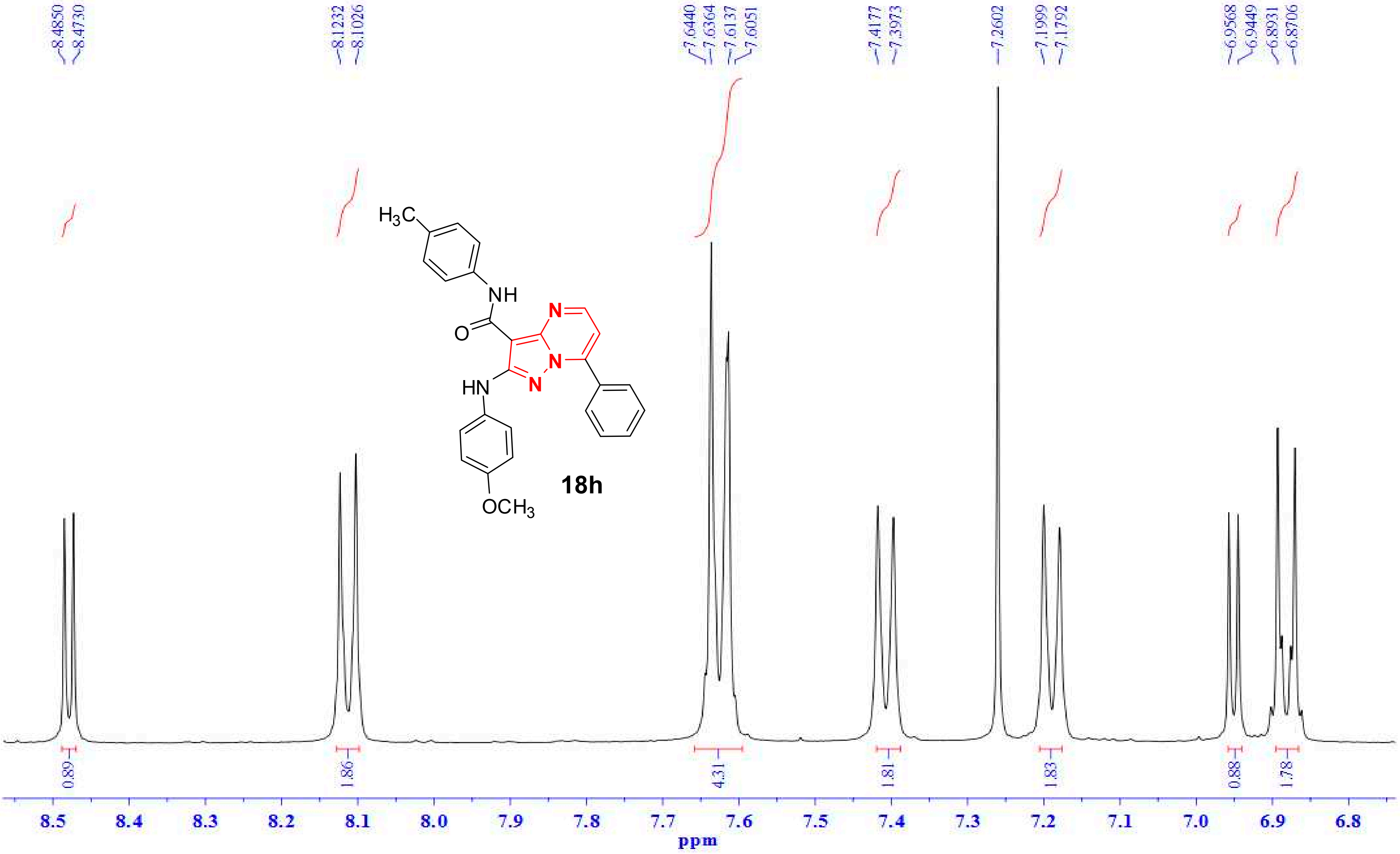

The ^{1}H NMR (400 MHz) spectrum (aromatic region) of compound **18h**

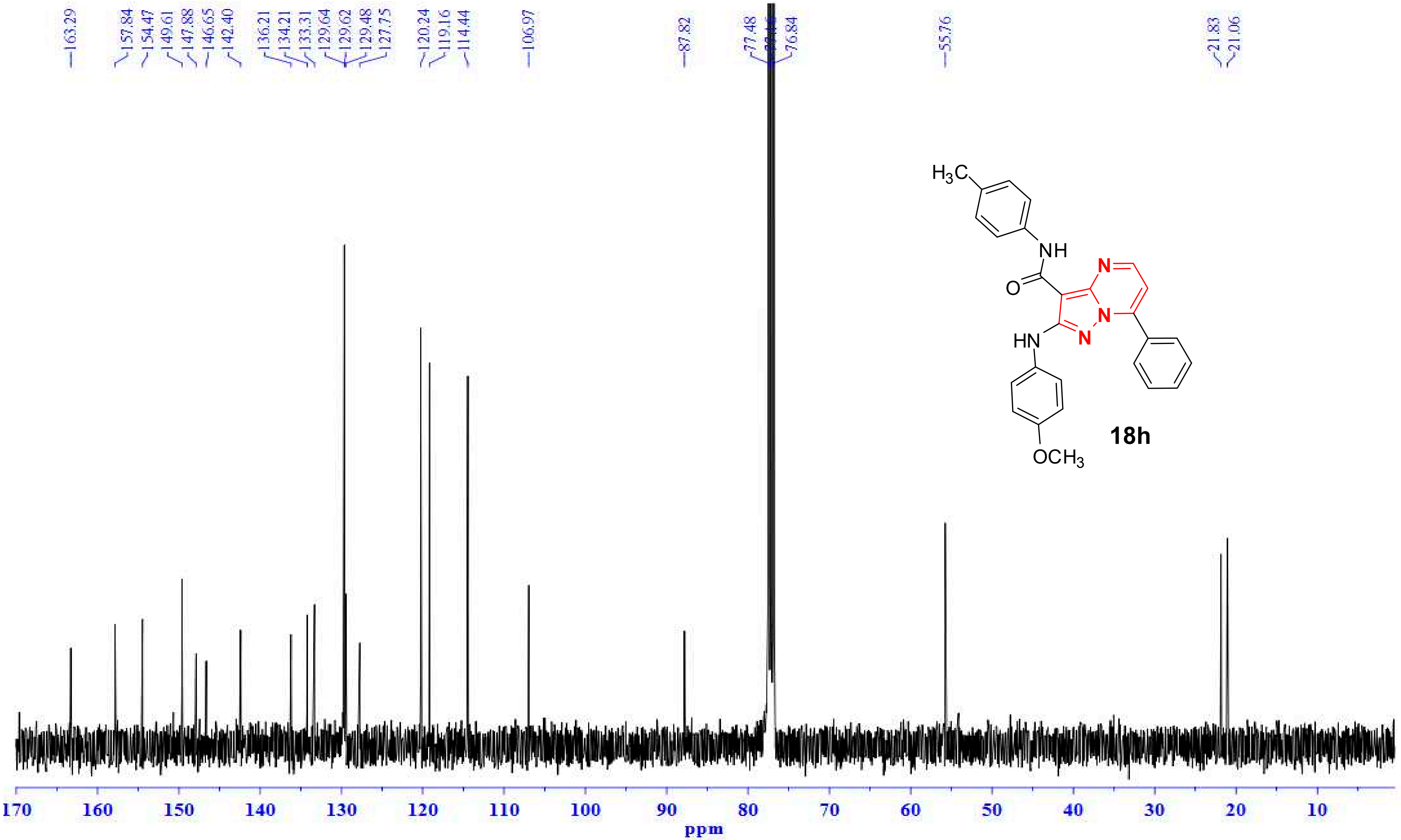

The ^{13}C NMR (100 MHz) spectrum of compound **18h**

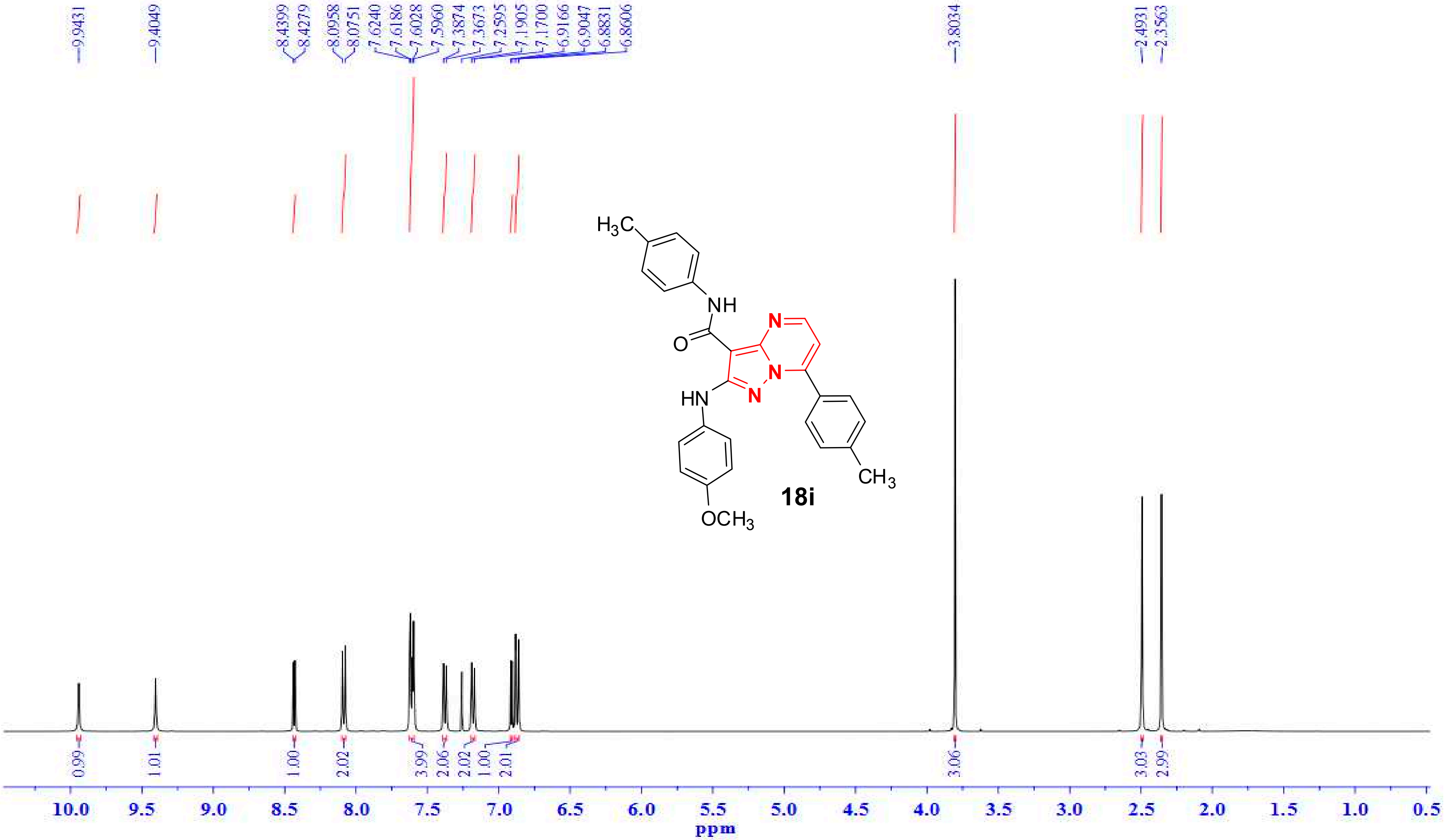

The ^{1}H NMR (400 MHz) spectrum of compound **18i**

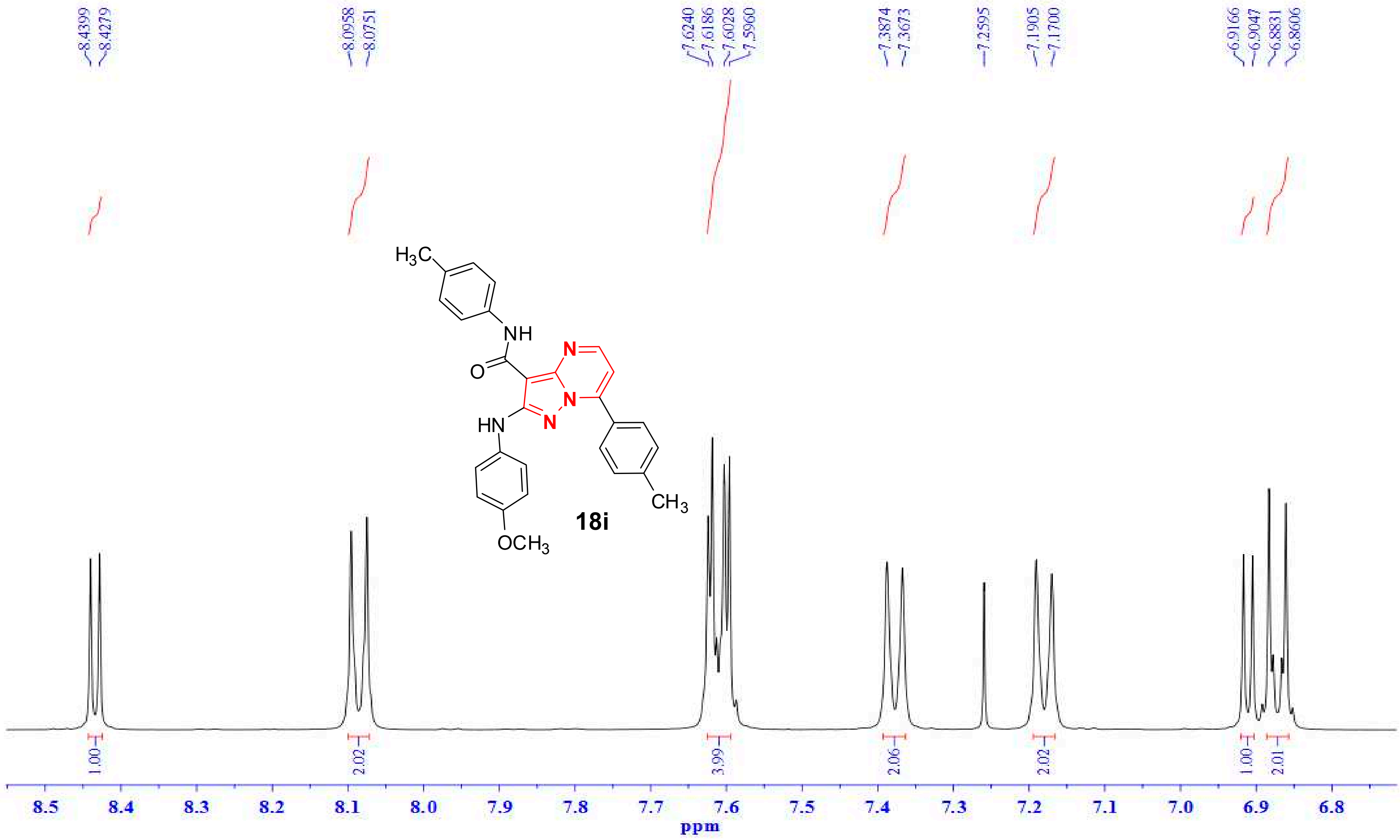

The ^{1}H NMR (400 MHz) spectrum (aromatic region) of compound **18i**

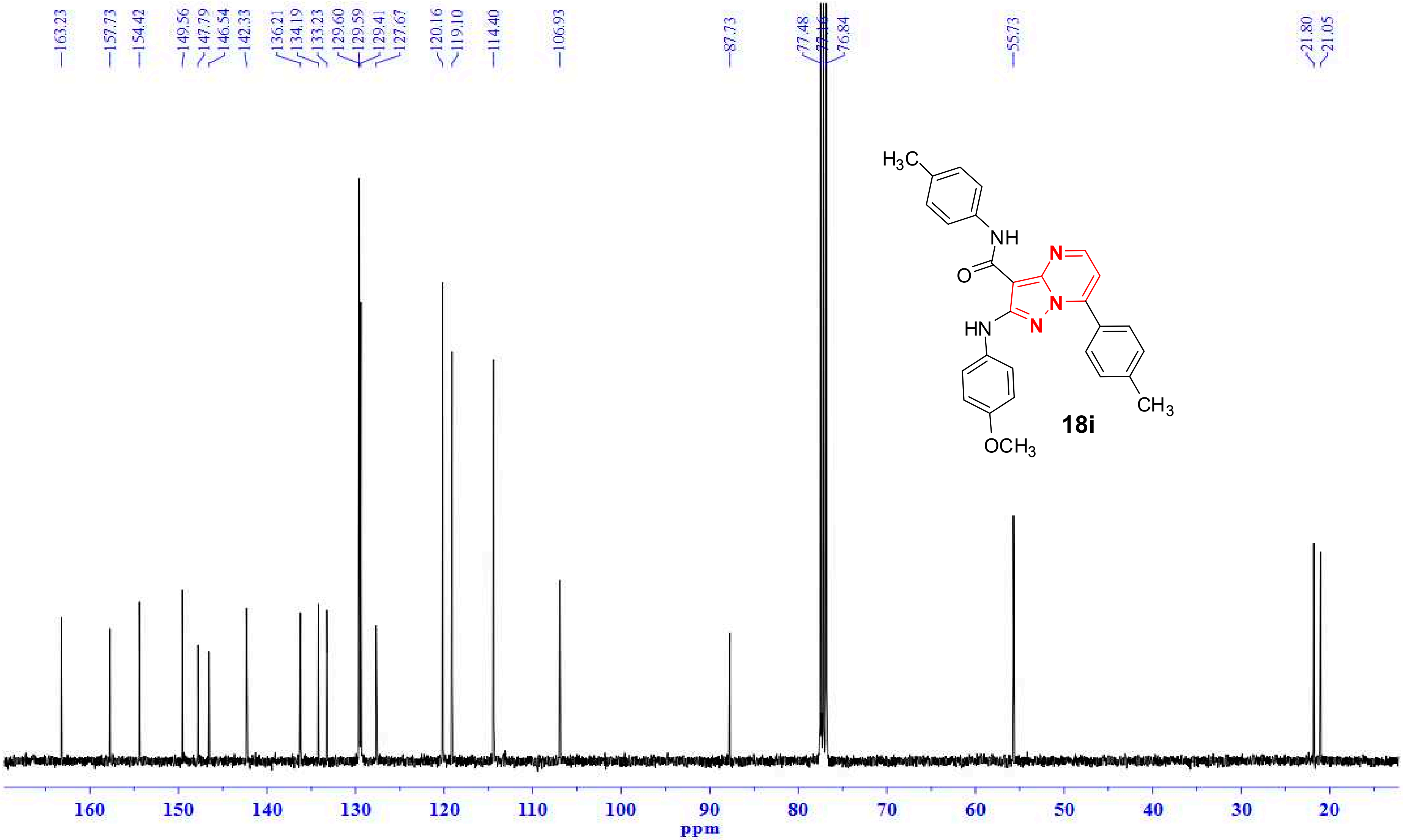

The ^{13}C NMR (100 MHz) spectrum of compound **18i**

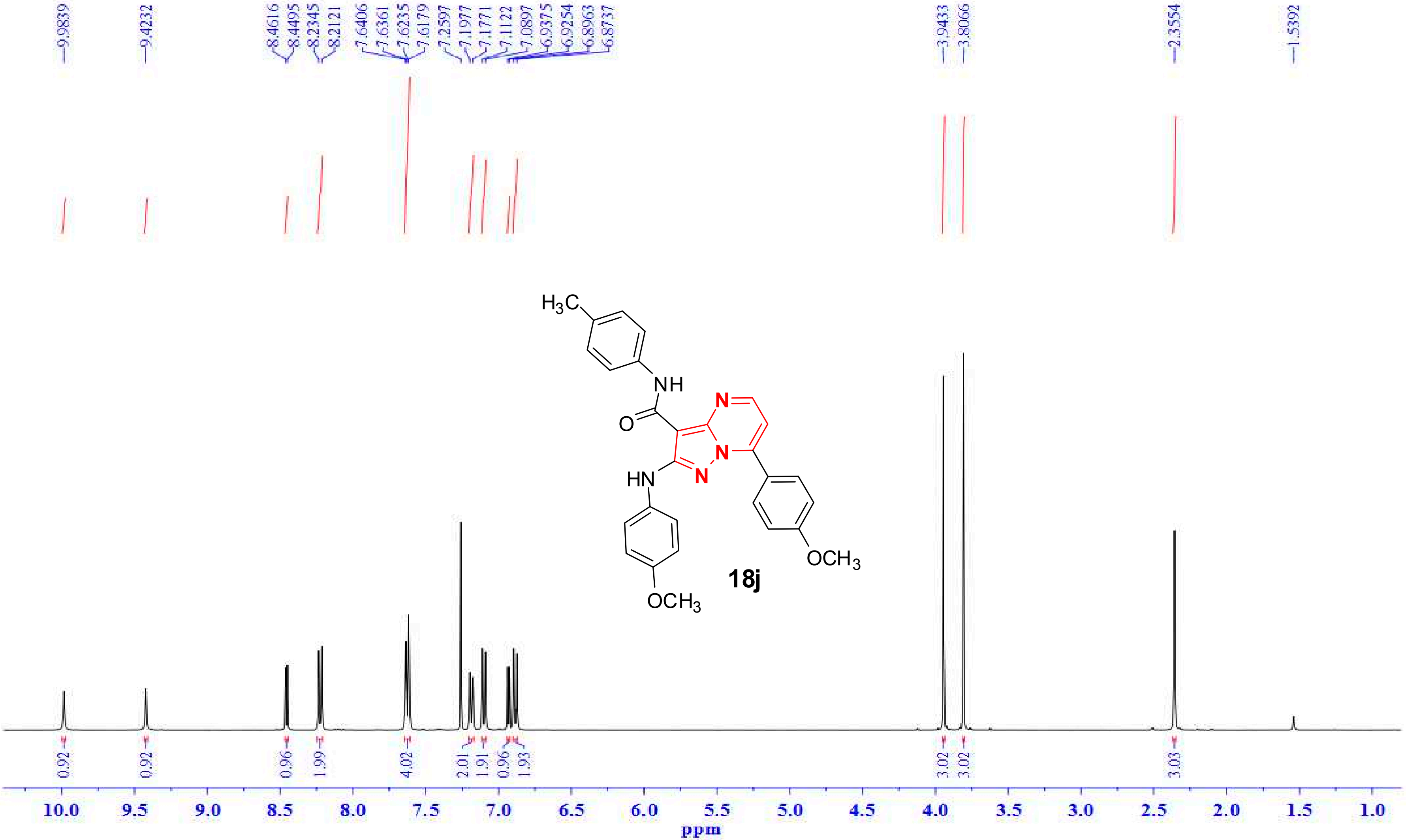

The ^{1}H NMR (400 MHz) spectrum of compound **18j**

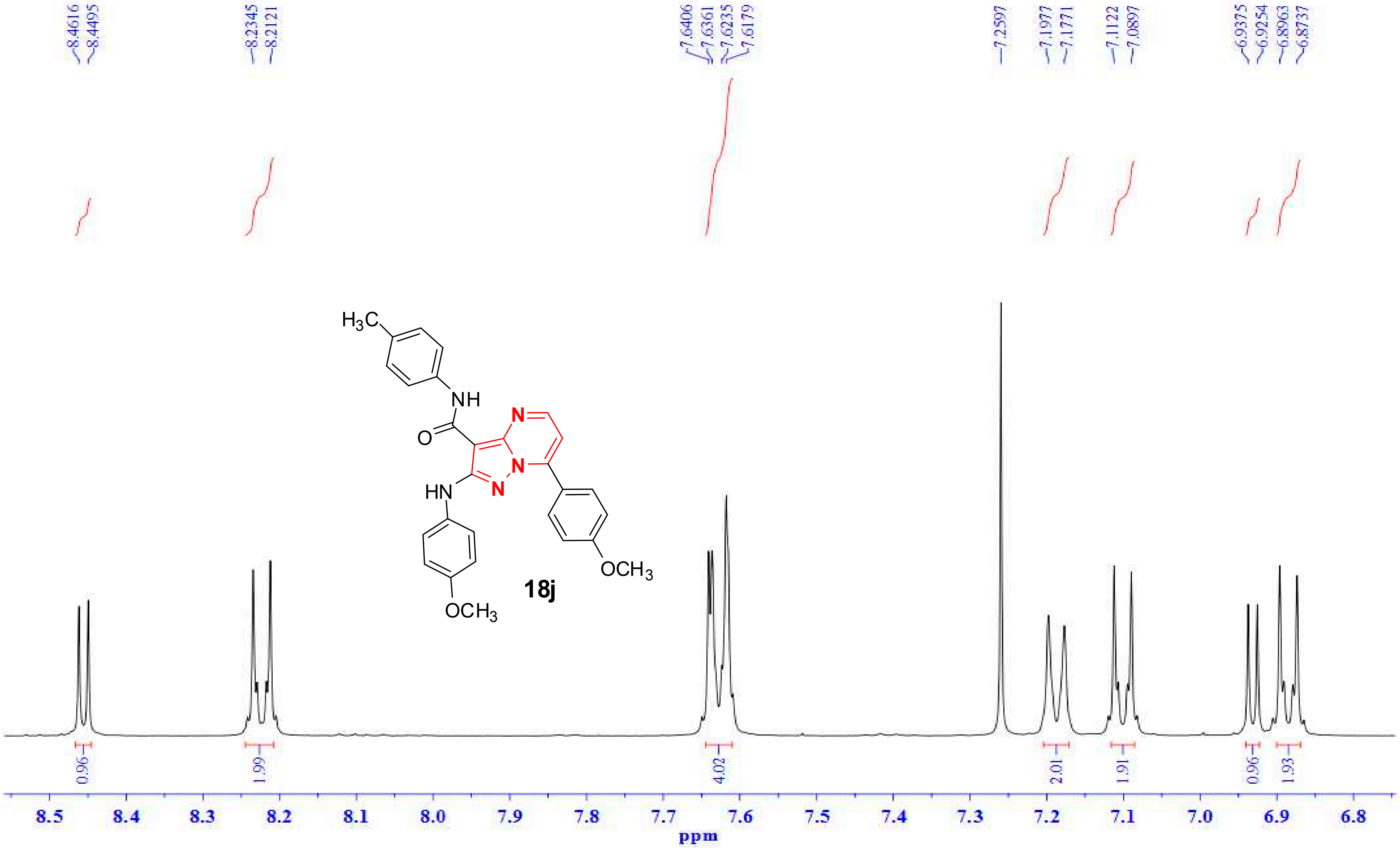

The ^{1}H NMR (400 MHz) spectrum (aromatic region) of compound **18j**

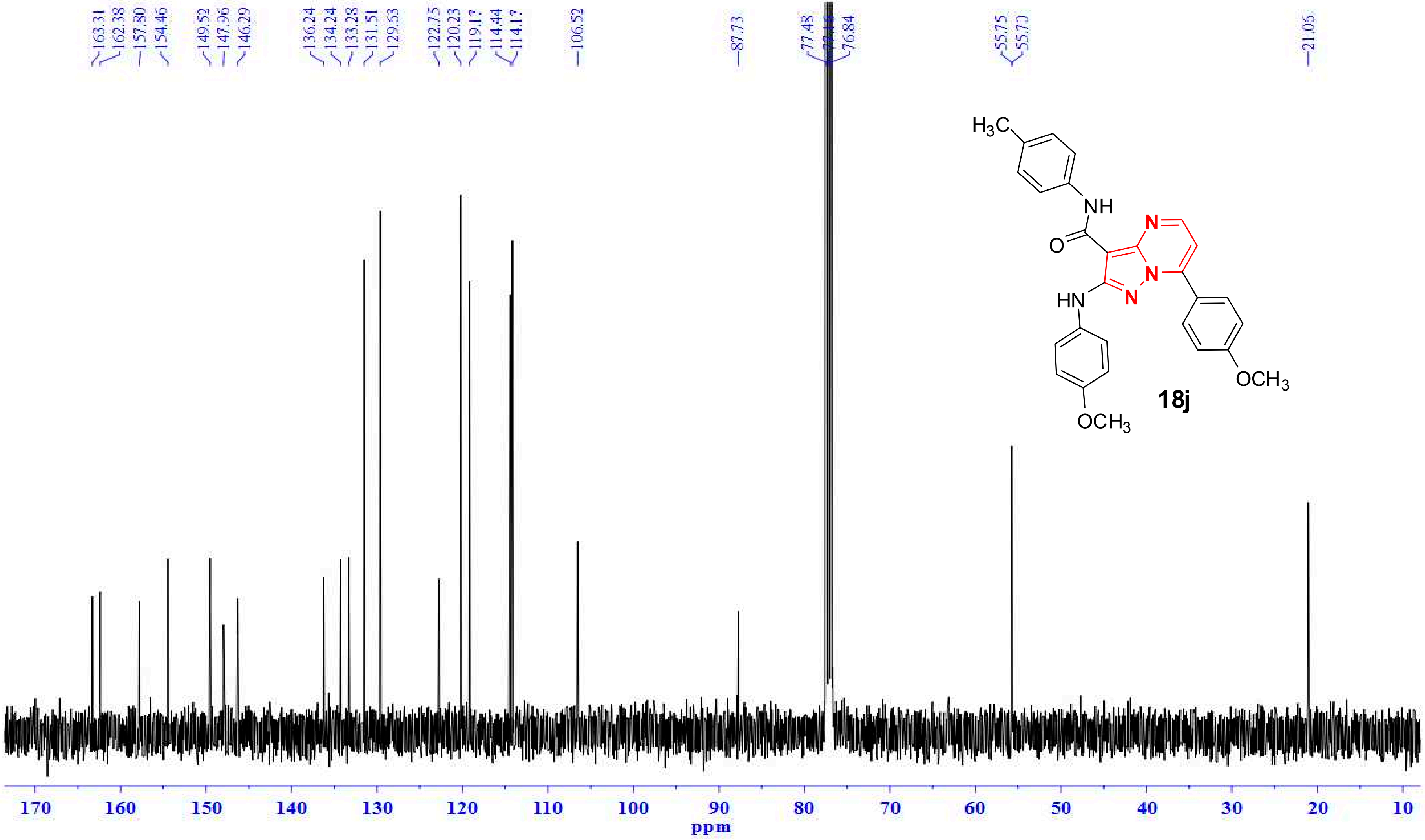

The ^{13}C NMR (100 MHz) spectrum of compound **18j**

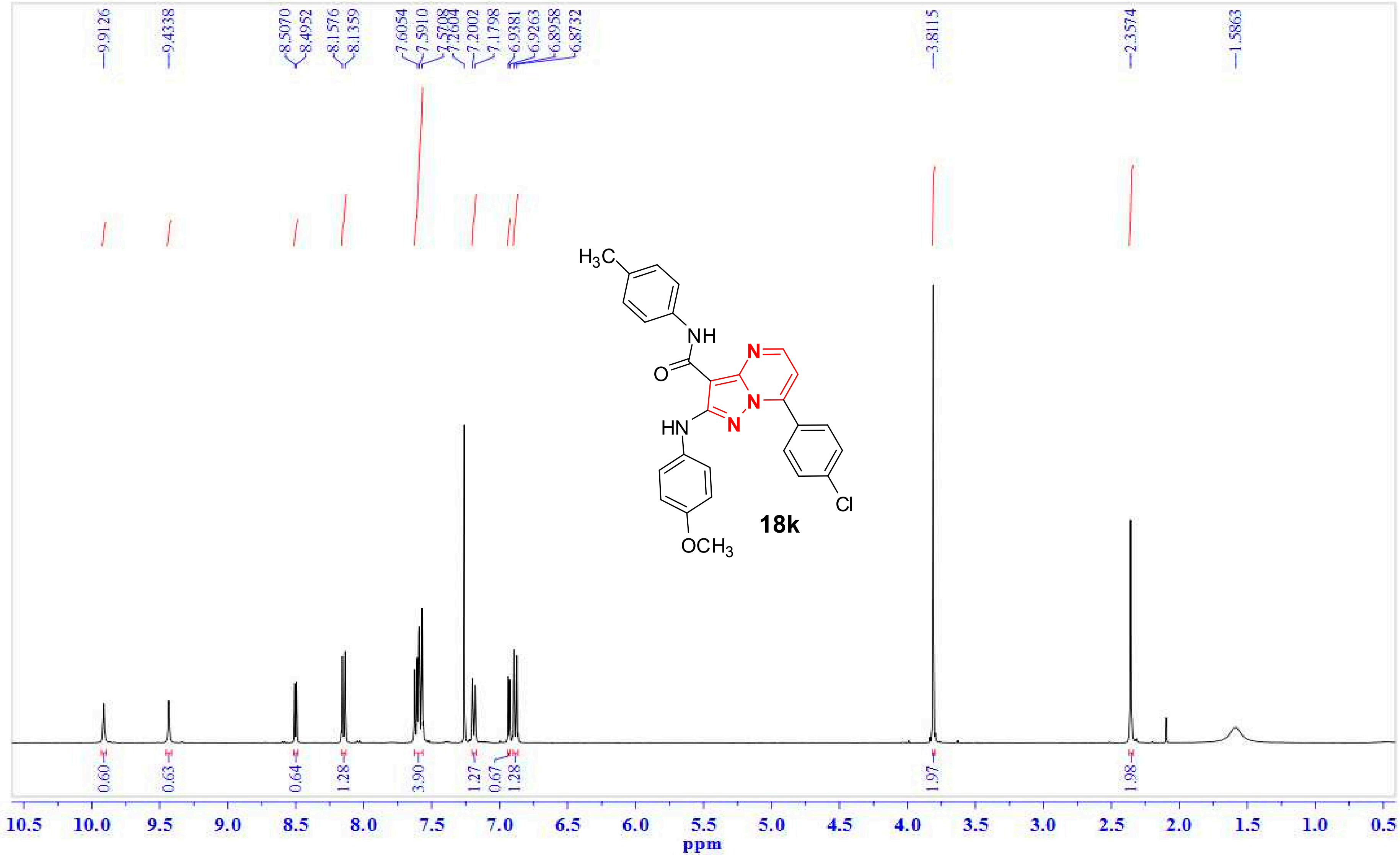

The ^{1}H NMR (400 MHz) spectrum of compound **18k**

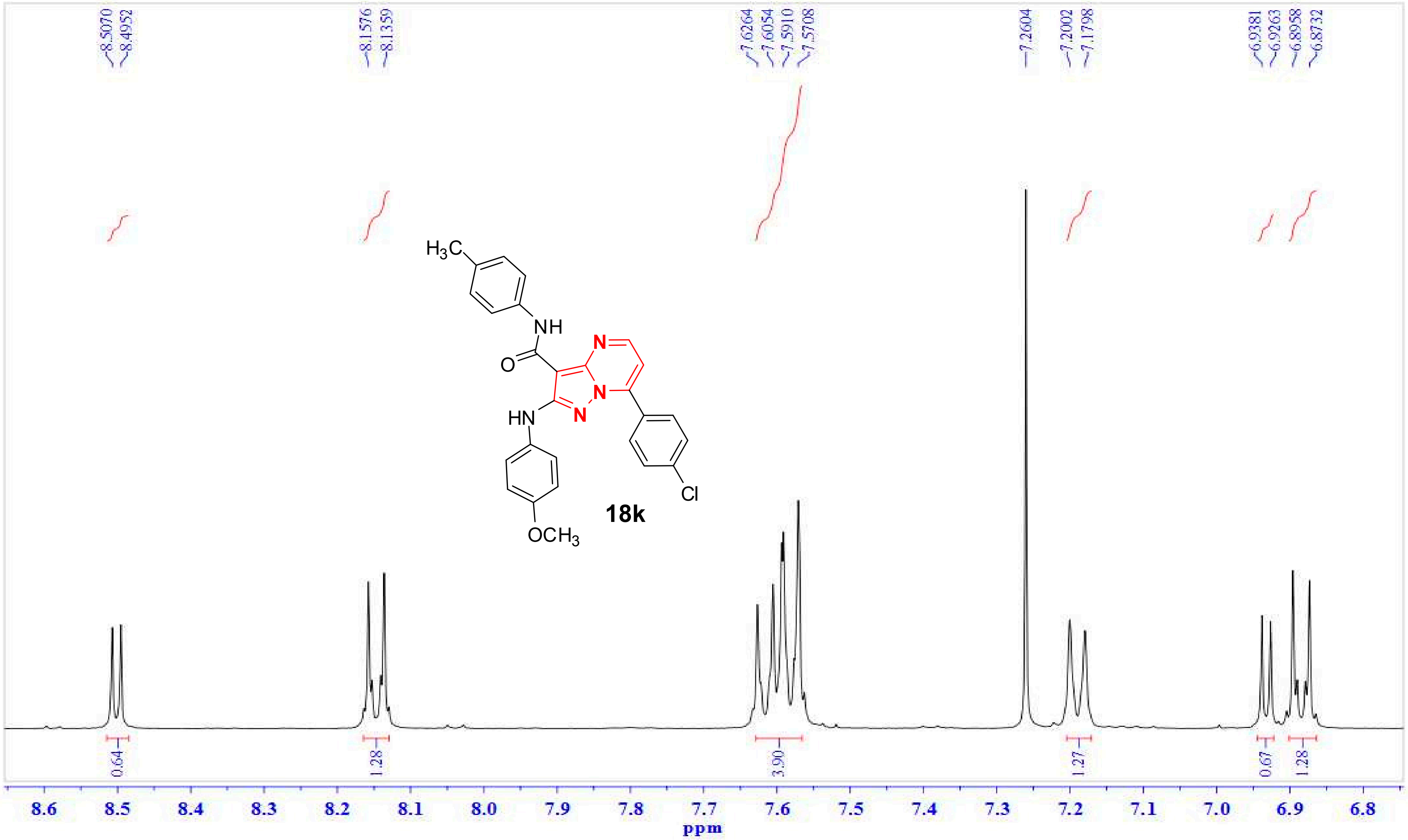

The ^{1}H NMR (400 MHz) spectrum (aromatic region) of compound **18k**

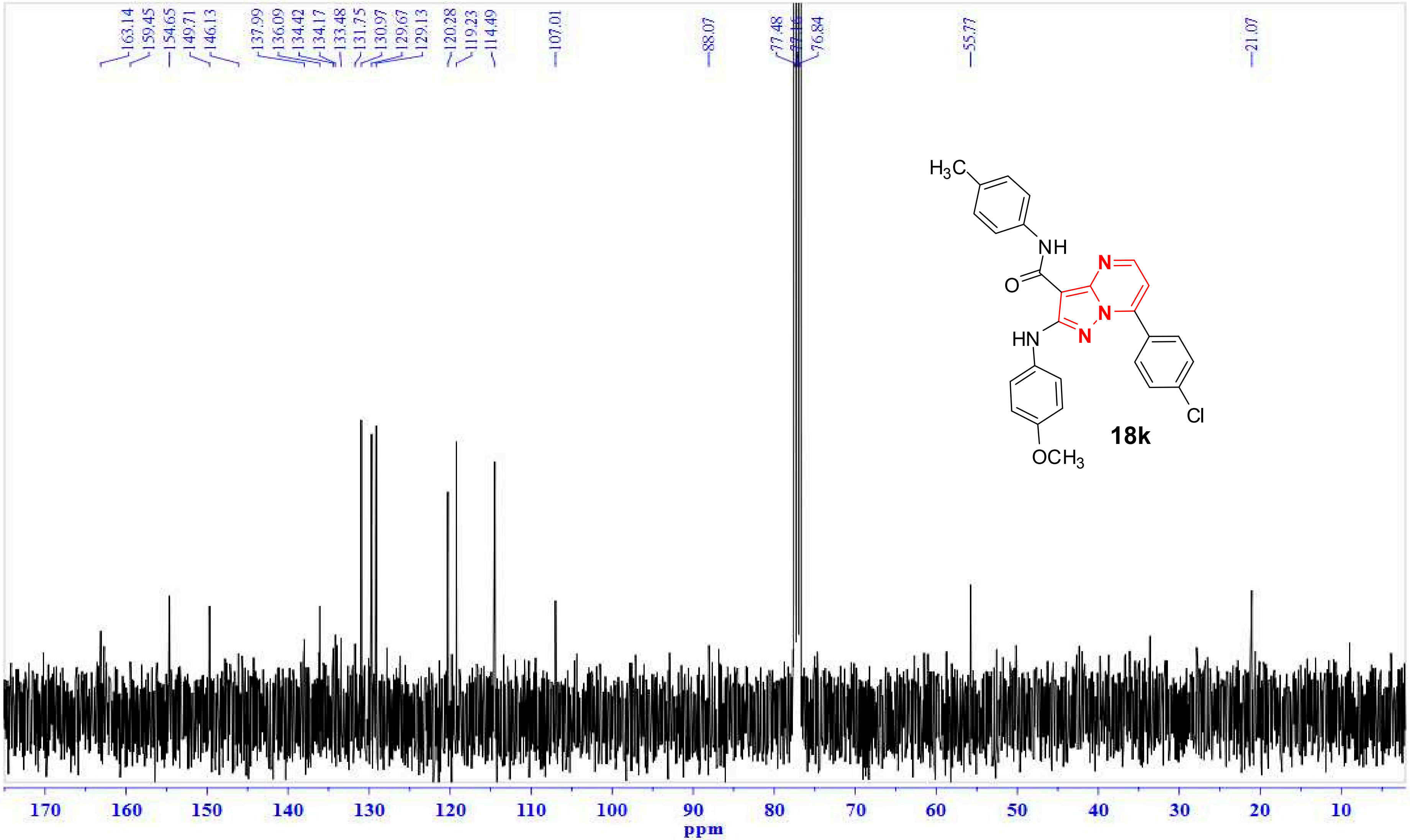

The ^{13}C NMR (100 MHz) spectrum of compound **18k**

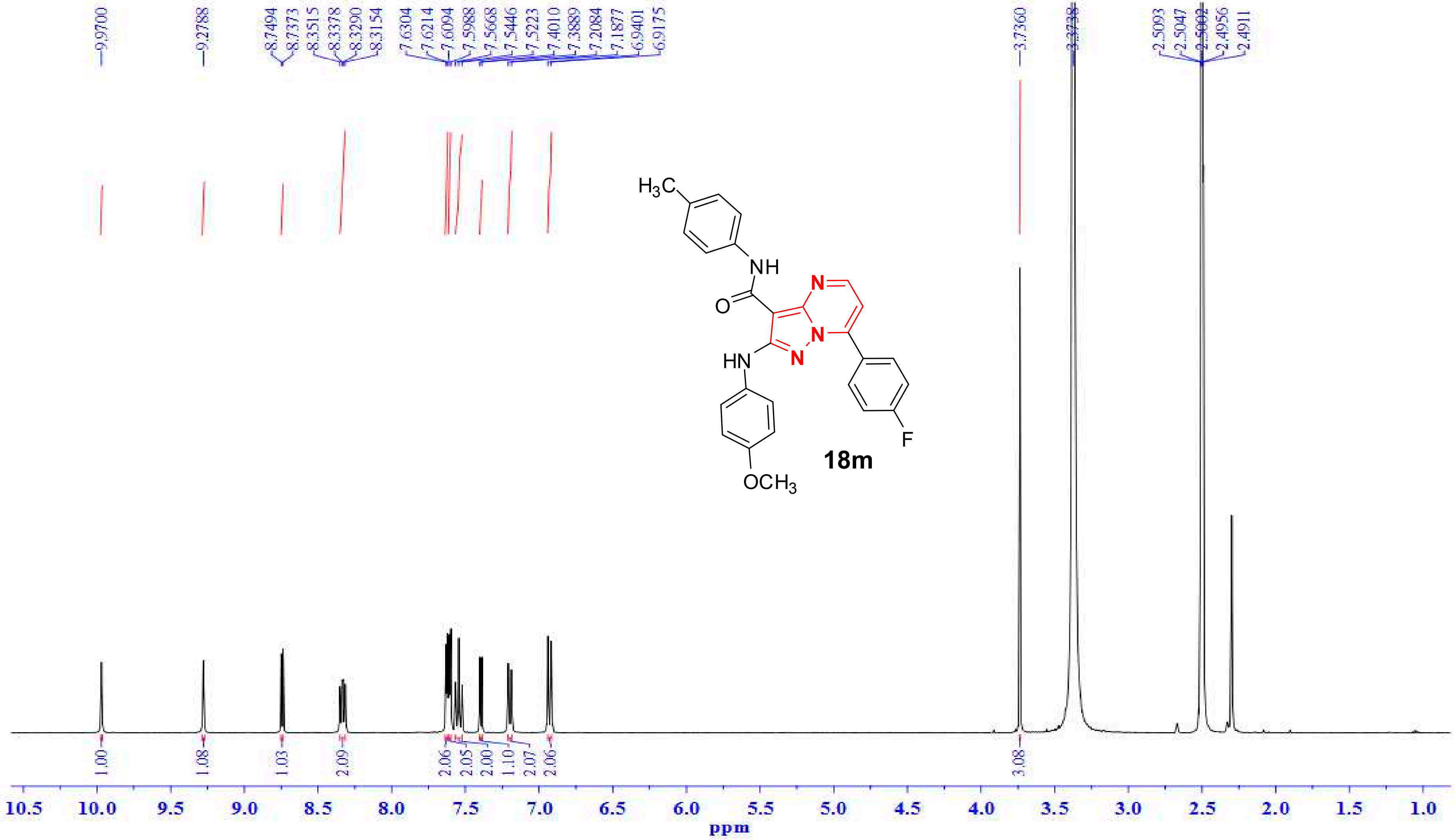

The ¹H NMR (400 MHz) spectrum of compound **18m**

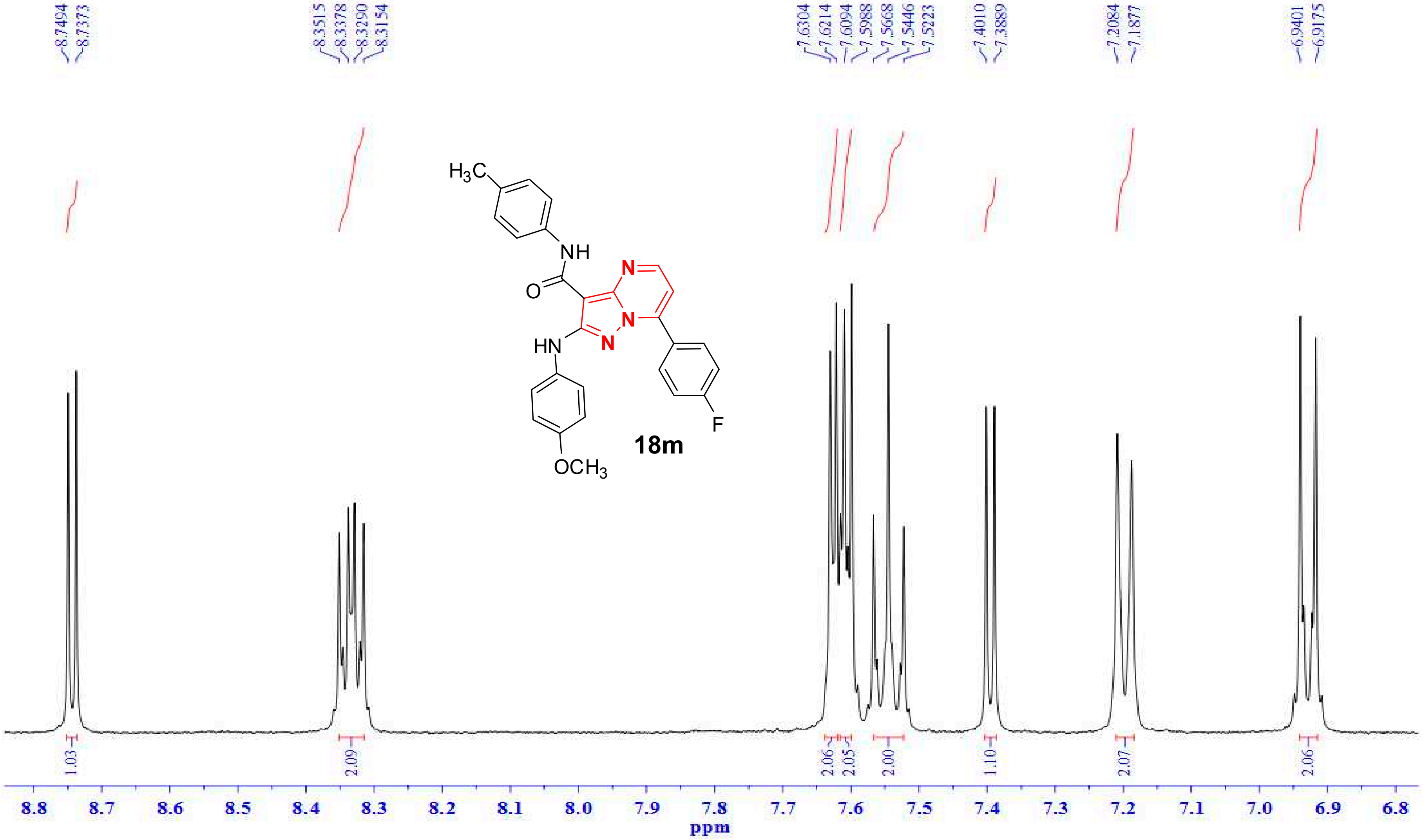

The ^{1}H NMR (400 MHz) spectrum (aromatic region) of compound **18m**

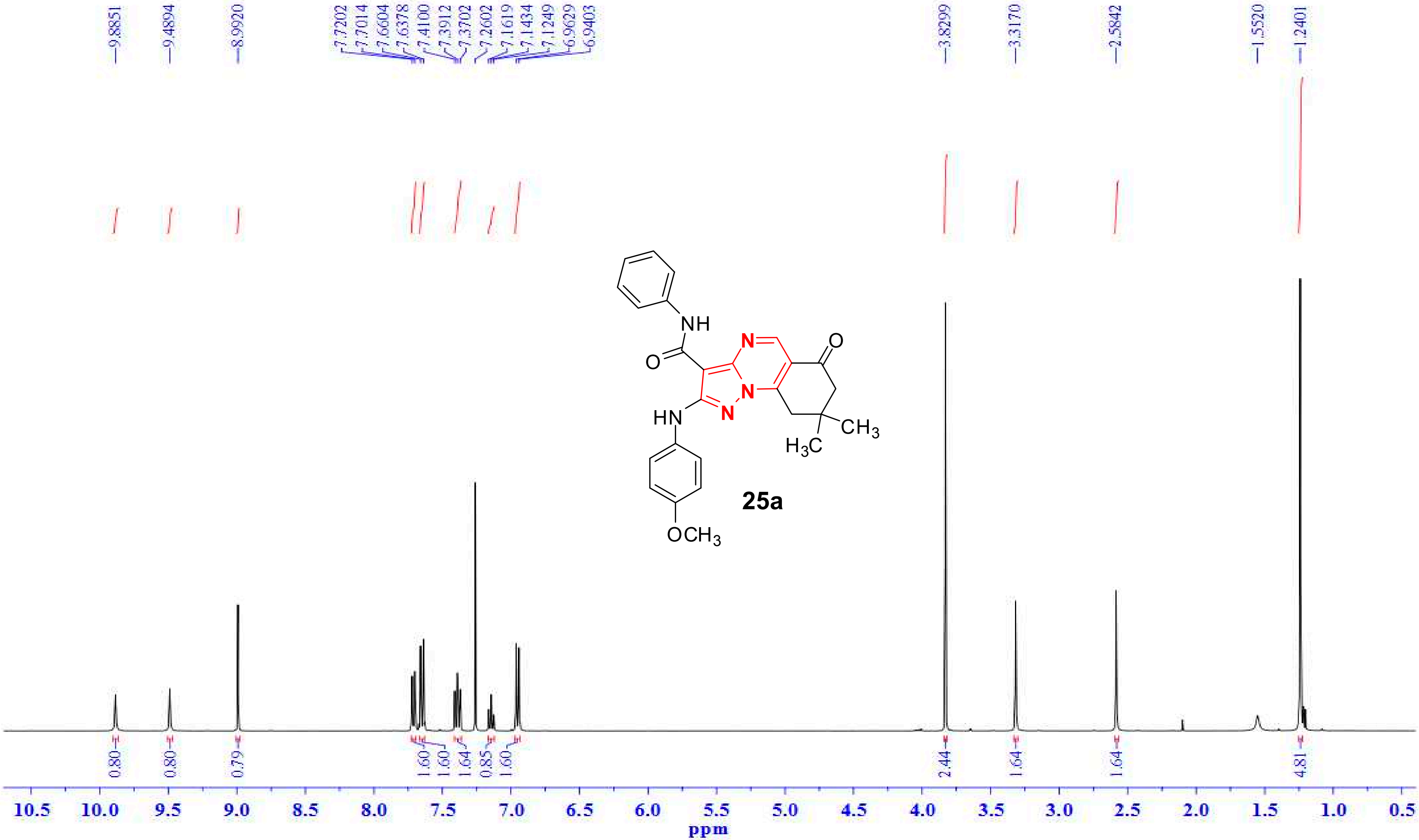

The ^{1}H NMR (400 MHz) spectrum of compound **25a**

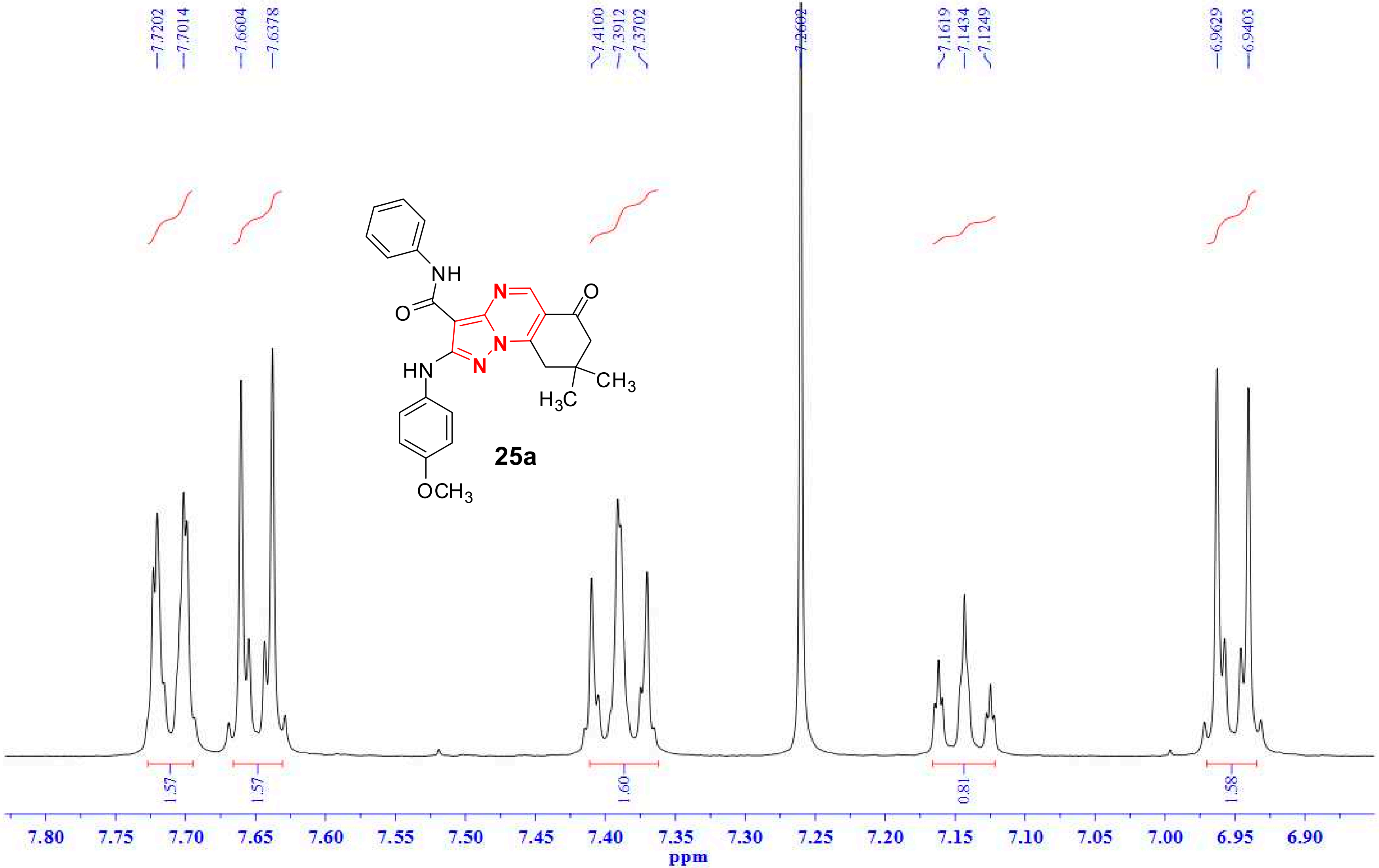

The ^{1}H NMR (400 MHz) spectrum (aromatic region) of compound **25a**

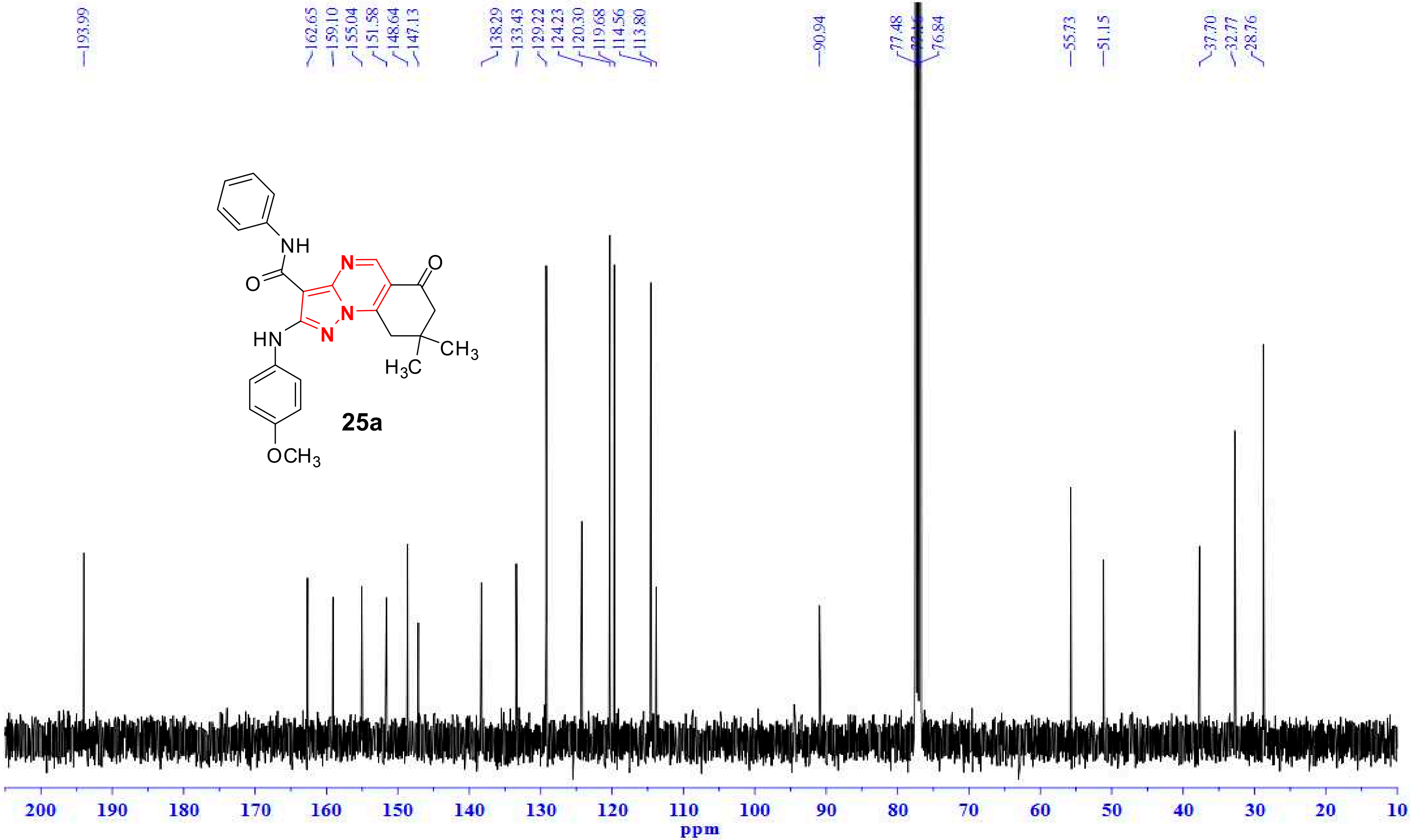

The ^{13}C NMR (100 MHz) spectrum of compound **25a**

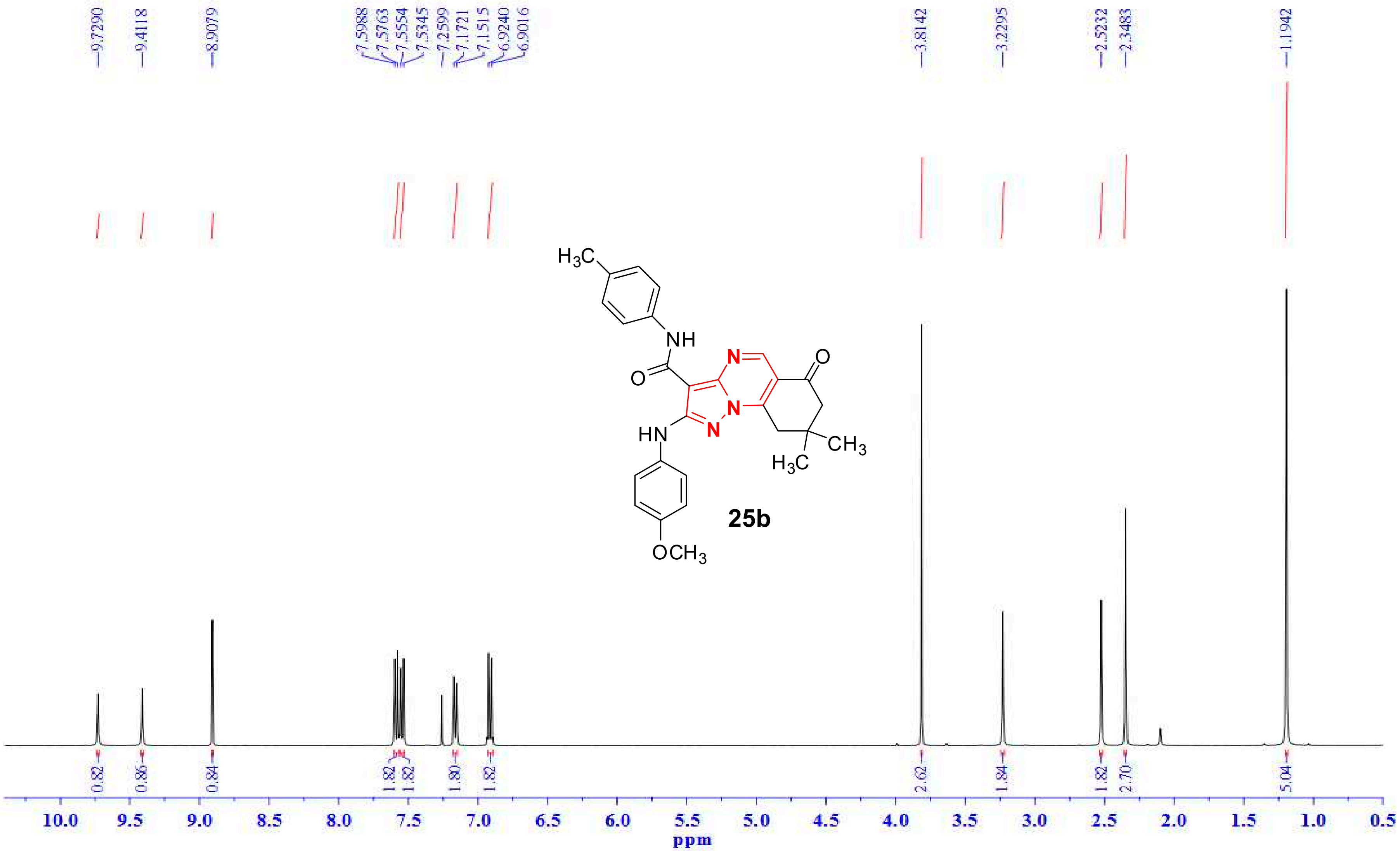

The ^{1}H NMR (400 MHz) spectrum of compound **25b**

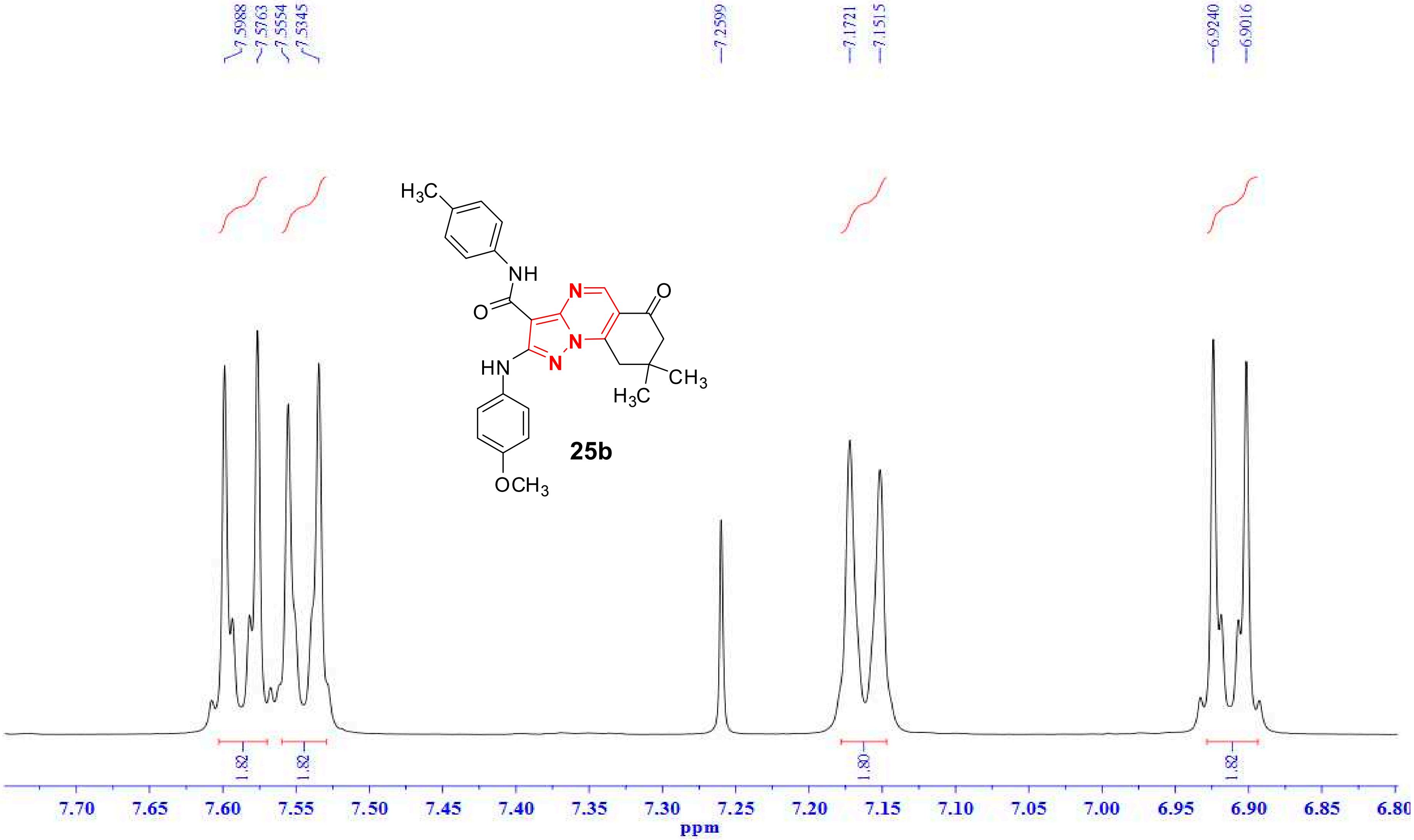

The ^{1}H NMR (400 MHz) spectrum (aromatic region) of compound **25b**

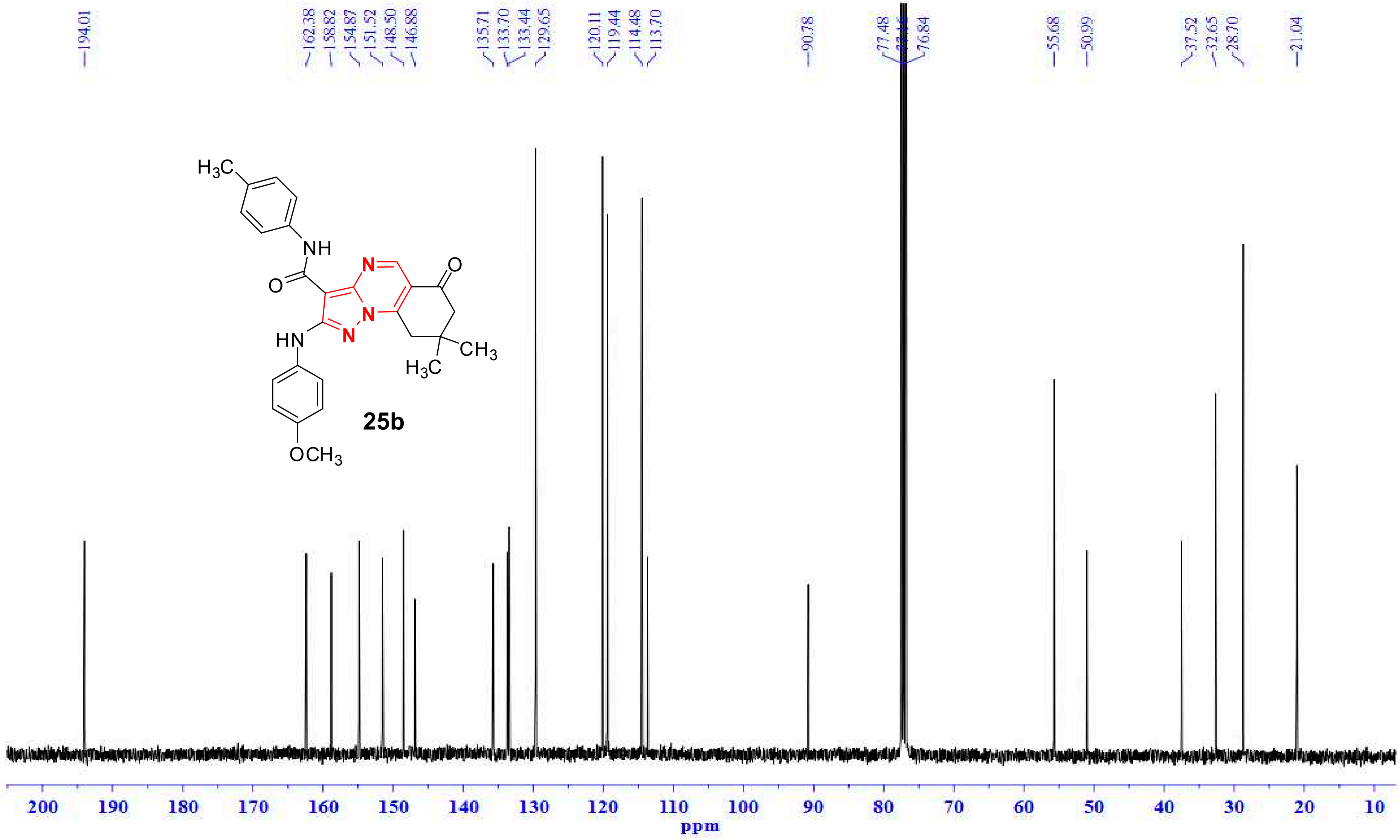

The ^{13}C NMR (100 MHz) spectrum of compound **25b**

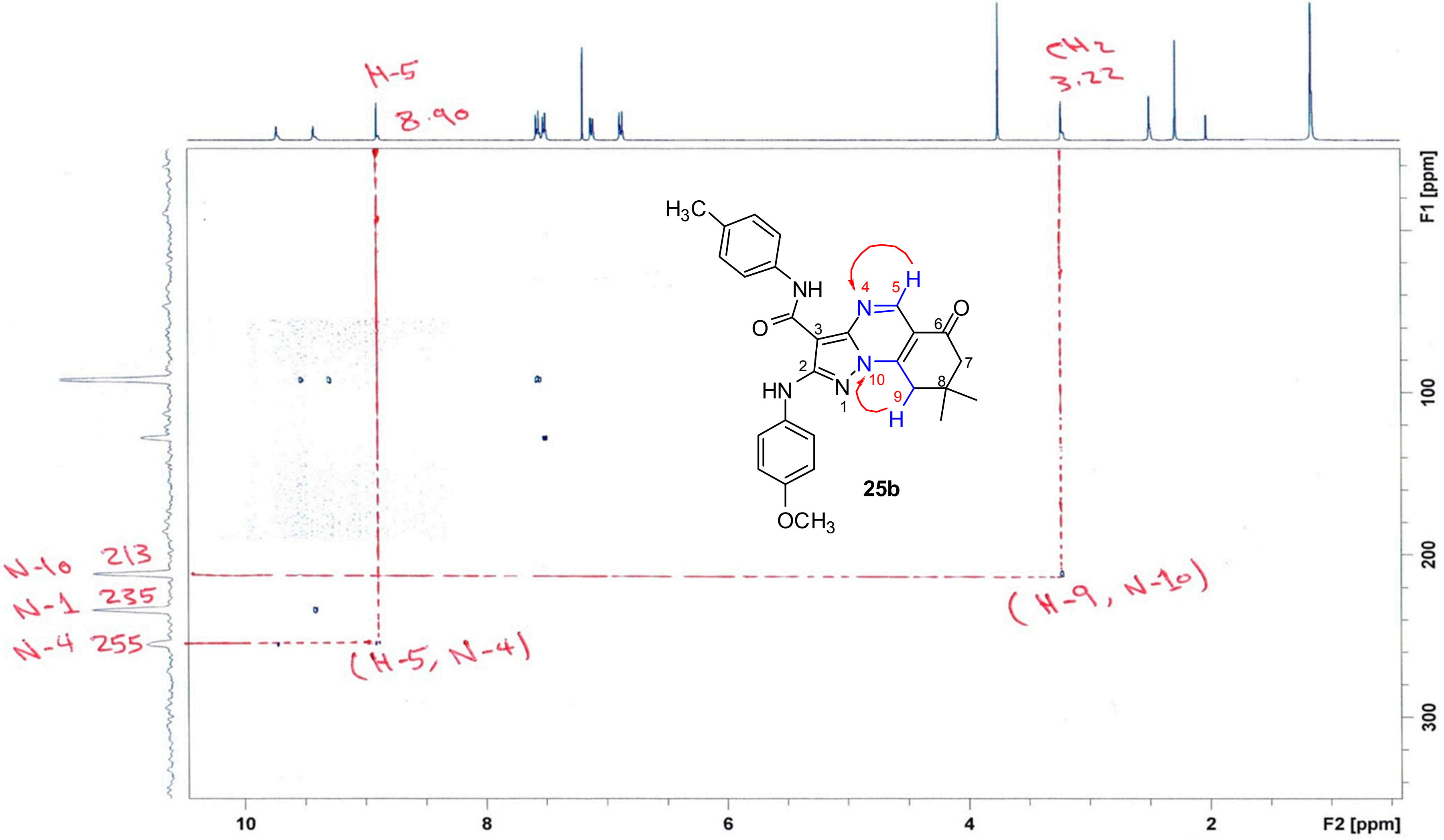

The ^{1}H-^{15}N HMBC (40 MHz) spectrum of compound **25b**
